# Evidence for an Electronically Driven Charge Density Wave in a 1D Metallic MOF

**DOI:** 10.1021/acscentsci.6c00405

**Published:** 2026-05-05

**Authors:** Jewel Ryu, Lukas Sippach, Sebastian A. Hallweger, Lukáš Palatinus, Peter Müller, Konstantin Glazyrin, Gregor Kieslich, Julius J. Oppenheim, Mircea Dincă

**Affiliations:** † Department of Chemistry, 2167 Massachusetts Institute of Technology, 77 Massachusetts Avenue, Cambridge, Massachusetts 02139, United States; ‡ Department of Chemistry, 6740 Princeton University, Washington Road, Princeton, New Jersey 08540, United States; § Technical University of Munich, 9184 TUM School of Natural Sciences, 85748, Lichtenbergstraße 4, 85748 Garching, Germany; ⊥ Institute of Inorganic and Analytical Chemistry, Justus Liebig University Giessen, Heinrich-Buff-Ring 17, 35392 Giessen, GER; ★ Institute of Physics of the Czech Academy of Sciences, Na Slovance 1999/2, 18200 Prague, Czechia; # 28332 Deutsches Elektronen-Synchrotron DESY, Notkestr. 85, 22607 Hamburg, Germany

## Abstract

Charge density wave (CDW) phases are unconventional quantum states that often arise in low-dimensional metallic systems and are themselves found alongside other exotic phenomena. Finding such states in porous materials is exceedingly rare. In fact, [Ln­(NO_3_)_1–*x*_]_3_(HOTP)_2_ (Ln = La, Nd; H_6_HOTP = 2,3,6,7,10,11-hexahydroxytriphenylene; **LnHOTP**) is the only example of a porous material wherein a CDW state has been proposed on the basis of a structural modulation. However, whether the modulation is trivial, and thus purely structural in nature, or it stems from a CDW, and thus has electronic origin, remains unknown. Here, low-temperature and high-pressure crystallography provide evidence for an electronic origin of the CDW phase in a series of **LnHOTP** (Ln = La, Ce, Pr, Nd, and Sm) MOFs, including the original La and Nd materials. We show that the modulation affects the relative rotation of neighboring HOTP ligands, and that the magnitude of the wavevector that defines the modulation, *q*, is sensitive to pressure. Importantly, the wavevector exhibits commensurability lock-in at one-third of the *c* unit cell parameter, *q* = ^1^/_3_ *c*, providing key evidence for energetic stabilization of the CDW phase.

## Graphical abstract

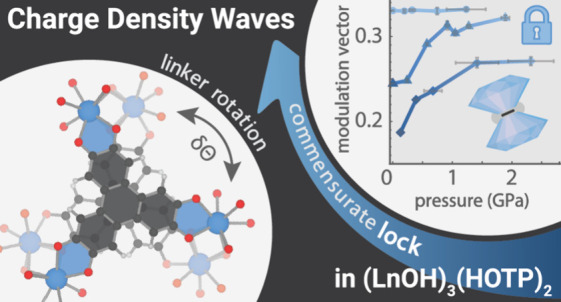

## Introduction

In low-dimensional metallic systems, the Peierls effect causes a spontaneous lattice distortion that is coupled to a modulation of the electron density. The structural deformation opens a gap in the conduction band to achieve electronic stabilization, leading to charge density wave (CDW) fluctuations.^1,2^ These phases are of interest as they often point to other potential exotic electronic states, including superconductivity.^3,4^

Among CDW materials, **LnHOTP** [Ln­(NO_3_)_1–*x*_]_3_(HOTP)_2_ (Ln = La, Nd; H_6_HOTP = hexahydroxytriphenylene) is unique in that it is a metal–organic framework (MOF).^5^ By virtue of their compositional and structural tunability, MOFs that host CDW phases will be of interest to a broader scientific community. Establishing an electronic origin for this first example of CDW in a MOF is critical.^1–5^

**LnHOTP** frameworks can be described as hexagonal two-dimensional lattices of HOTP ligands connected in the third dimension by one-dimensional (1D) secondary building units (SBUs) made from lanthanide-catechol bonds, wherein lanthanide atoms display square antiprismatic or dodecahedral geometries.^5–8^ We showed previously that the SBUs, formed entirely by highly ionic Ln–O bonds, do not contribute to electronic transport in these materials.^5–7^ Instead, one-dimensional π-stacks of HOTP ligands mediate true, temperature-deactivated metallic conductivity in the crystallographic *c* direction. The La and Nd systems also exhibited periodic lattice modulations below a critical temperature of 361–365 K.^5^ We associated this behavior with a CDW transition, as would be expected for 1D metallic systems. As the electronic instability is coupled to the distortion of the lattice, obtaining the crystal structure is paramount to understanding the effects of the modulation. However, the structures below the phase transition remained elusive, limiting our interpretation on the origin of the CDW. Assigning the proper space group for each case requires a rigorous examination of the periodic lattice modulations and various disorders inherent in the structure.^9^ This motivates a thorough investigation of the modulated structure of the CDW—as well as potential lock-in-transitions—within the **LnHOTP** series. To unequivocally establish the CDW as having an electronic origin, we set out to examine the effects of the modulation in **LnHOTP** across a range of temperatures and pressures.

Through a rigorous crystallographic study of **LnHOTP** at low temperatures and high pressures, we provide strong evidence for an electronically driven CDW in the original La and Nd MOFs. Our conclusions are further strengthened by expanding the material scope to the full series of the early lanthanides (Ln = La, Ce, Pr, Nd, and Sm) and examining the structure across a wide range of modulation wavevectors, *q*. The preparation of high-quality single crystals is essential, as only crystals that diffract with strong satellite peaks are capable of revealing key information associated with the phase transition. A comparison of crystals with different modulation wavevectors—including both commensurate and incommensurate cases—further provides incisive clues to the role of commensurability on the energetic stabilization of the CDW. We present the various flavors of diffuse scattering observed in this system and delineate the types of local correlations in structural disorder of the Ln chains that give rise to these differences. From La to Nd, we demonstrate that the crystals are isostructural regardless of the identity of the lanthanide (Figure 1a, b). Although the modulation wavevector appears to be temperature-invariant, we note a pressure dependence of the wavevector in the range of 0–2.5 GPa for incommensurate crystals. We further remark on a commensurability lock-in at *q* = ^1^/_3_ *c* that points to an energetic stabilization. Through a comprehensive analysis of the crystal structure, we lay the groundwork for underpinning the origins of the CDW in the **LnHOTP** series.

**Figure 1:**
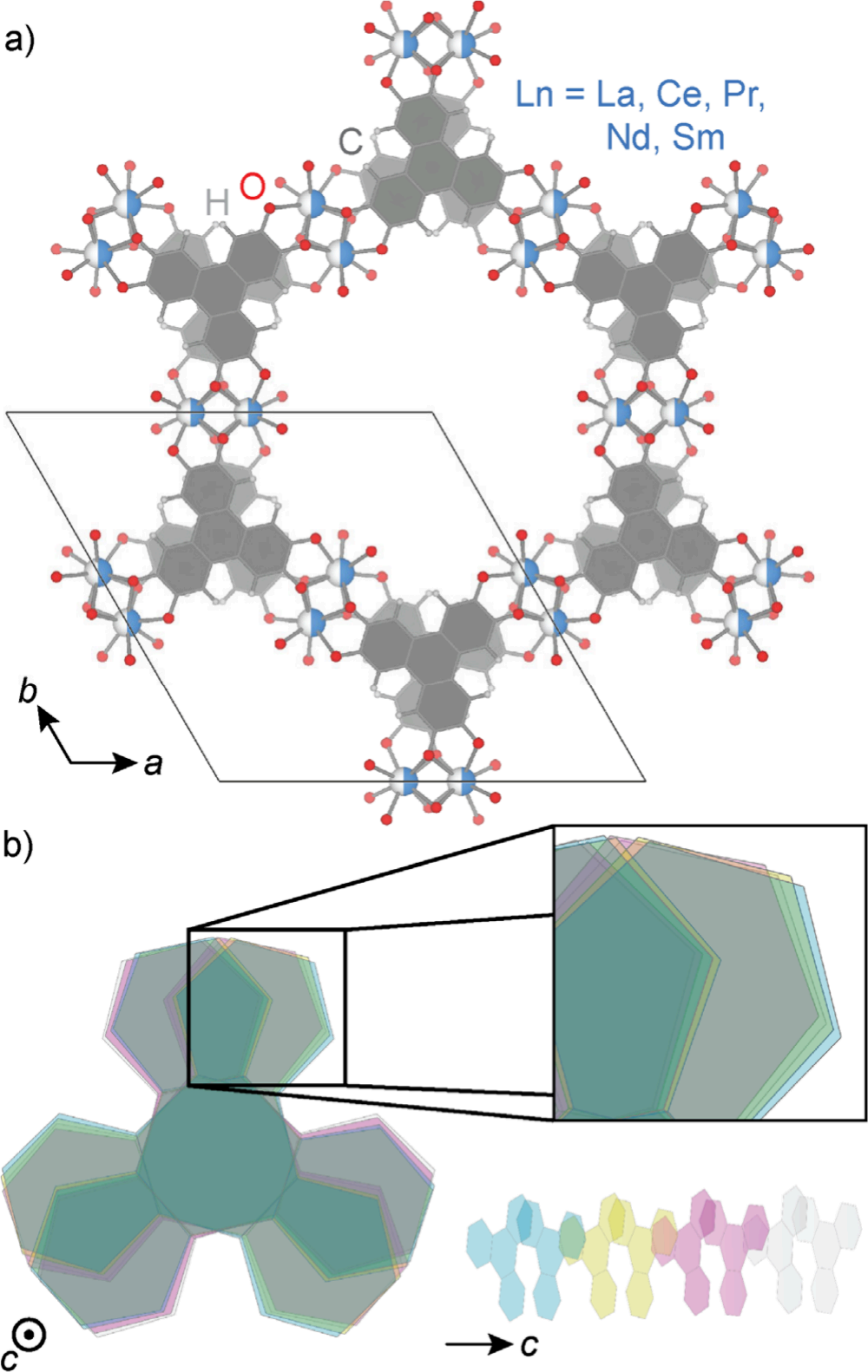
(a) Schematic illustrating the general structure of hexagonal phase **LnHOTP**. (b) A periodic lattice modulation can be observed along the crystallographic *c*-axis. Inset shows a magnified view of the relative rotation of neighboring HOTP molecules.

## Results and Discussion

### Materials Synthesis

Crystals of [Ln­(NO_3_)_1–*x*_]_1.5_HOTP·*n*H_2_O (Ln = La, Ce, Pr, Nd, and Sm; denoted as **LnHOTP**) were synthesized by the solvothermal reaction of H_6_HOTP and Ln­(NO_3_)_3_·6H_2_O in a solution of deionized water and *N*,*N*-dimethylacetamide (DMA) at 135 °C for 3 days. Powder X-ray diffraction (PXRD) patterns demonstrate high crystallinity and phase purity for all samples (). Elemental analysis indicates a formula that agrees with [Ln­(NO_3_)_1–*x*_]_1.5_HOTP·*n*H_2_O (0.39 ≤ *x* ≤ 0.65, 22 ≤ *n* ≤ 28). These conditions mimic previously reported procedures for **LaHOTP** and **NdHOTP**,^5^ with subtle differences in the crystallization (i.e., addition of 0.1 M hydrochloric acid modulator) and workup process (immediate exchange of the mother liquor after reaction cooling to prevent nucleation of impurity phases at room temperature). Additionally, we note that high quality crystals of **LnHOTP** can only be grown from fully reduced H_6_HOTP (a colorless material, unlike the oxidized H_*x*_HOTP (*x* < 6), which can display colors ranging from yellow, blue, pink, purple, or black).

### The Average Structure of LnHOTP

Solving the full structure of **LnHOTP** is a crystallographic puzzle, in which we encounter periodic lattice modulations and various correlated disorders that contribute to strong diffuse scattering. Before delving into these technical details, we begin with a discussion on the average structure of **LnHOTP**. The average structure, which adopts the formula [Ln­(NO_3_)_1–*x*_]_6_(HOTP)_4_·*n*H_2_O, crystallizes in the *P*6/*mcc* space group. This structure serves as the parent average structure for all lower symmetry phases, as we discuss below. The average structure unit cell contains two 1D stacks of HOTP ligands bridged by three chains of lanthanides. The HOTP ligands stack in a nearly eclipsed manner with only a slight rotation about the *c*-axis, where two neighboring HOTP molecules along each stack are related by a *c* glide (Figure 1). Each Ln atom can be modeled as a square antiprism or a distorted dodecahedron, coordinated to six oxygen atoms from four individual HOTP ligands and two oxygen atoms from water and/or charge-balancing nitrate ions.

Although the average structure resembles the model proposed by Skorupskii et al. (refined from PXRD in *P*6*cc*),^6^ it is important to note that our average structure exhibits no disorder of the HOTP linkers. If occupational disorder of HOTP (in addition to the disorder of the Ln sites) were present, the symmetry would be *P*6/*mmm* (the supergroup of *P*6/*mcc* with *k*-index = 2) with the *c*-lattice parameter halved to ∼3 Å. However, we find no such crystallographic evidence of linker disorder.

In fact, the only disorder observed is at the Ln sites, which are each half-occupied. Although *uncorrelated* disorder of the Ln sites would halve the cell size of the Ln-sublattice in the *c*-direction, we herein observe a unique case of *correlated* disorder. Indeed, the occupancy of the Ln atoms is correlated with a strong tendency for alternate stacking along the *c*-axis (Figure 2c). The correlations are responsible for strong diffuse scattering that appear in the odd-*l* *hkl* planes. The form factor of the disordered structure causes diffuse scattering in the odd-*l* planes with systematic absences arranged in a hexagonal lattice. Remarkably, the spacing between these absences corresponds to the Ln···Ln distance of a disordered pair (∼3 Å).

**Figure 2:**
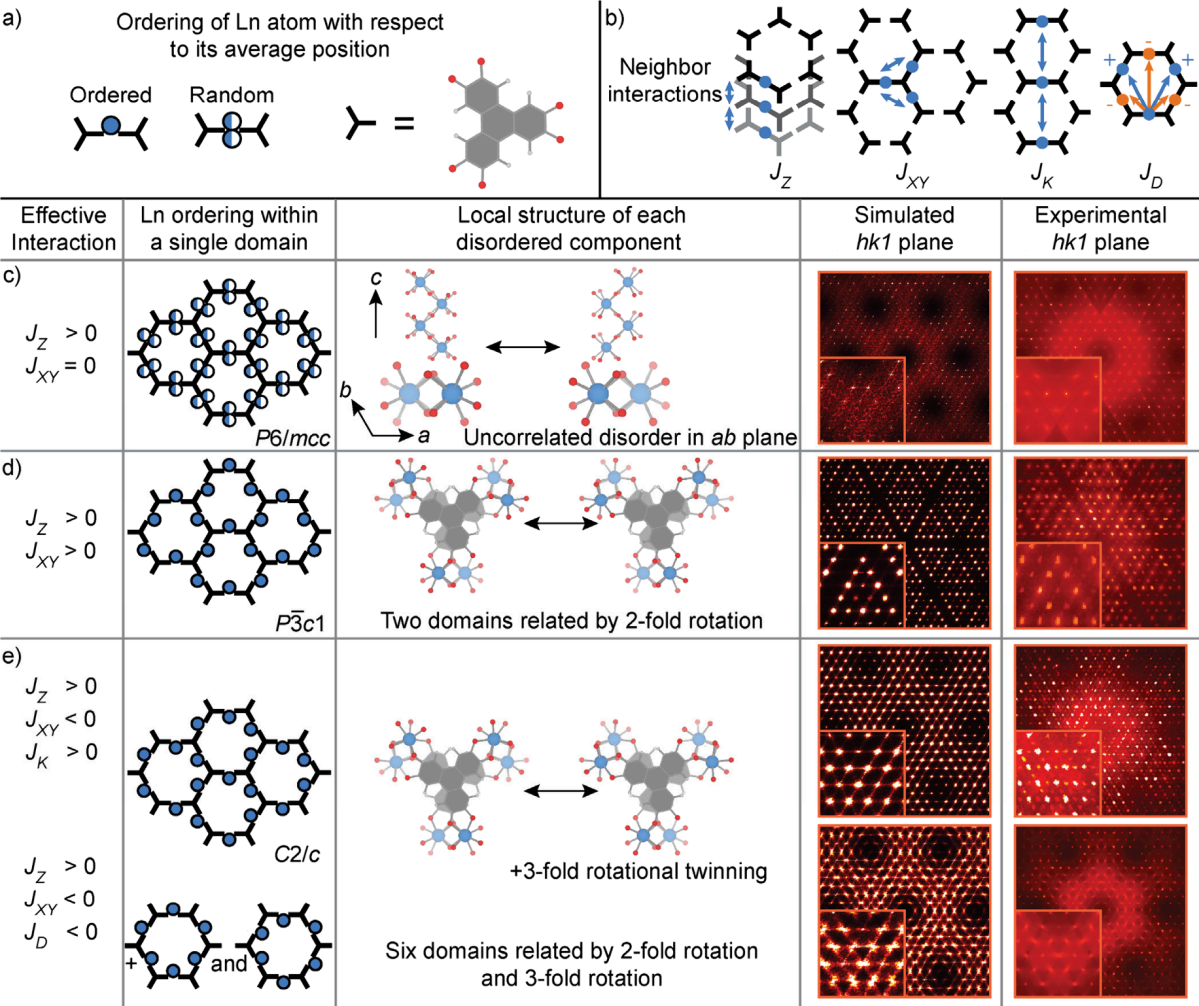
Illustration of diffuse scattering in the *hk1* plane. (a) Description of the representation for the Ln disorder. (b) Definition of the interaction types. *J*_*XY*_ defines the nearest neighbor interactions of a disordered Ln pair with its four nearest neighbors in the *ab*-plane. *J*_*Z*_ refers to nearest neighbor interaction of a disordered Ln pair with its two nearest neighbors along the *c*-axis. *J*_*K*_ and *J*_*D*_ correspond to additional cross-pore interactions of a disordered Ln pair. (c) Comparison of the simulation for *J*_*Z*_ = 100 with the synthesized precession image for a crystal of **NdHOTP** (with symmetry applied to the precession image). (d) Comparison of the simulation for *J*_*Z*_ = 100 and *J*_*XY*_ > 20 with the synthesized precession image for a crystal of **CeHOTP**. (e) Comparison of the simulations for either (top) *J*_*Z*_ = 100, *J*_*XY*_ = −5, and *J*_*K*_ = 5 or (bottom) *J*_*Z*_ = 100, *J*_*XY*_ = −1, and *J*_*D*_ = −100 with the synthesized precession images for crystals of **PrHOTP** and **SmHOTP**.

### Correlated Disorder in LnHOTP

Local correlations of the Ln disorder in the *ab*-plane give rise to an intricate structure that appears in the diffuse scattering. This information provides incisive clues to assigning the appropriate space group, particularly as pseudosymmetry and twinning are prevalent throughout the **LnHOTP** series. Determination of the appropriate space group is pivotal when solving the structures of the modulated crystals.

All of the observed diffuse scattering occurs only in the odd-*l* planes, where the scattering preserves the systematic absences corresponding to the Ln···Ln distance (∼3 Å). Although batch-to-batch variations give rise to different types of diffuse scattering, there appears to be no significant correlation between the identity of the lanthanide and the type of observed diffuse scattering. Most patterns either form a tiling of triangles (Figure 2d) or areas of scattered intensity between Bragg peaks (Figure 2e).

To understand the origin of the diffuse scattering, we utilized the forward Monte Carlo method.^9–13^ This approach simulates a large supercell, where the occupation of each Ln site is influenced by its interaction with neighboring sites. As the Ln atoms are each situated in two possible sites, this simulation can be mapped to an Ising model of stacked Kagome lattices (Figure 2a). The Hamiltonian for such a model displays the form, *H* = *J*_*a*_ ∑_*i,j*|*a*_ *S*_*i*_ *S*_*j*_ + *J*_*b*_ ∑_*i*,*j*|*b*_ S_i_ *S*_*j*_ + ···, where each *J* coupling term defines a type of pairwise neighboring interaction (Figure 2b). For example, *J*_*XY*_ defines nearest-neighbor interactions of a disordered Ln pair with its four nearest neighbors in the *ab*-plane, while *J*_*Z*_ defines the nearest-neighbor interaction of a disordered Ln pair with its two nearest neighbors along the *c*-axis. Some of the observed patterns require the introduction of more-complex cross-pore interactions, *J*_*K*_ and *J*_*D*_, illustrated in Figure 2b. After generating the supercell from the Monte Carlo method, the diffuse scattering was calculated using the DISCUS software.^14^

In all crystals, we find that the value of *J*_*Z*_ must be positive and large in order to reproduce the experimental data, where diffuse scattering is present only in the odd-*l* planes. A positive *J*_*Z*_ can be understood as a strong tendency for alternate stacking of the Ln pairs along the *c*-axis. Yet, the presence of *ab*-plane interactions adds complexity to the picture, for we observe not one, but three distinct scenarios (i.e., *J*_*XY*_ = 0, *J*_*XY*_ > 0, and *J*_*XY*_ < 0). In the simplest case, when all pairwise interactions in *ab* plane vanish (i.e., *J*_*XY*_ = 0), one arrives at the average structure *P*6/*mcc* (Figure 2c).

For situations where *J*_*XY*_ > 0 (analogous to ferromagnetic interactions on a magnetic Kagome Ising lattice), the simulated diffuse scattering reproduces the experimentally observed diffuse scattering of a tiling of triangles with the majority of the diffuse scattering centered near the Bragg peaks (Figure 2d). Inspection of the simulated supercell reveals the presence of two domains, both of which obey local *P* 3 *c*1 symmetry. These domains are related by an *ab*-mirror plane, [1 0 0, 0 1 0, 0 0 –1]. Addition of this symmetry element, brings *P* 3 *c*1 (order 12) to the *P*6/*mcc* supergroup (order 24), which is consistent with a descent in symmetry from the *P*6/*mcc* average structure. Interestingly, refinement of the diffraction data with triangular diffuse scattering requires the addition of a twin law (the *ab*-mirror plane), suggesting that there are macroscopically large domains crystallizing in *P* 3 *c*1 that contain small domains of its twin. We have found crystals that solve in *P* 3 *c*1 (with a twin law) that do not display any significant amount of diffuse scattering, which indicates that it is possible to achieve long-range order with *J*_*XY*_ ≫ 0.

Simulations with *J*_*XY*_ < 0 require additional cross-pore interactions (denoted as either *J*_*K*_ or *J*_*D*_) to reproduce the experimental data in Figure 2e. Simulations with *J*_*K*_ > 0 produce lines of diffuse scattering that connect Bragg peaks, whereas simulations with *J*_*D*_ < 0 produce hexagonal regions of diffuse scattering with vertices at the Bragg peaks. We have yet to identify crystals with lines of diffuse scattering connecting Bragg peaks; however, many crystals display a single diffuse peak between Bragg peaks, which can be interpreted as an ordered limit of the *J*_*K*_ > 0 case. In both *J*_*K*_ and *J*_*D*_ cases, inspection of the simulated supercell evidences a local domain that can be described in *C*2/*c* (or the related *P*2_1_/*n*, similar to reported structures of **LaHOTP** and **NdHOTP**).^5^ When described as *C*2/*c*, there are 6 possible domains, which are related by an *ab*-mirror plane and a 3-fold rotation about *c*. These symmetry elements raise the order of the group from 8 (derived from the 2-fold rotation axis, the *c*-glide, and the centering) to 24 (removing a factor of 2 as the cell size is halved). Refinement of diffraction data for crystals obeying either *C*2/*c* or *P*2_1_/*n* require application of twinning with 6 possible domains, again emphasizing the relationship between microscopic and macroscopic domains. Notably, we have yet to observe diffuse scattering corresponding to *J*_*XY*_ < 0 but with no other cross-pore interactions (*J*_*K*_ and *J*_*D*_). If such a case existed, it would be analogous to a spin-frustrated antiferromagnetic Kagome Ising lattice (). We note that, in addition to the *P* 3 *c*1 and *C*2/*c* structures, we also observed diffuse scattering consistent with a cell tripling, whose modeling is not well described by pairwise interactions relevant to our treatment here ().

We have yet to establish a clear relationship between empirical trends and the type of local ordering in **LnHOTP**. However, given that the majority of crystals within a batch display the same type of diffuse scattering, the crystallization condition clearly plays a significant role in determining the local ordering. Slight changes in the oxidation state, nucleation and growth kinetics, and the tendency to twin likely influence the modulation.

### Polymorphism in SmHOTP

Samarium, the smallest lanthanide in our series, deserves special attention because it hosts a second polymorph. Within a single batch of **SmHOTP**, there are two types of crystals: one consistent with *C*2/*c* (similar to the previously discussed structures) and a new *P*2_1_/*n* phase, where the SBUs are best described as discrete Sm_2_ dinuclear units (with Sm···Sm contacts of ∼4 Å). This phase can also be regarded as a descent in symmetry from the average structure in *P*6/*mcc*, with loss of the 3-fold rotation axis and the *ab*-mirror plane (similar to the *C*2/*c* structures), with additional descent in symmetry caused by shifts of the position of the Sm atom along the (formerly defined) *c*-axis. Here, the Sm atoms remain 8-coordinate, to five oxygen atoms from HOTP ligands and three additional oxygen atoms. While the local Sm geometry in the *C*2/*c* phase can be described as a triangular dodecahedron (*D*_2*d*_), the local geometry in the *P*2_1_/*n* phase is best described as a bicapped trigonal prism (*C*_2*v*_) (see ). The π–π contacts between neighboring HOTP molecules in the *P*2_1_/*n* phase are slightly longer than the corresponding *C*2/*c* phase, 3.18 Å compared with 3.05 Å. Only the *C*2/*c* phase displays diffuse scattering. The crystals grow as non-merohedral twins with a 6-fold rotation about the formerly defined *c*-axis. The formation of local domains is likely prevented as the twins cannot be merohedral, given that the β angle is not close to 90°.

### Commensurability in LnHOTP

Previously, we observed satellite peaks in the diffractograms of **LaHOTP** and **NdHOTP** below a critical temperature of 361–365 K; these peaks were thought to originate from periodic modulations associated with the CDW instability.^5^ However, the structure at lower temperatures remained largely unexplored, limiting our interpretation on the nature of the CDW. Utilizing the diffuse scattering as a guide to determine the appropriate symmetry, we sought to determine the low-temperature modulated structures of **LnHOTP** (Ln = La, Ce, Pr, and Nd).

Rather unusually for CDW materials, we find that **LnHOTP** crystals prepared under the same protocol are capable of hosting a wide range of modulation wavevectors. Each **LnHOTP** (Ln = La, Ce, Pr, and Nd) crystal exhibits a single modulation along the *c*-axis with a wavevector ranging from *q* = 0.18*c* to *q* = 0.5*c* (Table 1). In all of the samples tested, we have only observed first-order satellite peaks from the diffraction data. The wavevector is batch-to-batch variant—likely depending on the oxidation state of the ligand—but is found to be consistent within a batch. We attribute the large variability in the wavevector to (1) the redox-noninnocent nature of the HOTP ligand and (2) the pores of the MOF that can host either negatively or positively charged species. Importantly, some of the modulation vectors are commensurate (i.e., wavevectors with simple rational coordinates) whereas others are incommensurate (i.e., wavevectors of noninteger fractions). All crystals that displayed triangular diffuse scattering could be solved in superspace, resulting in a superspace group assignment of *P* 3 *c*1­(00γ)­0*s*0. Experimentally, the modulation satellites are strongest in the even-*l* planes, but are also present in the odd-*l* planes. Additionally, there is no strong diffuse scattering in the *hk*-planes for the satellites, indicating that the modulation and the diffuse scattering are unrelated. Although satellite peaks are found in all of the **LnHOTP** crystals of the early lanthanides, samarium presents a unique case where none of the tested crystals display modulation. In *C*2/*c*-**SmHOTP**, we have not observed any periodic modulations at 298 K. As for *P*2_1_/*n*-**SmHOTP**, we have observed one batch exhibiting a cell-doubling along the formerly defined *c*-axis, yet the dataset was not of sufficient quality for structure solution.

**Table 1:** Structural Parameters for Refinements

Ln	Temperature (K)	Long lattice parameters (Å)	Short lattice parameter (Å)	*q* (*c*)	Space group
La	100	*a* = 22.1276(9)	*c* = 6.0552(3)	^1^/_4_	*P* 3 *c*1(00γ)0*s*0, (*t*_zero_ = 0.0625, *P*321 supercell)
La	298	*a* = 22.1205(7)	*c* = 18.3698(6)	^1^/_3_ ^a^	*P* 3 *c*1
Ce	100	*a* = 22.0584(10)	*c* = 6.0609(2)	0.240870	*P* 3 *c*1(00γ)0*s*0
Pr	100	*a* = 22.0438(5)	*c* = 6.02540(10)	^1^/_4_	*P* 3 *c*1(00γ)0*s*0, (*t*_zero_ = 0.0625, *P*321 supercell)
Nd	100	*a* = 21.9472(8)	*c* = 12.1112(7)	^1^/_2_ ^a^	*P*321
Sm	298	*a* = 21.9654(12)	*c* = 6.09294(16)	Not observed	*C*2/*c*
		*b* = 38.0331(13)			
		β = 89.934(4)			
Sm	100	*c* = 21.877(2)	*a* = 6.3682(6)	Not observed	*P*2_1_/*n*
		*b* = 38.704(5)			
		β = 95.771(7)			

a Refined in a supercell rather than superspace.

Commensurate modulation belongs to a special class of superlattices, in which the wavevector coordinates in reciprocal lattices are simple rational fractions. In **LnHOTP**, we observe three distinct cases of commensurate modulation, corresponding to wavevectors of ^1^/_2_ *c*, ^1^/_3_ *c*, and ^1^/_4_ *c*. In the commensurately modulated structures, there are various options for the origin in the internal dimension, *t*_0_ (also referred to as the phase of the modulation), which affects the symmetry of the supercell (Figure 3). For *q* = ^1^/_4_ *c*, there are two nongeneral options for space group assignment: *P* 3 and *P*321. We find that refinement in *P*321 results in a better fit as characterized by lower *R*-value in all cases (see Tables 2, 3, and 4). For *q* = ^1^/_3_ *c*, there is only one nongeneral option, *P* 3 *c*1. For *q* = ^1^/_2_ *c*, there are two nongeneral options: *P* 3 and *P*321. Again, refinement in *P*321 results in the superior model. For the *q* = ^1^/_2_ *c* and *q* = ^1^/_3_ *c* cases, we chose to refine directly in real space (i.e., using a supercell), whereas for the *q* = ^1^/_4_ *c* case, we refined using the superspace formalism—because there are only first-order satellites, 25% of the expected Bragg peaks for a supercell are systematically absent.

**Figure 3:**
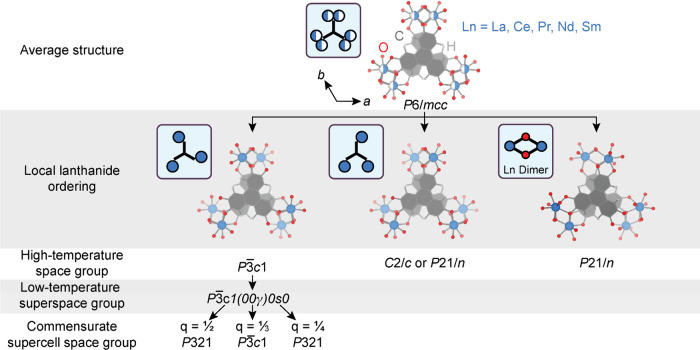
Group-subgroup relations for **LnHOTP**.

**Table 2:** Comparison of Refinement of **LaHOTP** Supercell in ***P* 3** and *P*321

**LaHOTP**	*P* 3, *R*(all)	*P* 3, *wR*2(all)	*P*321, *R*(all)	*P*321, *wR*2(all)
All reflections	9.22	18.58	8.72	17.95
Main reflections	6.92	16.14	6.88	15.95
Satellites	11.92	20.92	10.88	19.92

**Table 3:** Comparison of Refinement of **PrHOTP** Supercell in ***P* 3** and *P*321

**PrHOTP**	*P* 3, *R*(all)	*P* 3, *wR*2(all)	*P*321, *R*(all)	*P*321, *wR*2(all)
All reflections	15.20	20.96	14.86	20.75
Main reflections	11.24	22.12	11.20	22.07
Satellites	20.36	18.62	19.64	18.06

**Table 4:** Comparison of Refinement of **NdHOTP** Supercell in ***P* 3** and *P*321

**NdHOTP**	*P* 3, R1	*P* 3, wR2	*P*321, R1	*P*321, wR2
All reflections	10.13	27.26	8.46	24.70

The effect of the modulation is a continuous displacement of all atoms, which causes rotation of the HOTP ligands in the *ab*-plane and a slight displacement of the Ln atoms (Figure 4a, b). We hypothesize that by increasing the relative rotation, δθ, of one HOTP unit relative to its neighbors, all ligands can carry more charge (i.e., increasing charge density on the ligands does not cause as much repulsion when the ligands are displaced relative to each other). Likewise, a smaller value of δθ is associated with decreased charge density on the ligands and a redistribution of charge density across the MOF, resolving the CDW instability. The effect of the modulation on the Ln atoms is less pronounced. In all cases, the local Ln geometry is best described as a distorted dodecahedron (with lowest continuous symmetry measures, ranging from 2 to 3),^15,16^ implying that the modulation does not significantly influence the coordination type of the lanthanide (see ).

**Figure 4:**
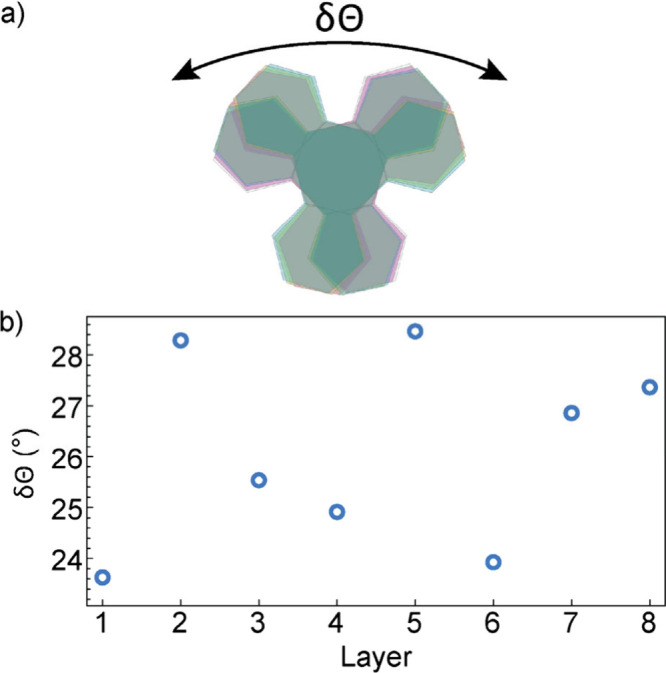
(a) The structure **PrHOTP**, highlighting the changes to the relative rotation of neighboring HOTP. (b) The relative rotation of neighboring HOTP for **LaHOTP** defined by the dihedral angle Φ­(O–C–C–O) between neighboring HOTP molecules.

It is intriguing that only a single modulation is observed for **LnHOTP** given that two bands cross the Fermi level.^5^ These two bands are nested, such that both run from Γ to A in the Brillouin zone. Utilizing simple Hückel-type arguments (Figure 5b, ), these two bands must originate from the doubly degenerate HOMO/HOMO–1 and the nondegenerate HOMO–2 orbitals of the fully reduced HOTP^6–^ molecule. Given that the oxidation state of the ligand in **LnHOTP** is nominally HOTP^3–^, the Fermi level would, in theory, cut through both bands.

**Figure 5:**
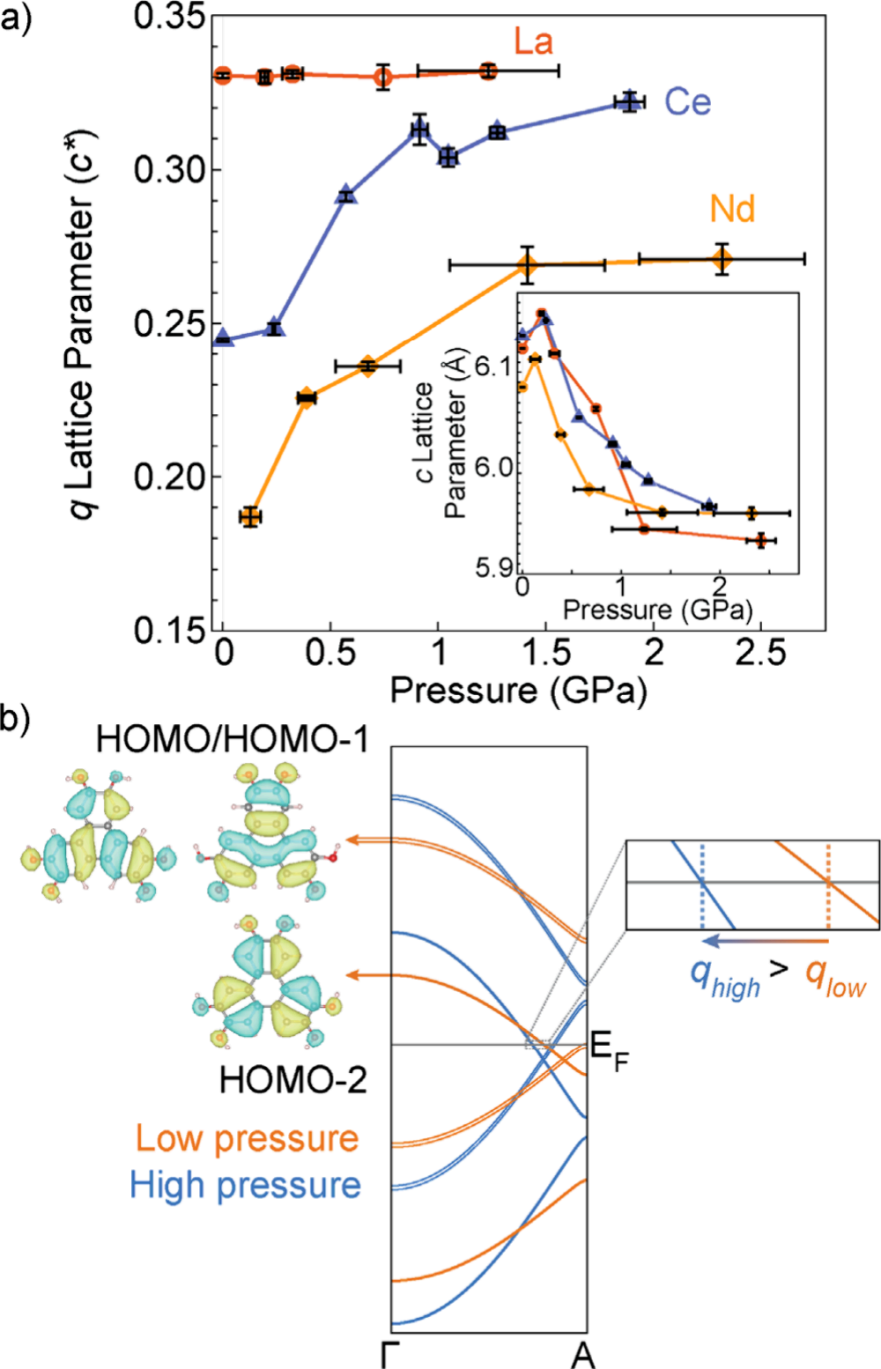
(a) The pressure dependence of the modulation wavevectors and *c* lattice parameters for **LaHOTP**, **CeHOTP**, and **NdHOTP**. (b) A qualitative Hückel-type band structure derived from the frontier orbitals of the HOTP^6–^ molecule.^5,25^ The thin lines are derived from the HOMO–2 orbital and the thick lines are derived from the HOMO/HOMO–1 degenerate set. This band structure is consistent with the band structure previously calculated from DFT. As the pressure increases, there is a cell contraction that increases orbital overlap, which increases band dispersion and shifts the band crossing closer to *q* = 0.5 *c*.

We present three potential scenarios that are in agreement with our observation of a single modulation from 100 K to 365 K.^1–3^ (1) There exists a lower critical temperature at which a second modulation appears. (2) There exists a lower critical temperature at which the first modulation becomes temperature dependent. (3) A lower critical temperature is nonexistent. The observed transition at 365 K is a metal–metal transition consistent with the picture of a single gapped band. If a secondary modulation were to appear at lower temperatures, we may expect a metal–insulator phase transition in which both bands would be gapped, but this is not observed above 100 K.

### Pressure-Induced Ordering in LnHOTP

It is often the case that CDWs are pressure-dependent; application of pressure can cause variation in the modulation vector or suppress the CDW phase.^4,17^ To this end, we sought to analyze the pressure response of **LnHOTP** in a diamond anvil cell (DAC). When performing variable-pressure experiments on MOFs, it is crucial to select a suitable pressure-transmitting medium that would not enter the pores. **LnHOTP** crystals were thus immersed in polyphenyl-methylsiloxane (PPMS) to ensure hydrostatic conditions.^18–20^ Preliminary variable-pressure PXRD measurements based on a prototype cell indicate that **LnHOTP** is stable up to at least 0.68 GPa with minimal loss in crystallinity (). From the data collected at each pressure, the unit-cell parameters could be fitted with TOPASv6. In all cases, we report on a reduction in both the *a* and *c* lattice parameters upon pressurization.^21^ Fitting this data using PASCal,^22^ the bulk moduli were calculated from second- and third-order Birch–Murnaghan Coefficients as B_0_ = 8.1 ± 4.3 GPa for **CeHOTP**, 9.9 ± 3.4 GPa for **PrHOTP**, and 11.9 ± 3.2 GPa for **NdHOTP.** Due to the weak intensity of the modulation satellites relative to main reflections, the modulation wavevector could not be determined simply from PXRD.

For this purpose, variable-pressure single crystal X-ray diffraction was performed on both commensurately and incommensurately modulated samples of **LnHOTP** (Ln = La, Ce, and Nd) at a pressure range of 0–2.5 GPa (Figure 5a). The diffuse scattering of **LaHOTP** (commensurate), **CeHOTP** (incommensurate), and **NdHOTP** (incommensurate) were in accordance with *P* 3 *c*1, *C*2/*c*, and a cell tripling, respectively. For all three samples, the lattice parameters were extracted using the average trigonal cell. Above 2.5 GPa, we observed a noticeable decline in crystal quality, which precluded measurements at higher pressures. Upon pressurization, the wavevectors of **NdHOTP** and **CeHOTP** increased from 0.19*c* to 0.27*c* and 0.24*c* to 0.32*c*, respectively. An increase in the wavevector is a consequence of unit-cell contraction and a subsequent improvement of orbital overlap; this effectively increases band dispersion and shifts the Fermi-level-crossing closer to *q* = 0.5 *c* (Figure 5b). For **LaHOTP**, the wavevector remains commensurate at ^1^/_3_ *c* at all pressures. Importantly, such a locking-in of commensurability is in agreement with an energetic stabilization for classic CDW systems.^4,23,24^

## Conclusion

Charge-density-wave (CDW) order in materials with intrinsic structural porosity presents an unresolved structural and electronic problem. Here, we report a systematic investigation of **LnHOTP** (Ln = La, Ce, Pr, Nd, and Sm), a CDW material in which porosity is an inherent feature of the framework. Analysis of the crystal structure establishes the origin of the diffuse scattering, the modulated structure, and the pressure dependence of the modulation. The modulation corresponds to a continuous displacement of all atoms accompanied by a rotation of the HOTP ligands transverse to the stacking axis, providing insight into the physical origin of the CDW state. The modulation wavevector increases monotonically with pressure, consistent with a simple Hückel model in which pressurization enhances orbital overlap between neighboring HOTP units. At higher pressures, a commensurate lock-in at *q* = ^1^/_3_ *c* is observed, indicating energetic stabilization of the modulated phase.

We anticipate that these findings will shed useful light on the structure–function relationships and the novel electronic states yet to be discovered in 2D conductive MOFs. Continued forays into the low-temperature regime may reveal additional anomalies, such as secondary order–disorder transitions and nonlinear phenomena.

## Supplementary Material





















## Notes

General information, experimental information, elemental analysis, crystallographic details, code for simulating diffuse scattering (PDF)
t-plot movie #1 of incommensurate CeHOTP (.gif)
t-plot movie #2 of incommensurate CeHOTP (.gif)
Crystal structure of LaHOTP (P-3c1­(00g)­0s0) (CIF)
Crystal structure of SmHOTP (C2/c) (CIF)
Crystal structure of CeHOTP (P-3c1­(00g)­0s0) (CIF)
Crystal structure of NdHOTP (P321) (CIF)
Crystal structure of SmHOTP (P21/n) (CIF)
Crystal structure of PrHOTP (P-3c1­(00g)­0s0) (CIF)
Crystal structure of LaHOTP (P-3c1) (CIF)
