# Supplementary material for: Evidence for an Electronically Driven Charge Density Wave in a 1D Metallic MOF
Source: ACS Cent Sci. 2026 May 5;12(5):704–11. doi: 10.1021/acscentsci.6c00405 (PMC13220207; doi:10.1021/acscentsci.6c00405)
Supplement: Supplementary file 1 [file oc6c00405_si_001.pdf]

Supplementary Information

Evidence for an electronically-driven charge density wave in a 1D metallic MOF

Jewel Ryu[a,b], Lukas Sippach[c,d], Sebastian A. Hallweger[c], Lukáš Palatinus[e], Peter Müller[a], Konstantin Glazyrin[f], Gregor Kieslich[c,d], Julius J. Oppenheim[b*], Mircea Dincă[b*]

[a] Department of Chemistry, Massachusetts Institute of Technology, 77 Massachusetts Avenue, Cambridge, Massachusetts, USA
[b] Department of Chemistry, Princeton University, Washington Road, Princeton, New Jersey, 08540, United States
[c] Technical University of Munich, TUM School of Natural Sciences, 85748, Lichtenbergstraße 4, 85748 Garching, Germany
[d] Institute of Inorganic and Analytical Chemistry, Justus Liebig University Giessen, Heinrich-Buff-Ring 17, 35392, Giessen, GER.
[e] Institute of Physics of the Czech Academy of Sciences, Na Slovance 1999/2, 18200 Prague, Czechia
[f] Deutsches Elektronen-Synchrotron DESY, Notkestr. 85, 22607 Hamburg

Section .... Page
1. General information 2
2. Synthetic methods 4
3. Elemental analysis 9
4. Powder X-ray diffraction 10
5. Crystallographic details 11
6. Simulation of diffuse scattering 17
7. Continuous shape and symmetry measures 27
8. Variable pressure diffraction 36
9. Construction of Hückel band structure 50
10. References 54

Section S1. General Information

Materials. $La(NO_3)_3{\cdot}6H_2O$ (Sigma Aldrich, 99.9%), $Ce(NO_3)_3{\cdot}6H_2O$ (Sigma Aldrich, 99.9%), $Pr(NO_3)_3{\cdot}6H_2O$ (Sigma Aldrich, 99.9%), $Nd(NO_3)_3{\cdot}6H_2O$ (Sigma Aldrich, 99.9%), $Sm(NO_3)_3{\cdot}6H_2O$ (Sigma Aldrich, 99.9%), *N*,*N*-dimethylacetamide (DMA; Sigma Aldrich, 99% zerO_2), boron tribromide (BBr_3; Sigma Aldrich, ≥99.9%), and 2,3,6,7,10,11-hexamethoxytriphenylene (HMTP; AmBeed, 97%) were used as received. Dichloromethane (DCM; Sigma Aldrich, for HPLC, ≥99.9%) was dried and deoxygenated using a Glass Contour Solvent Purification System. Deionized water and methanol (MeOH; Fisher, low water, ≥99.8%) were deoxygenated by bubbling with N_2 under the Schlenk line for 24 hours. Red silicone O-rings (Ace Glass 7855-218, I.D. 10.2 mm, wall 2.6 mm) and 15 mL-capacity glass pressure tubes (Synthware) were prepared for MOF synthesis.

MOF synthesis, reaction work-up, and solvent washes were performed in an Innovative Technology glovebox with a N_2 atmosphere. Measurements after the washes were performed in air, and the samples were stored in aerated solvents without loss of crystallinity for more than 6 months.

In house powder X-ray diffraction. A Bruker Advance II diffractometer with $\theta/2\theta$ reflection geometry and Ni-filtered Cu Kα radiation (Kα_1 = 1.5406 Å, Kα_2 = 1.5444 Å, Kα_2/Kα_1 = 0.5) was used to record PXRD patterns. The tube voltage and current were set to 40 kV and 40 mA, respectively. Thin layers of samples were placed on a zero-background silicon crystal plate for PXRD measurements.

In-house single-crystal X-ray diffraction. Low-temperature diffraction data were collected on a Bruker-AXS X8 Kappa Duo diffractometer with IμS micro-sources using either Mo Kα radiation (λ = 0.71073 Å) or Cu Kα (λ = 1.54178 Å), performing ϕ-and ω-scans. The diffractometer was equipped with a Smart APEX2 CCD detector.

LaHOTP (q = 1/3 c) and **NdHOTP** (q = 1/2 c) were solved by dual-space methods using SHELXT[1] and refined against F^2 on all data by full-matrix least squares with SHELXL2017[2] following established refinement strategies.[3] All hydrogen atoms were included into the model at geometrically calculated positions and refined using a riding model. The isotropic displacement parameters of all hydrogen atoms were fixed to 1.2 times the U-value of the atoms they are linked to. Details of the data quality and a summary of the residual values of the refinement are listed in **Tables S1-7**.

Modulated structures were imported into Jana2020,[4] and the crystal structures were solved in superspace using Superflip.[5,6] The structures were refined using the superspace formalism.

Synchrotron high-pressure single crystal X-ray diffraction. High-pressure single-crystal X-ray diffraction (HP-SCXRD) experiments were performed at beamline P02.2 of the PETRA

III synchrotron facility (Deutsches Elektronen-Synchrotron, DESY, Hamburg) using an X-ray energy of 42.7 keV (λ = 0.29 Å) and a PerkinElmer XRD1621 2D area detector.

Single crystals (~120 × 10 × 10 μm^3) were selected and loaded into a diamond anvil cell (DAC) with a 90° opening angle, equipped with Boehler–Almax diamonds (culet size: 800 µm). The large culet size allowed for fine pressure increments and improved pressure resolution. A stainless-steel gasket was used as the gasket material and silicone oil AP-100 as the pressure-transmitting medium. A ruby chip was added as a pressure calibrant. A small, spherical tungsten particle (d < 10 µm) was included for the alignment of the sample with respect to the X-ray beam and for the centering of the DAC with respect to the goniometer rotation axis (strong X-ray absorber).
Diffraction images were collected at each pressure point by rotating the DAC from −30° to +30° in ω with 1° steps. Measurements were performed from ambient pressure up to 2.5 GPa.

Elemental analyses were carried out by Robertson Microlit Laboratories in Ledgewood, New Jersey. Measurements were performed in duplicate.

Diffuse scattering were simulated using the DISCUS suite (version 6.17.02).[7]

Continuous symmetry and shape measurements were performed on commensurate **LnHOTP** crystals using the SHAPE 2.1 software.[8,9] We selected two distinct polymorphs of commensurately modulated **LnHOTP** crystals with different q vectors: **LaHOTP** (q = 1/3 c) and **NdHOTP** (q = 1/2 c). Analyses were performed on each of the distinct eight-coordinate La and Nd coordinates appearing in the crystal structure, including both major and minor occupancy sites (i.e., disordered positions). The unit cell for commensurate crystals typically contains 4 to 8 distinct lanthanide positions, including both main and disordered sites. Formally seven-coordinate or four-coordinate Ln sites could not be assigned an eight-coordinate polyhedra, and were excluded from the analysis.

Nuclear magnetic resonance (NMR) experiments were performed on a three-channel Bruker Avance Neo spectrometer (500 MHz). ^{1}H-NMR spectra are internally referenced to residual solvent signal at δ = 2.50 (DMSO-d_6).

Section S2. Synthetic methods

Synthesis of H_6HOTP (2,3,6,7,10,11-hexahydroxytriphenylene)

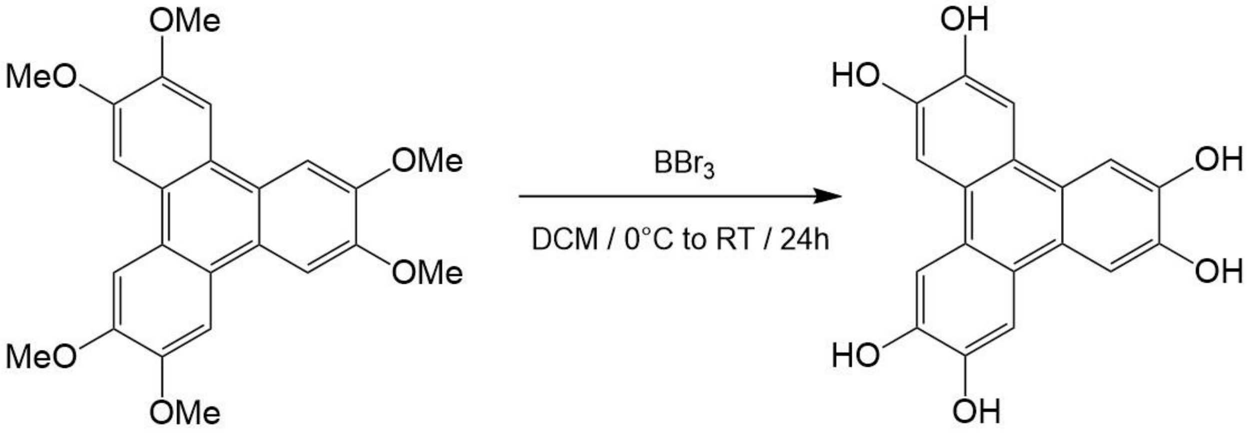

Figure S1. Reaction scheme for the synthesis of H_6HOTP. Deprotection of the methoxy groups was achieved by adding BBr_3 in dry DCM to HMTP at 0°C under high N_2.

In a 500 mL Schlenk flask, 2.04 g of HMTP (1 eq., 4.99 mmol) (hexamethoxytriphenylene) was stirred in 80 mL dry DCM using a magnetic stir bar for 1 h (20.0 °C, 500 rpm). The flask was then placed in a wide, flat-bottomed beaker filled with ice. 60 mL of 1 M BBr_3 (12 eq., 60 mmol) in dry DCM was added over 10 minutes through a syringe at 0 °C in a high-N_2 environment under the Schlenk line. The suspension cleared up after 10 mL, leading to the formation of a light yellow-white precipitate. The suspension was allowed to warm up to room temperature and was stirred for 24 h (20.0 °C, 550 rpm) to produce a light tan-cream suspension.

150 mL of cold deoxygenated water was added to the suspension under high N_2 at the Schlenk line, either using cannula transfer or through multiple injections of 10 mL syringes. The suspension was stirred until gases were no longer produced. Upon the addition of water, the suspension transformed into a creamy, off-white mixture. In this step, it is critical to ensure that the water is fully deoxygenated, and that all processes are kept strictly air-free. Trace amounts of impurities or oxygen in the cannula or syringe tips may result in ligands with a pale blue coloration.

The suspension was vacuum-filtered and washed with deoxygenated water in a N_2-filled purge box for 1 hour. The powder was transferred into an amber glass vial to account for the slight light sensitivity of H_6HOTP. The vial was taken to the Schlenk line, where the powder was dried overnight under vacuum. The following day, the collected powder of H_6HOTP was stored in the fridge of an MBraun glove box (yield 85%).

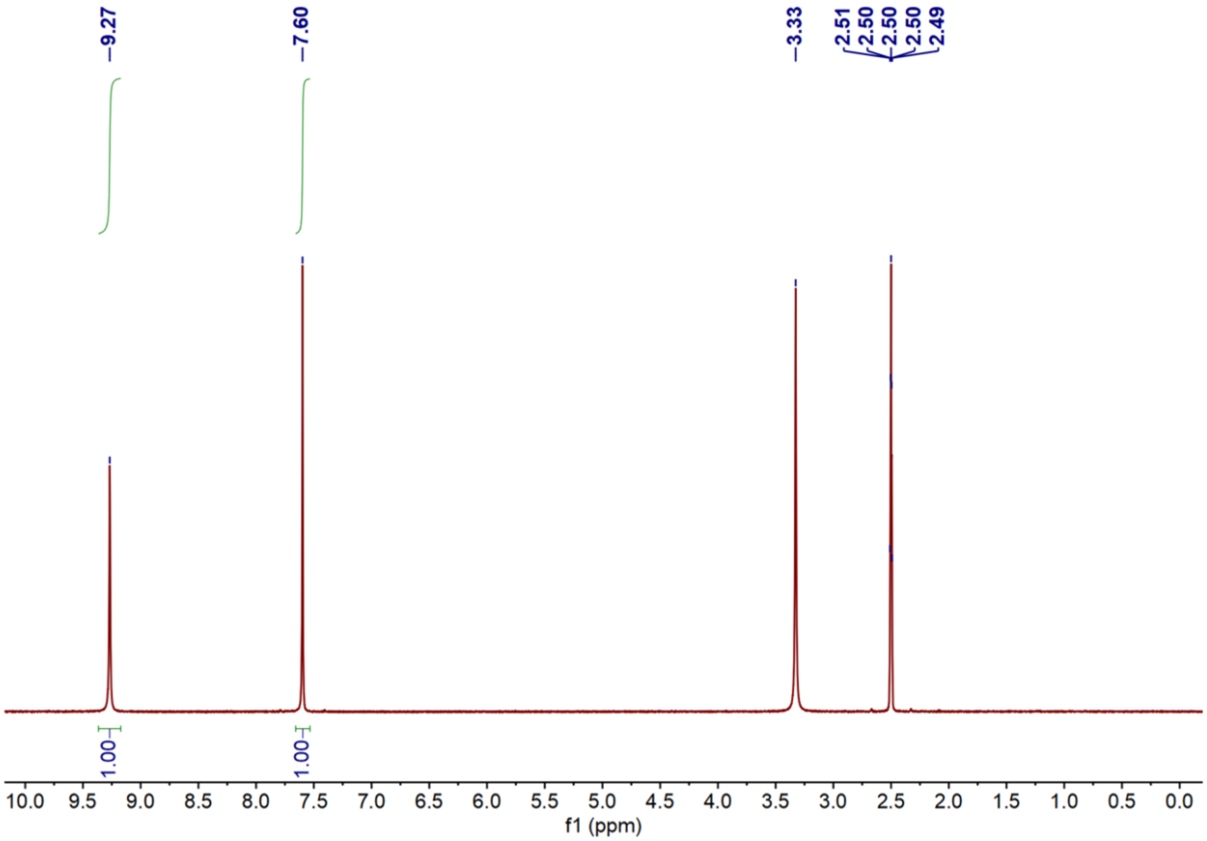

Figure S2. Nuclear magnetic resonance (NMR) spectrum of synthesized H_6HOTP, measured in dimethylsulfoxide-d6 on a Bruker Advance-III HD Nanobay spectrometer operating at 400.09 MHz.

Completely unoxidized and purified H_6HOTP powders exhibit an off-white coloration. Impurities or incomplete washing of the ligand can lead to a yellowish hue. Slightly oxidized H_6HOTP tends to take on a sky-blue hue. Prolonged oxidation, especially in the presence of water or solvents, will lead to a light pink shade. Further oxidation will lead to a dark blue, indigo, or purple hue. Complete oxidation or a high concentration of impurities will yield a dark gray or black powder. We find that it is important to strive towards an off-white color of the ligand to obtain high-quality single crystals of **LnHOTP**, as H_6HOTP is sensitive to air and water. Slightly pink or purple H_6HOTP powders were unable to produce single crystals of **LnHOTP** suitable for diffraction. While yellow or more darkly saturated hues of ligand solutions are capable of producing powder forms of **LnHOTP** or other variants of **MHOTP** (M = transition metal) MOFs, it is challenging to achieve high-quality crystals under these conditions.

Recrystallization of H_6HOTP

The laboratory was equipped with a special N_2-filled glove box containing water, designated for air-sensitive reactions requiring aqueous solutions. In this glove box, 20 to

30 mg of H_6HOTP powder was measured in multiple 20 mL scintillation vials, to which 2 mL DMA was subsequently dispensed with a pipette. The H_6HOTP ligand was heated up at 100 °C in 2 mL DMA, then let to recrystallize overnight. This led to the formation of transparent needle-shaped crystals of the H_6HOTP ligand. Crystals had formed in approximately 50% of the scintillation vials containing supersaturated solutions of the H_6HOTP linker, while recrystallization was not observed in the remaining vials. Yet by adding a small amount (1-5 mg) of ligand seed crystals in the unsuccessful supersaturated solutions, we observed the rapid formation of numerous crystals, which could subsequently be salvaged. From each vial, DMA was dispensed with a pipette so that the H_6HOTP crystals on the bottom could be collected.

Note that the conditions for obtaining high-quality crystals may be dependent on the ambient environment or recrystallization conditions. For instance, recrystallization did not happen as readily in the MBraun glove box as in the N_2-filled glove box containing water vapor. Adding slightly larger volumes of DMA in the scintillation vials was also unfavorable in terms of achieving a fully supersaturated solution. Recrystallization was observed for white, light yellow, or sky-blue powders of H_6HOTP, which equally yielded transparent H_6HOTP crystals. However, recrystallization was generally unsuccessful for pink/purple or more deeply saturated colors of H_6HOTP.

Synthesis of LnHOTP (Ln = La, Ce, Pr, Nd, and Sm)

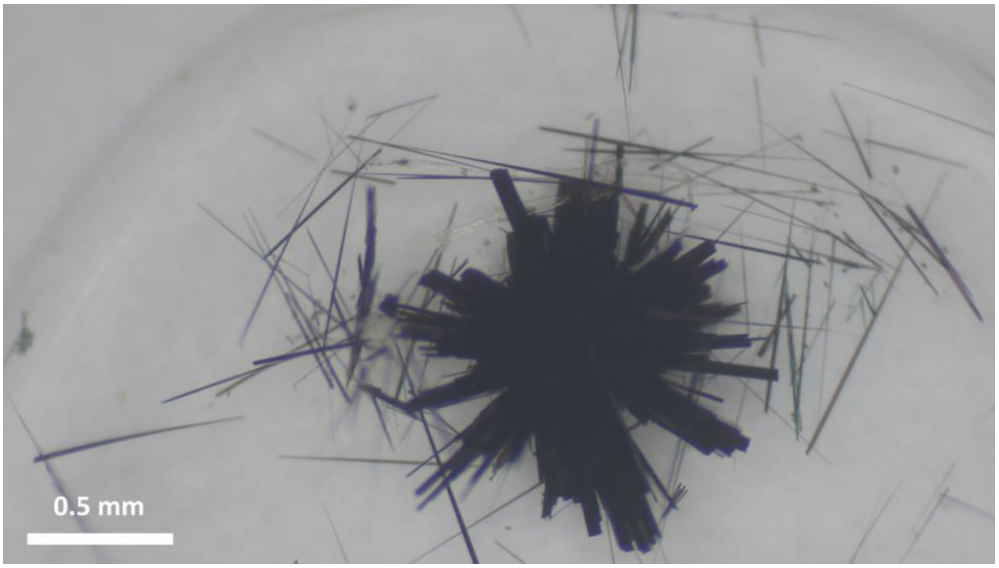

Figure S3. Optical microscopy image of **LnHOTP** single crystals and clusters, shown for PrHOTP (scale bar: 0.5 mm). Crystals of the other lanthanides display identical or similar morphologies.

In separate 20 mL scintillation vials, 9.6 mmol (160 equiv.) of $Ln(NO_3)_3{\cdot}6H_2O$ (Ln = La, Ce, Pr, Nd, and Sm) was each measured. 6 mL of deoxygenated water was dispensed into each

vial. This was shown to be effective because a large excess of lanthanides keeps the acidity at the desired state. A high concentration of the metal salt can be a driving force for stabilizing the MOF at high temperatures.

In an N_2-filled glove box, 0.060 mmol of recrystallized H_6HOTP (1 equiv.) was measured in 20 mL scintillation vials, to which 0.7 mL DMA was subsequently dispensed with a pipette. The ten vials—one for each linker solution and one for each metal salt were placed on the hot plate, set to 90 °C. The metal salts easily dissolve in water to produce a clear solution. Upon heating to 90 °C on a hot plate, the transparent H_6HOTP crystals transformed into a clear supersaturated solution. Note that the color of the solution is paramount. If the ligand is dark blue/purple or black, it will dissolve rather quickly to form a darker bluish or blackish solution, and this will be quite evident from any angle. If there is a hint of yellow in the solution, it is a clear but indication that H_6HOTP had already started to oxidize or accumulate impurities.

After the solutions had fully dissolved, the vials were lowered from the hot plate. Using a micropipette, 0.4 mL of 0.1 M HCl was added to the metal salt solutions, which now added up to 6.4 mL. The concentration and volume of the acid modulator would play a key role in the buffering effect. The linker solutions were then combined with their respective metal salt solutions. Using 5 mL plastic syringes, the combined solutions were each passed through a 200 nm polyethersulfone filter and transferred into a 15 mL-capacity glass pressure tube. Note that this subtle alteration of the general protocol is not universal for MOFs; for *d*-block metals, care must be taken, as combining the solutions prior to filtration can rapidly lead to precipitates. In the case of lanthanides, combining the solutions before filtration was found to be less problematic.

Each pressure tube was fitted with a polytetrafluoroethylene screw plug with a red silicone O-ring. The pressure tubes were subsequently transferred out of the glove box and secured in an aluminum heating block. The heating block containing the pressure tubes was placed inside a preheated convection oven, which was set to 135°C. The mixture was kept at 135 °C for 72 h. After the reaction, the oven was cooled down to room temperature over 2 hours. Inspection of the contents of the pressure tubes indicated the formation of needle-shaped single crystals or star-shaped clusters of **LnHOTP**. In this step, proper sealing of the pressure tubes appeared to be crucial; pressure tubes fitted with black Teflon O-rings or slightly larger O-rings typically did not produce robust crystals.

After cooling, the pressure tubes were transferred into an N_2-filled glove box without further ado. The reaction was immediately worked up by removing the solution using a pipette and transferring the crystals into water. The mother liquor should be exchanged with deionized water as quickly as time permits, in order to prevent the formation of unfavorable organic crystals (e.g., acetates and formates) at room temperature. This was found to be especially important in the case of **CeHOTP**, likely due to the redox-active

nature of $Ce^{III/IV}$; gel-like phases or undesirable organic crystals may develop in the mother liquor within several hours at room temperature.

The collected crystals were washed several times with water to remove remaining salts within the MOF pores. Washing and decanting was repeated until the color of the solution became very faint. The water was decanted and exchanged with methanol, a lower-surface-tension liquid. The crystals were soaked and stored in methanol prior to X-ray diffraction. For additional physical measurements, the crystals were dried and activated by applying gentle heating (50 °C) under vacuum.

Section S3. Elemental analysis

Elemental analysis was performed by Robertson Microlit Laboratories, Ledgewood NJ. The **LnHOTP** crystals were activated at 90°C for 24 hours *in vacuo* on a Schlenk line and handled in N_2-filled gloveboxes. Carbon, hydrogen, and nitrogen contents were obtained using traditional CHN analysis. The content of the metal was obtained using inductively coupled plasma optical emission spectroscopy (ICP-OES).

$Ce_{1.5}$HOTP: $[Ce(NO_3)_{0.51}]_3(HOTP)_2 \cdot 28.0\ H_2O$

(calcd., found for $Ce_{1.5}C_{18}H_{34.0}N_{0.8}O_{22.3}$): C (27.41, 26.10), H (3.79, 2.41), N (1.360, 1.295), Ce (26.65, 27.01)

$Pr_{1.5}$HOTP: $[Pr(NO_3)_{0.35}]_3(HOTP)_2 \cdot 22.5\ H_2O$

(calcd., found for $Pr_{1.5}C_{18}H_{28.5}N_{0.5}O_{18.8}$): C (29.96, 28.25), H (3.32, 2.40), N (1.02, 0.96), Pr (29.29, 31.77)

$Sm_{1.5}$HOTP: $[Sm(NO_3)_{0.61}]_3(HOTP)_2 \cdot 22.4\ H_2O$

(calcd., found for $Sm_{1.5}C_{18}H_{28.4}N_{0.9}O_{19.9}$): C (28.23, 26.93), H (3.21, 2.40), N (1.68, 1.61), Sm (29.45, 28.33)

Additional solvents in the formula originate from the washing procedure, which involves the usage of water and methanol. Acetic acid (CH_3COOH) appears in the formula as the product of DMA hydrolysis. For the elemental analysis of $La_{1.5}$HOTP and $Nd_{1.5}$HOTP, refer to our previous report.[10]

Section S4. Powder X-ray diffraction

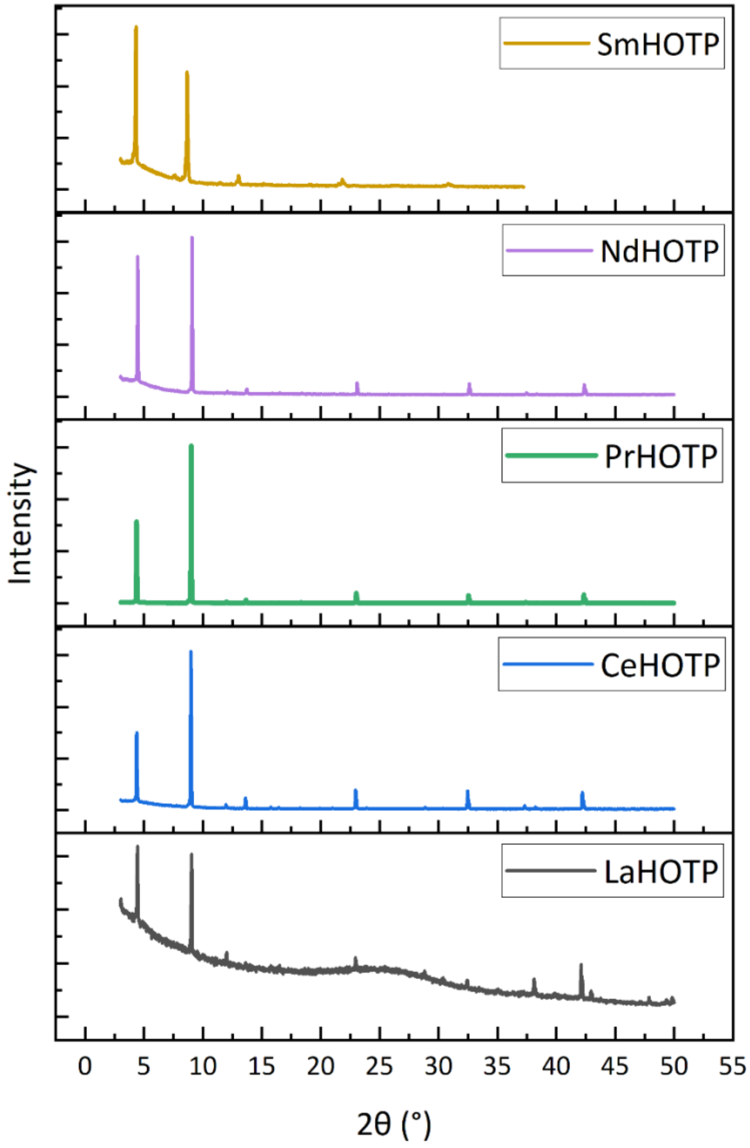

Figure S4. Powder X-ray diffraction patterns of **LnHOTP** (Ln = La, Ce, Pr, Nd, and Sm).

Section S5. Crystallographic details

Table S1. Crystallographic information for **LaHOTP** (q = 1/4 c).

Identification code (CCDC Deposition)	2500707
Empirical formula	$La_{1.5}C_{18}H_6O_{12}$
Formula weight	622.5
Temperature (K)	100
Crystal system	Trigonal
Space group	$P\bar{3}c1(00\gamma)0s0$
Commensurate t_0	0.0625
Supercell space group	$P321$
a (Å)	22.1276(9)
b (Å)	22.1276(9)
c (Å)	6.0552(3)
α (°)	90
β (°)	90
γ (°)	120
Modulation vector	(0, 0, 0.25)
Volume (Å^3)	2567.60(19)
Z	4
ρ_{calc} (g/cm^3)	1.6106
μ (1/mm)	19.586
Crystal size (mm^3)	0.440 x 0.070 x 0.070
Radiation	CuKα (λ = 1.54178)
2Θ range for data collection (°)	2.3056 to 74.6031
Index ranges	$-27 \le h \le 27$, $-27 \le k \le 27$, $-6 \le l \le 7$, $-1 \le m \le 1$
Reflections collected	53839
Independent reflections	5266
Data/restraints/parameters/constraints	5266 / 0 / 175 / 20
Goodness-of-fit on F^2	3.9087
Final R indexes [I>= 2σ(I)]	$R1$ = 0.0766, $wR2$ = 0.1774
Final R indexes [all data]	$R1$ = 0.0872, $wR2$ = 0.1795
Largest diff. peak/hole (e/Å^3)	4.77 / -1.89

Table S2. Crystallographic information for **LaHOTP** (q = 1/3 c).

Identification code (CCDC Deposition)	2500713
Empirical formula	$La_{1.5}C_{18}H_6O_9$
Formula weight	574.6
Temperature (K)	298
Crystal system	Trigonal
Space group	$P\bar{3}c1$

a (Å)	22.1205(7)
b (Å)	22.1205(7)
c (Å)	18.3698(6)
α (°)	90
β (°)	90
γ (°)	120
Volume ($Å^3$)	7784.4(6)
Z	12
ρ_{calc} (g/cm^3)	1.471
μ (1/mm)	1.178
F(000)	3258
Crystal size (mm^3)	0.200 x 0.010 x 0.010
Radiation	Synchrotron, 0.29 Å
2Θ range for data collection (°)	1.564 to 11.954
Index ranges	$-31 \leq h \leq 31$, $-31 \leq k \leq 31$, $-25 \leq l \leq 25$
Reflections collected	39698
Independent reflections	7736
Data/restraints/parameters	7736 / 0 / 264
Goodness-of-fit on F^2	1.045
Final R indexes [I>= 2σ(I)]	$R1$ = 0.0998, $wR2$ = 0.2444
Final R indexes [all data]	$R1$ = 0.1722, $wR2$ = 0.2958
Largest diff. peak/hole ($e/Å^3$)	4.733 / -1.593

Table S3. Crystallographic information for **CeHOTP** (q = 0.240870 c).

Identification code (CCDC Deposition)	2500709
Empirical formula	$Ce_{1.5}C_{18}H_6O_9$
Formula weight	576.3
Temperature (K)	100
Crystal system	Trigonal
Space group	$P\bar{3}c1(00\gamma)0s0$
a (Å)	22.0584(10)
b (Å)	22.0584(10)
c (Å)	6.0609(2)
α (°)	90
β (°)	90
γ (°)	120
Modulation vector	(0, 0, 0.240870)
Volume ($Å^3$)	2553.97(18)
Z	4
ρ_{calc} (g/cm^3)	1.4991
μ (1/mm)	2.687

F(000)	1092
Crystal size (mm^3)	1.320 x 0.037 x 0.037
Radiation	MoKα (λ = 0.71073)
2Θ range for data collection (°)	1.85 to 32.22
Index ranges	$-31 \le h \le 31$, $-31 \le k \le 31$, $-8 \le l \le 8$, $-1 \le m \le 1$
Reflections collected	80898
Independent reflections	7825
Data/restraints/parameters/constraints	7825 / 0 / 166 / 20
Goodness-of-fit on F^2	1.4886
Final R indexes [I>= 2σ(I)]	$R1$ = 0.0935, $wR2$ = 0.2161
Final R indexes [all data]	$R1$ = 0.1248, $wR2$ = 0.2283
Largest diff. peak/hole (e/$Å^3$)	1.28 / -1.21

Table S4. Crystallographic information for **PrHOTP** (q = 1/4 c).

Identification code (CCDC Deposition)	2500712
Empirical formula	$Pr_{1.5}C_{18}H_6O_{12}$
Formula weight	625.6
Temperature (K)	100
Crystal system	Trigonal
Space group	$P\bar{3}c1(00\gamma)0s0$
Commensurate t_0	0.0625
Supercell space group	$P321$
a (Å)	22.0438(5)
b (Å)	22.0438(5)
c (Å)	6.02540(10)
α (°)	90
β (°)	90
γ (°)	120
Modulation vector	(0, 0, 0.25)
Volume ($Å^3$)	2535.65(9)
Z	4
ρ_{calc} (g/cm^3)	1.6388
μ (1/mm)	22.412
F(000)	1194
Crystal size (mm^3)	0.738 x 0.062 x 0.065
Radiation	CuKα (λ = 1.54178)
2Θ range for data collection (°)	2.31 to 75.52
Index ranges	$-27 \le h \le 27$, $-27 \le k \le 27$, $-7 \le l \le 7$, $-1 \le m \le 1$
Reflections collected	49905

Independent reflections	10435
Data/restraints/parameters/constraints	10435 / 0 / 186 / 20
Goodness-of-fit on F^2	1.6255
Final R indexes [I>= 2σ(I)]	$R1$ = 0.0838, $wR2$ = 0.1908
Final R indexes [all data]	$R1$ = 0.1486, $wR2$ = 0.2075
Largest diff. peak/hole (e/Å^3)	2.68 / -1.77

Table S5. Crystallographic information for **NdHOTP** (q = 1/2 c).

Identification code (CCDC Deposition)	2500710
Empirical formula	$Nd_{1.5}C_{18}H_6O_9$
Formula weight	582.5978
Temperature (K)	100
Crystal system	Trigonal
Space group	$P321$
a (Å)	21.9472(8)
b (Å)	21.9472(8)
c (Å)	12.1112(7)
α (°)	90
β (°)	90
γ (°)	120
Volume (Å^3)	5052.1(5)
Z	8
ρ_{calc} (g/cm^3)	1.532
μ (1/mm)	23.682
F(000)	2208
Crystal size (mm^3)	0.100 x 0.010 x 0.010
Radiation	CuKα (λ = 1.54178)
2Θ range for data collection (°)	2.324 to 74.535
Index ranges	$-27 \le h \le 26$, $-26 \le k \le 26$, $-14 \le l \le 15$
Reflections collected	85200
Independent reflections	6825
Data/restraints/parameters	6825 / 650 / 367
Goodness-of-fit on F^2	1.046
Final R indexes [I>= 2σ(I)]	$R1$ = 0.0846, $wR2$ = 0.2244
Final R indexes [all data]	$R1$ = 0.1086, $wR2$ = 0.2470
Largest diff. peak/hole (e/Å^3)	2.465 / -1.574

Table S6. Crystallographic information for **SmHOTP** ($C2/c$).

Identification code (CCDC Deposition)	2500708
Empirical formula	$Sm_{1.5}C_{18}H_6O_9$

Formula weight	591.75
Temperature (K)	293(2)
Crystal system	Monoclinic
Space group	*C*2/*c*
a (Å)	21.9654(12)
b (Å)	38.0331(13)
c (Å)	6.09294(16)
α (°)	90
β (°)	89.934(4)
γ (°)	90
Volume (Å^3)	5090.1(3)
Z	8
ρ_{calc} (g/cm^3)	1.544
μ (1/mm)	0.332
F(000)	2232
Crystal size (mm^3)	0.150 x 0.020 x 0.020
Radiation	Synchrotron, 0.29 Å
2Θ range for data collection (°)	1.559 to 11.969
Index ranges	$-28 \le h \le 29$, $-52 \le k \le 54$, $-8 \le l \le 8$
Reflections collected	20894
Independent reflections	7317
Data/restraints/parameters	7317 / 388 / 252
Goodness-of-fit on F^2	2.720
Final R indexes [I>= 2σ(I)]	*R*1 = 0.1384, *wR*2 = 0.3941
Final R indexes [all data]	*R*1 = 0.1523, *wR*2 = 0.3990
Largest diff. peak/hole (e/Å^3)	3.111 / -3.181

Table S7. Crystallographic information for **SmHOTP** ($P2_1/n$).

Identification code (CCDC Deposition)	2500711
Empirical formula	$Sm_{1.5}C_{18}H_6O_{10.5}$
Formula weight	615.8
Temperature (K)	100
Crystal system	Monoclinic
Space group	$P2_1/n$
a (Å)	6.3682(6)
b (Å)	38.704(5)
c (Å)	21.877(2)
α (°)	90
β (°)	95.771(7)
γ (°)	90
Volume (Å^3)	5362.8(10)
Z	4

ρ_{calc} (g/cm^3)	1.525
μ (1/mm)	24.875
F(000)	2328
Crystal size (mm^3)	0.075 x 0.005 x 0.005
Radiation	CuKα (λ = 1.54178)
2Θ range for data collection (°)	2.283 to 50.426
Index ranges	$-6 \leq h \leq 6$, $-38 \leq k \leq 38$, $-21 \leq l \leq 21$
Reflections collected	64320
Independent reflections	5627
Data/restraints/parameters	5627 / 966 / 530
Goodness-of-fit on F^2	1.977
Final R indexes [I>= 2σ(I)]	$R1$ = 0.2008, $wR2$ = 0.4377
Final R indexes [all data]	$R1$ = 0.2359, $wR2$ = 0.4554
Largest diff. peak/hole (e/Å^3)	2.920 / -1.556

Section S6. Simulation of diffuse scattering

Code used to simulate the diffuse scattering:

```
import sys
import os
import matplotlib.pyplot as plt
import numpy as np
import time
import random
from pylab import imshow, show, colorbar
import math
import subprocess


def generate_number(x):
    """ Get 1 or -1 with probability x """

    if random.random() < x:
        return 1
    return -1

def runmonte(grid, runnum, length, lengthz,jxy,jz,t,hx):
    """ Perform Monte Carlo of a Kagome lattice """

    j = jxy
    h = hx            # Field is turned on

    # Experimental Parameters
    k = 0 # Set to -100 for special C2/c case, called J_D in text

    # Alternative coupling schemes
    jg = 0
    jf = 0
    jk = 0

    print("Starting Monte")

    # Variables for debugging
    a1 = 0
    a2 = 0
    a3 = 0
    a2a = 0
    a2b = 0
    a4 = 0

    count = 1 * 10 ** 7       # The number of steps
    for i in range(count):
        if i == count / 4:
            print("25%")
        elif i == count / 2:
            print("50%")
        elif i == count / 4 * 3:
            print("75%")
        elif i == count - 1:
            print("100%")

        # Choose random point on grid and flip
        x = int(random.random()*length)
        y = int(random.random()*length)
        c = int(random.random()*lengthz)
        z = random.random()

        # Mapping the Kagome lattice to a square grid with every even-i even-j index removed
        if not (x % 2 == 0 and y % 2 == 0):
            # Calculate energy in an efficient way
            # Three independent lattice sites that need to be treated

            if x % 2 == 1 and y % 2 == 0:
                a1 += 1
                diff = 2*jz*grid[x][y][c]*(grid[x][y][(c+1)%lengthz]+grid[x][y][(c-1)%lengthz]) + 2 * j * grid[x][y][c] *( grid[x][(y+1)%length][c] + grid[x][(y-1)%length][c] +
grid[(x+1)%length][(y+1)%length][c] + grid[(x-1)%length][(y-1)%length][c]) #+ 2 * (h) * grid[x][y][c]

                if k != 0:
                    xp = (np.abs(grid[x][y][c] - grid[x][(y+1)%length][c] + grid[(x-1)%length][(y+1)%length][c] - grid[(x-2)%length][(y)%length][c] + grid[(x-2)%length][(y-1)%length][c] -
grid[(x-1)%length][(y-1)%length][c]) + np.abs(-grid[x][y][c] + grid[(x+1)%length][(y+1)%length][c] - grid[(x+2)%length][(y+1)%length][c] + grid[(x+2)%length][(y)%length][c] -
grid[(x+1)%length][(y-1)%length][c] + grid[x][(y-1)%length][c]))
                    xq = (np.abs(-grid[x][y][c] - grid[x][(y+1)%length][c] + grid[(x-1)%length][(y+1)%length][c] - grid[(x-2)%length][(y)%length][c] + grid[(x-2)%length][(y-1)%length][c] -
grid[(x-1)%length][(y-1)%length][c]) + np.abs(grid[x][y][c] + grid[(x+1)%length][(y+1)%length][c] - grid[(x+2)%length][(y+1)%length][c] + grid[(x+2)%length][(y)%length][c] -
grid[(x+1)%length][(y-1)%length][c] + grid[x][(y-1)%length][c]))
                    diff += k* (xp - xq)
                if jg != 0:
```

```
                diff += 2 * jg * grid[x][y][c] * (grid[(x)%length][(y+2)%length][c]+grid[(x)%length][(y-2)%length][c]+grid[(x+2)%length][(y+2)%length][c]+grid[(x-2)%length][(y-
2)%length][c])
            if jf != 0:
                diff += 2 * jf * grid[x][y][c] * (grid[(x+2)%length][(y)%length][c]+grid[(x-2)%length][(y)%length][c]-0.5*(grid[(x)%length][(y+1)%length][c]+grid[(x)%length][(y-
1)%length][c]+grid[(x+1)%length][(y+1)%length][c]+grid[(x-1)%length][(y-1)%length][c] ))

            if jk != 0:
                diff += 2 * jk * grid[x][y][c] * (grid[(x+2)%length][(y)%length][c]+grid[(x-2)%length][(y)%length][c])

            if h != 0:
                diff += -h * grid[x][y][c] * (grid[x][(y+1)%length][c]+grid[x][(y-1)%length][c])


        elif x % 2 == 0 and y % 2 == 1:
            a2 += 1
            diff = 2*jz*grid[x][y][c]*(grid[x][y][(c+1)%lengthz]+grid[x][y][(c-1)%lengthz]) + 2 * j * grid[x][y][c] *(grid[(x-1)%length][(y-1)%length][c] +
grid[(x+1)%length][(y+1)%length][c] + grid[(x+1)%length][y][c] + grid[(x-1)%length][y][c]) #+ 2 * (h) * grid[x][y][c]

            if k != 0:
                xp = (np.abs(- grid[x][y][c] + grid[(x+1)%length][(y+1)%length][c] - grid[(x+1)%length][(y+2)%length][c] + grid[x][(y+2)%length][c] - grid[(x-1)%length][(y+1)%length][c]
+ grid[(x-1)%length][(y)%length][c]) + np.abs(grid[x][y][c] - grid[(x+1)%length][(y)%length][c] + grid[(x+1)%length][(y-1)%length][c] - grid[x][(y-2)%length][c] + grid[(x-1)%length][(y-
2)%length][c] - grid[(x-1)%length][(y-1)%length][c]))
                xq = (np.abs( grid[x][y][c] + grid[(x+1)%length][(y+1)%length][c] - grid[(x+1)%length][(y+2)%length][c] + grid[x][(y+2)%length][c] - grid[(x-1)%length][(y+1)%length][c] +
grid[(x-1)%length][(y)%length][c]) + np.abs(-grid[x][y][c] - grid[(x+1)%length][(y)%length][c] + grid[(x+1)%length][(y-1)%length][c] - grid[x][(y-2)%length][c] + grid[(x-1)%length][(y-
2)%length][c] - grid[(x-1)%length][(y-1)%length][c]))
                diff += k * (xp - xq)
            if jg != 0:
                diff += 2 * jg * grid[x][y][c] * (grid[(x+2)%length][(y)%length][c]+grid[(x-2)%length][(y)%length][c]+grid[(x+2)%length][(y+2)%length][c]+grid[(x-2)%length][(y-
2)%length][c])
            if jf != 0:
                diff += 2 * jf * grid[x][y][c] * (grid[(x)%length][(y+2)%length][c]+grid[(x)%length][(y-2)%length][c]-0.5*(grid[(x+1)%length][(y)%length][c]+grid[(x-
1)%length][(y)%length][c]+grid[(x+1)%length][(y+1)%length][c]+grid[(x-1)%length][(y-1)%length][c]))
            if jk != 0:
                diff += 2 * jk * grid[x][y][c] * (grid[(x)%length][(y+2)%length][c]+grid[(x)%length][(y-2)%length][c])
            if h != 0:
                diff += -1 * h * grid[x][y][c] * (grid[(x-2)%length][y][c]+grid[(x+2)%length][y][c])#+grid[(x-2)%length][(y-2)%length]+grid[(x+2)%length][(y+2)%length]) #hx * grid[x][y] *
(x%4-1)


        elif x % 2 == 1 and y % 2 == 1:
            a3 += 1
            #(x-1,y)(x+1,y)(x,y+1)(x,y-1)
            diff = 2*jz*grid[x][y][c]*(grid[x][y][(c+1)%lengthz]+grid[x][y][(c-1)%lengthz]) +2 * j * grid[x][y][c] *(grid[(x-1)%length][(y)%length][c] + grid[(x+1)%length][(y)%length][c] +
grid[(x)%length][(y+1)%length][c] + grid[(x)%length][(y-1)%length][c]) #+ 2 * (h) * grid[x][y][c]

            if k != 0:
                xp = (np.abs(grid[x][y][c] - grid[(x+1)%length][(y)%length][c] + grid[(x+2)%length][(y+1)%length][c] - grid[(x+2)%length][(y+2)%length][c] +
grid[(x+1)%length][(y+2)%length][c] - grid[x][(y+1)%length][c]) + np.abs(-grid[x][y][c] + grid[x][(y-1)%length][c] - grid[(x-1)%length][(y-2)%length][c] + grid[(x-2)%length][(y-
2)%length][c] - grid[(x-2)%length][(y-1)%length][c] + grid[(x-1)%length][(y)%length][c]))
                xq = (np.abs(-grid[x][y][c] - grid[(x+1)%length][(y)%length][c] + grid[(x+2)%length][(y+1)%length][c] - grid[(x+2)%length][(y+2)%length][c] +
grid[(x+1)%length][(y+2)%length][c] - grid[x][(y+1)%length][c]) + np.abs(grid[x][y][c] + grid[x][(y-1)%length][c] - grid[(x-1)%length][(y-2)%length][c] + grid[(x-2)%length][(y-
2)%length][c] - grid[(x-2)%length][(y-1)%length][c] + grid[(x-1)%length][(y)%length][c]))
                diff += k * (xp - xq)
            if jg != 0:
                diff += 2 * jg * grid[x][y][c] * (grid[(x+2)%length][(y)%length][c]+grid[(x-2)%length][(y)%length][c]+grid[(x)%length][(y+2)%length][c]+grid[(x)%length][(y-2)%length][c])
            if jf != 0:
                diff += 2 * jf * grid[x][y][c] * (grid[(x+2)%length][(y+2)%length][c]+grid[(x-2)%length][(y-2)%length][c]-0.5*(grid[(x+1)%length][(y)%length][c]+grid[(x-
1)%length][(y)%length][c]+grid[(x)%length][(y+1)%length][c]+grid[(x)%length][(y-1)%length][c]))
            if jk != 0:
                diff += 2 * jk * grid[x][y][c] * (grid[(x+2)%length][(y+2)%length][c]+grid[(x-2)%length][(y-2)%length][c])
            if h != 0:
                diff += -h * grid[x][y][c] * (grid[x][(y+1)%length][c]+grid[x][(y-1)%length][c])

        else:
            a4 += 1
            print('Something went very wrong')

        # If energy is less, accept, else, accept with some probability
        if diff < 0:
            p = 1.0
        else:
            ans = math.exp(-(diff)/t)
            p = min(1.0, ans)
        num = int(z + (1-p))  # 0 if accept and 1 if reject
        grid[x][y][c] = (num*2-1) * grid[x][y][c]

    print("End Monte:",np.average(sum(sum(grid))/length**2*4.0/3.0+1.0)/2.0)
    return grid

def rundiscus(name):
    """ Create the DISCUS macro files and run """

    print("Starting DISCUS")

    # Flags to generate certain hkl slices
    nhk0 = False
    nhk1 = True
```

```
    n0kl = False

    ##################################
    # LOG
    # Increased nabs/nord from 1001 to 1501
    # Increases lots from 10x10x20 to 15x15x20
    ##################################

    a = """discus
  read
    stru %s.dis
  fourier
    xray
    wvle 0.709
    abs h
    ord k
    ll -25, -25, 0
    lr 25, -25, 0
    ul -25, 25, 0
    nabs 1501
    nord 1501
    disp off
    set aver, 0
    lots box, 15, 15, 20, 12, no #lots size nxnxn and number of lots.
    show
    run
  exit
 output
    outf %s_int_hk0.h5
    value inte
    form hdf5
    run
  exit
  exit
exit""" % (name, name)

    if nhk0:
        f = open("%s_hk0.mac"%name,'w')
        f.write(a)
        f.close()

    a = """discus
  read
    stru %s.dis
  fourier
    xray
    wvle 0.709
    abs h
    ord k
    ll -25, -25, 1
    lr 25, -25, 1
    ul -25, 25, 1
    nabs 1501
    nord 1501
    disp off
    set aver, 0
    lots box, 15, 15, 20, 12, no # comment
    show
    run
  exit
 output
    outf %s_int_hk1.h5
    value inte
    form hdf5
    run
  exit
  exit
exit""" % (name, name)

    if nhk1:
        f = open("%s_hk1.mac"%name,'w')
        f.write(a)
        f.close()

    a = """discus
  read
    stru %s.dis
  fourier
    xray
    wvle 0.709
    abs k
    ord l
    ll 0, -25, -25
    lr 0, 25, -25
    ul 0, -25, 25
    nabs 1501
    nord 1501
```

```
    disp off
    set aver, 0
    lots box, 15, 15, 20, 12, no #lots size nxnxn and number of lots.
    show
    run
 exit
 output
    outf %s_int_0kl.h5
    value inte
    form hdf5
    run
 exit
 exit
exit""" % (name, name)

    if n0kl:
        f = open("%s_0kl.mac"%name,'w')
        f.write(a)
        f.close()

    processes = []  # To store running processes

    if nhk0:
        processes.append(subprocess.Popen(['/path/to/bin/discus_suite', f'{name}_hk0.mac']))
    if nhk1:
        processes.append(subprocess.Popen(['path/to/bin/discus_suite', f'{name}_hk1.mac']))
    if n0kl:
        processes.append(subprocess.Popen(['path/to/bin/discus_suite', f'{name}_0kl.mac']))

    # Wait for all processes to finish
    for process in processes:
        process.wait()

    return

def diffus(monte,monte2,nx,nz,name,jxy,jz,t,hx):
    """ Create the .dis files for DISCUS. Requires information from the .cif file """

    # Example .dis file format
    # spcgr P1
    # cell   14.5000    14.5000    14.5000    60.0000    60.0000    60.0000
    # ncell       40,    40,     10,    86
    # atoms
    # VOID   0.0074   0.0074   1.5868   3.94784
    # VOID   0.0920   -0.0763   1.5397   3.94784
    # VOID   -0.0763   0.0920   1.4445   3.94784
    # VOID   -0.0763   0.0920   1.5397   3.94784
    # VOID   0.0920   -0.0763   1.4445   3.94784
    # VOID   0.0045   0.0045   1.3000   3.94784
    # VOID   0.0045   0.0045   1.6910   3.94784

    # Symmetry operators for P-3
    # 'x, y, z'
    # '-y, x-y, z'
    # '-x+y, -x, z'
    # '-x, -y, -z'
    # 'y, -x+y, -z'
    # 'x-y, x, -z'

    addlinker = True # Flag for inclusion of the full linker
    Hatom = True          # Flag for inclusion of hydrogen atoms

    print("Writing DIFFUS input file")

    ny = nx
    if addlinker and not Hatom:
        natom = 102
    if addlinker and Hatom:
        natom = 126
    else:
        natom = 6

    f = open('%s.dis'%name,'w')

    f.write("title %dx%dx%d,jxy=%d,jz=%d,t=%d,hx=%d\n"%(nx,ny,nz,jxy,jz,t,hx))
    f.write("spcgr P1\n")
    f.write("cell  22.0511    22.0511    6.0844    90.0000    90.0000    120.0000\n")
    f.write("ncell       %d,    %d,     %d,     %d\n"%(nx,ny,nz,natom))
    f.write("atoms\n")


    linker = """O     0.52650   0.45560   0.51670   0.02767
C    0.57460   0.43760   0.51260   0.02300
C    0.68370   0.40560   0.50240   0.01600
C    0.73880   0.38780   0.50260   0.01613
O    0.44430   0.34070   0.04740   0.04244
C    0.69930   0.47480   0.50950   0.02356
```

```
C    0.55910  0.36840  0.50350  0.01889
C    0.64710  0.49230  0.52080  0.02833
H    0.74722  0.51126  0.50654  0.02830
H    0.51124  0.33182  0.49770  0.02270
O    0.54440  0.07090  0.51670  0.02767
C    0.56240  0.13700  0.51260  0.02300
C    0.59440  0.27810  0.50240  0.01600
C    0.61220  0.35100  0.50260  0.01613
O    0.65930  0.10360  0.04740  0.04244
C    0.52520  0.22450  0.50950  0.02356
C    0.63160  0.19070  0.50350  0.01889
C    0.50770  0.15480  0.52080  0.02833
H    0.48874  0.23596  0.50654  0.02830
H    0.66818  0.17942  0.49770  0.02270
O    0.92910  0.47350  0.51670  0.02767
C    0.86300  0.42540  0.51260  0.02300
C    0.72190  0.31630  0.50240  0.01600
C    0.64900  0.26120  0.50260  0.01613
O    0.89640  0.55570  0.04740  0.04244
C    0.77550  0.30070  0.50950  0.02356
C    0.80930  0.44090  0.50350  0.01889
C    0.84520  0.35290  0.52080  0.02833
H    0.76404  0.25278  0.50654  0.02830
H    0.82058  0.48876  0.49770  0.02270
O    0.54440  0.47350  0.01670  0.02767
C    0.56240  0.42540  0.01260  0.02300
C    0.59440  0.31630  0.00240  0.01600
C    0.61220  0.26120  0.00260  0.01613
O    0.65930  0.55570  0.54740  0.04244
C    0.52520  0.30070  0.00950  0.02356
C    0.63160  0.44090  0.00350  0.01889
C    0.50770  0.35290  0.02080  0.02833
H    0.48874  0.25278  0.00654  0.02830
H    0.66818  0.48876 -0.00230  0.02270
O    0.92910  0.45560  0.01670  0.02767
C    0.86300  0.43760  0.01260  0.02300
C    0.72190  0.40560  0.00240  0.01600
C    0.64900  0.38780  0.00260  0.01613
O    0.89640  0.34070  0.54740  0.04244
C    0.77550  0.47480  0.00950  0.02356
C    0.80930  0.36840  0.00350  0.01889
C    0.84520  0.49230  0.02080  0.02833
H    0.76404  0.51126  0.00654  0.02830
H    0.82058  0.33182 -0.00230  0.02270
O    0.52650  0.07090  0.01670  0.02767
C    0.57460  0.13700  0.01260  0.02300
C    0.68370  0.27810  0.00240  0.01600
C    0.73880  0.35100  0.00260  0.01613
O    0.44430  0.10360  0.54740  0.04244
C    0.69930  0.22450  0.00950  0.02356
C    0.55910  0.19070  0.00350  0.01889
C    0.64710  0.15480  0.02080  0.02833
H    0.74722  0.23596  0.00654  0.02830
H    0.51124  0.17942 -0.00230  0.02270"""
    linker2 = """O    0.45560  0.52650  0.98330  0.02767
C    0.43760  0.57460  0.98740  0.02300
C    0.40560  0.68370  0.99760  0.01600
C    0.38780  0.73880  0.99740  0.01613
O    0.34070  0.44430  0.45260  0.04244
C    0.47480  0.69930  0.99050  0.02356
C    0.36840  0.55910  0.99650  0.01889
C    0.49230  0.64710  0.97920  0.02833
H    0.51126  0.74722  0.99346  0.02830
H    0.33182  0.51124  1.00230  0.02270
O    0.07090  0.54440  0.98330  0.02767
C    0.13700  0.56240  0.98740  0.02300
C    0.27810  0.59440  0.99760  0.01600
C    0.35100  0.61220  0.99740  0.01613
O    0.10360  0.65930  0.45260  0.04244
C    0.22450  0.52520  0.99050  0.02356
C    0.19070  0.63160  0.99650  0.01889
C    0.15480  0.50770  0.97920  0.02833
H    0.23596  0.48874  0.99346  0.02830
H    0.17942  0.66818  1.00230  0.02270
O    0.47350  0.92910  0.98330  0.02767
C    0.42540  0.86300  0.98740  0.02300
C    0.31630  0.72190  0.99760  0.01600
C    0.26120  0.64900  0.99740  0.01613
O    0.55570  0.89640  0.45260  0.04244
C    0.30070  0.77550  0.99050  0.02356
C    0.44090  0.80930  0.99650  0.01889
C    0.35290  0.84520  0.97920  0.02833
H    0.25278  0.76404  0.99346  0.02830
H    0.48876  0.82058  1.00230  0.02270
O    0.47350  0.54440  0.48330  0.02767
C    0.42540  0.56240  0.48740  0.02300
C    0.31630  0.59440  0.49760  0.01600
```

```
C    0.26120  0.61220  0.49740  0.01613
O    0.55570  0.65930  0.95260  0.04244
C    0.30070  0.52520  0.49050  0.02356
C    0.44090  0.63160  0.49650  0.01889
C    0.35290  0.50770  0.47920  0.02833
H    0.25278  0.48874  0.49346  0.02830
H    0.48876  0.66818  0.50230  0.02270
O    0.45560  0.92910  0.48330  0.02767
C    0.43760  0.86300  0.48740  0.02300
C    0.40560  0.72190  0.49760  0.01600
C    0.38780  0.64900  0.49740  0.01613
O    0.34070  0.89640  0.95260  0.04244
C    0.47480  0.77550  0.49050  0.02356
C    0.36840  0.80930  0.49650  0.01889
C    0.49230  0.84520  0.47920  0.02833
H    0.51126  0.76404  0.49346  0.02830
H    0.33182  0.82058  0.50230  0.02270
O    0.07090  0.52650  0.48330  0.02767
C    0.13700  0.57460  0.48740  0.02300
C    0.27810  0.68370  0.49760  0.01600
C    0.35100  0.73880  0.49740  0.01613
O    0.10360  0.44430  0.95260  0.04244
C    0.22450  0.69930  0.49050  0.02356
C    0.19070  0.55910  0.49650  0.01889
C    0.15480  0.64710  0.47920  0.02833
H    0.23596  0.74722  0.49346  0.02830
H    0.17942  0.51124  0.50230  0.02270"""

    uiso = 0.03200        # Uiso for the Ln atoms

    for z in range(nz):
        for y in range(ny):
            for x in range(nx):
                if monte[2*x,2*y+1,z] == 0 or monte[2*x+1,2*y+1,z] == 0 or monte[2*x+1,2*y+1,z] == 0:
                    print('Something went quite wrong')
                    return


                # La   0.56792  0.56792  0.25000  0.03200
                # La   0.43250  0.43250  0.25000  0.02590
                # La   0.43208  0.00000  0.25000  0.03200
                # La   0.56750  0.00000  0.25000  0.02590
                # La   0.00000  0.43208  0.25000  0.03200
                # La   0.00000  0.56750  0.25000  0.02590
                if monte[2*x+1,2*y,2*z] == 1:                                                    # ATOM A
                    atom1x = 0.56750
                    atom1y = 0.0000
                    atom1z = 0.250
                    f.write("La " + "{:.4f}".format(atom1x+x) + ' ' + "{:.4f}".format(atom1y+y) + ' ' + "{:.4f}".format(atom1z+z) + ' ' + "{:.4f}".format(uiso)+'\n')
                else:                                            # ATOM B
                    atom1x = 0.43208
                    atom1y = 0.0000
                    atom1z = 0.25
                    f.write("La " + "{:.4f}".format(atom1x+x) + ' ' + "{:.4f}".format(atom1y+y) + ' ' + "{:.4f}".format(atom1z+z) + ' ' + "{:.4f}".format(uiso)+'\n')

                if monte[2*x,2*y+1,2*z] == 1:                                                    # ATOM A
                    atom1x = 0.0
                    atom1y = 0.56750
                    atom1z = 0.250
                    f.write("La " + "{:.4f}".format(atom1x+x) + ' ' + "{:.4f}".format(atom1y+y) + ' ' + "{:.4f}".format(atom1z+z) + ' ' + "{:.4f}".format(uiso)+'\n')
                else:                                            # ATOM B
                    atom1x = 0.0
                    atom1y = 0.43208
                    atom1z = 0.25
                    f.write("La " + "{:.4f}".format(atom1x+x) + ' ' + "{:.4f}".format(atom1y+y) + ' ' + "{:.4f}".format(atom1z+z) + ' ' + "{:.4f}".format(uiso)+'\n')

                if monte[2*x+1,2*y+1,2*z] == 1:                                                  # ATOM A
                    atom1x = 0.43250
                    atom1y = 0.43250
                    atom1z = 0.250
                    f.write("La " + "{:.4f}".format(atom1x+x) + ' ' + "{:.4f}".format(atom1y+y) + ' ' + "{:.4f}".format(atom1z+z) + ' ' + "{:.4f}".format(uiso)+'\n')
                else:                                            # ATOM B
                    atom1x = 0.56792
                    atom1y = 0.56792
                    atom1z = 0.25
                    f.write("La " + "{:.4f}".format(atom1x+x) + ' ' + "{:.4f}".format(atom1y+y) + ' ' + "{:.4f}".format(atom1z+z) + ' ' + "{:.4f}".format(uiso)+'\n')

                # La   0.43208  0.43208  0.75000  0.03200
                # La   0.56750  0.56750  0.75000  0.02590
                # La   0.56792  0.00000  0.75000  0.03200
                # La   0.43250  0.00000  0.75000  0.02590
                # La   0.00000  0.56792  0.75000  0.03200
                # La   0.00000  0.43250  0.75000  0.02590
                if monte[2*x+1,2*y,2*z+1] == 1:                                                  # ATOM A
                    atom1x = 0.43250
                    atom1y = 0.0
                    atom1z = 0.75
```

```
                f.write("La " + "{:.4f}".format(atom1x+x) + ' ' + "{:.4f}".format(atom1y+y) + ' ' + "{:.4f}".format(atom1z+z) + ' ' + "{:.4f}".format(uiso)+'\n')
            else:                                   # ATOM B
                atom1x = 0.56792
                atom1y = 0.0
                atom1z = 0.75
                f.write("La " + "{:.4f}".format(atom1x+x) + ' ' + "{:.4f}".format(atom1y+y) + ' ' + "{:.4f}".format(atom1z+z) + ' ' + "{:.4f}".format(uiso)+'\n')

            if monte[2*x,2*y+1,2*z+1] == 1:                                 # ATOM A
                atom1x = 0.0
                atom1y = 0.43250
                atom1z = 0.75
                f.write("La " + "{:.4f}".format(atom1x+x) + ' ' + "{:.4f}".format(atom1y+y) + ' ' + "{:.4f}".format(atom1z+z) + ' ' + "{:.4f}".format(uiso)+'\n')
            else:                                   # ATOM B
                atom1x = 0.0
                atom1y = 0.56792
                atom1z = 0.75
                f.write("La " + "{:.4f}".format(atom1x+x) + ' ' + "{:.4f}".format(atom1y+y) + ' ' + "{:.4f}".format(atom1z+z) + ' ' + "{:.4f}".format(uiso)+'\n')

            if monte[2*x+1,2*y+1,2*z+1] == 1:                               # ATOM A
                atom1x = 0.56750
                atom1y = 0.56750
                atom1z = 0.75
                f.write("La " + "{:.4f}".format(atom1x+x) + ' ' + "{:.4f}".format(atom1y+y) + ' ' + "{:.4f}".format(atom1z+z) + ' ' + "{:.4f}".format(uiso)+'\n')
            else:                                   # ATOM B
                atom1x = 0.43208
                atom1y = 0.43208
                atom1z = 0.75
                f.write("La " + "{:.4f}".format(atom1x+x) + ' ' + "{:.4f}".format(atom1y+y) + ' ' + "{:.4f}".format(atom1z+z) + ' ' + "{:.4f}".format(uiso)+'\n')


            if addlinker:
                link = linker.split('\n')
                for item in range(len(link)):
                    aa = link[item].split()[0]
                    if monte2[2*x,y] == 1:
                        xa = float(link[item].split()[1])
                        ya = float(link[item].split()[2])
                    else:
                        xa = 1-float(link[item].split()[2])
                        ya = 1-float(link[item].split()[1])
                    za = float(link[item].split()[3])
                    ua = float(link[item].split()[4])
                    if aa != 'H':      # The H atoms are manually turned off
                        f.write(aa + " " + "{:.4f}".format(xa+x) + ' ' + "{:.4f}".format(ya+y) + ' ' + "{:.4f}".format(za+z) + ' ' + "{:.4f}".format(uiso)+'\n')
                    elif Hatom:          # The H atoms are manually turned off
                        f.write(aa + " " + "{:.4f}".format(xa+x) + ' ' + "{:.4f}".format(ya+y) + ' ' + "{:.4f}".format(za+z) + ' ' + "{:.4f}".format(uiso)+'\n')

                link = linker2.split('\n')
                for item in range(len(link)):
                    aa = link[item].split()[0]
                    if monte2[2*x+1,y] == 1:
                        xa = float(link[item].split()[1])
                        ya = float(link[item].split()[2])
                    else:
                        xa = 1-float(link[item].split()[2])
                        ya = 1-float(link[item].split()[1])
                    za = float(link[item].split()[3])
                    ua = float(link[item].split()[4])
                    if aa != 'H':      # The H atoms are manually turned off
                        f.write(aa + " " + "{:.4f}".format(xa+x) + ' ' + "{:.4f}".format(ya+y) + ' ' + "{:.4f}".format(za+z) + ' ' + "{:.4f}".format(uiso)+'\n')
                    elif Hatom:          # The H atoms are manually turned off
                        f.write(aa + " " + "{:.4f}".format(xa+x) + ' ' + "{:.4f}".format(ya+y) + ' ' + "{:.4f}".format(za+z) + ' ' + "{:.4f}".format(uiso)+'\n')


    f.close()


def main():
    """ Run Monte Carlo simulation, generate .dis file, generate .mac file and run DISCUS """

    # Main Variables
    t = 1                               # Temperature
    jzlist = [100]#np.linspace(0,50,6)  # Coupling in c-vector
    jxylist = [1]                       # Coupling in ab-plane
    hxlist = [0]                        # Additional parameter to bias the disorder

    runs = 1#2                             # Average over many runs

    runmontebool = True                 # Run Monte Carlo for metal
    plotter = True                      # Plot the Monte Carlo results

    n2 = 50                             # Size of lattice in ab-plane
    n3 = 100                               # Size of lattice in c-vector

    # For debugging
    #ahold = np.zeros((nx,ny))#.astype(complex)
    #aahold = np.zeros((display*2,display*2))
```

```
    for jxynum in range(len(jxylist)):
        for jznum in range(len(jzlist)):
            for hxnum in range(len(hxlist)):
                jz = jzlist[jznum]
                jxy = jxylist[jxynum]
                hx = hxlist[hxnum]

                print("Starting run ",(jznum+1),":Jz = ",jz),":Jxy = ",jxy

                # Initialize the Monte Carlo array
                monte = np.zeros((n2,n2,n3))
                monte2 = np.zeros((n2,int(n2/2)))
                for i in range(n2):
                    for j in range(n2):
                        for k in range(n3):
                            monte[i,j,k] = np.random.choice([-1,1])

                            if i%2 == 0 and j%2 == 0:
                                monte[i,j,k] = 0
                            else:
                                monte[i,j,k] = generate_number(0.5)
                realn2 = int(n2/2)
                realn3 = int(n3/2)
                for i in range(n2):
                    for j in range(int(n2/2)):
                        # Linker is manually set to no disorder
                        monte2[i,j] = 1

                if runmontebool:
                    monte = runmonte(monte,jznum,n2,n3,jxy,jz,t,hx)


                # ---------------------------------------------------------------
                # SET THIS VARIABLE TO NOT OVERRIDE DATA
                name = "publication_jkh_%d_%d_%d"%(jz,jxy,hx)                                    # Output file name
                # ---------------------------------------------------------------

                diffus(monte,monte2,realn2,realn3,name,jxy,jz,t,hx)

                if plotter == True:
                    plt.subplot(131)
                    imshow(monte[0:n2-1,0:n2-1,0])
                    plt.subplot(132)
                    imshow(monte[1,0:n2-1,0:n3-1])
                    plt.subplot(133)
                    imshow(monte2)
                    plt.savefig("%s"%name)
                rundiscus(name)
    return

if __name__ == '__main__':
    main()
```

Output of code as copied:

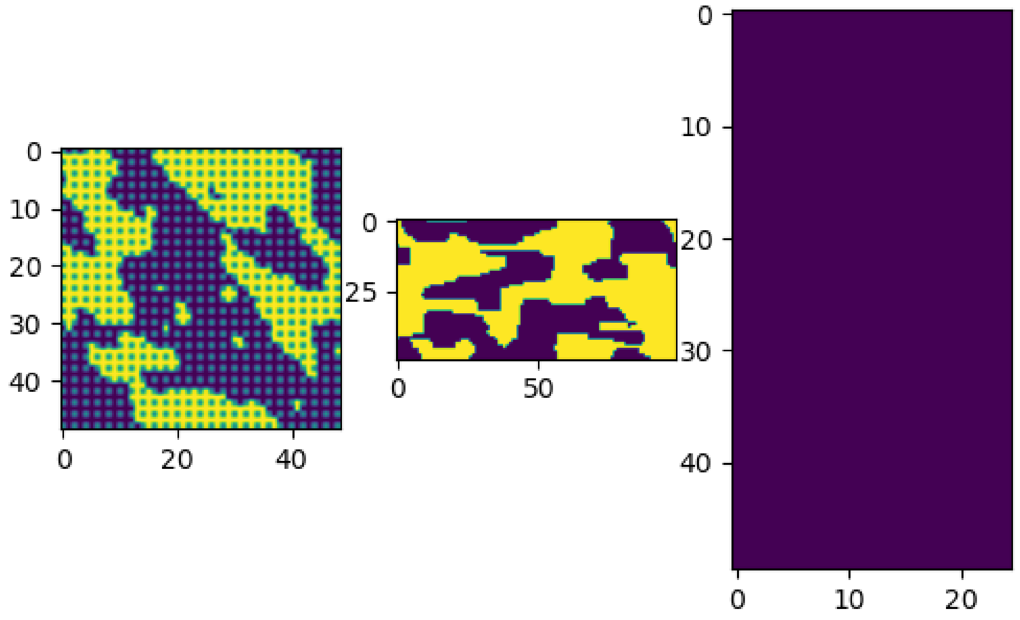

Figure S5. Representation of the disorder for a simulation with J_Z = 100 and J_{XY} = 1. (Left) The disorder of the lanthanides in an *ab*-slice. The trigonal cell is skewed by 60° in order to fit into a square box. The (even *x*, even *y*) indices are not counted by the simulation as they must be ignored in order to map the trigonal lattice onto a square grid. (Middle) The disorder of the lanthanides in a *bc*-slice (here the *y*-axis is the *b* direction and the *x*-axis is the *c* direction). (Right) The disorder of the organics. In the code supplied, the HOTP ligand is perfectly ordered.

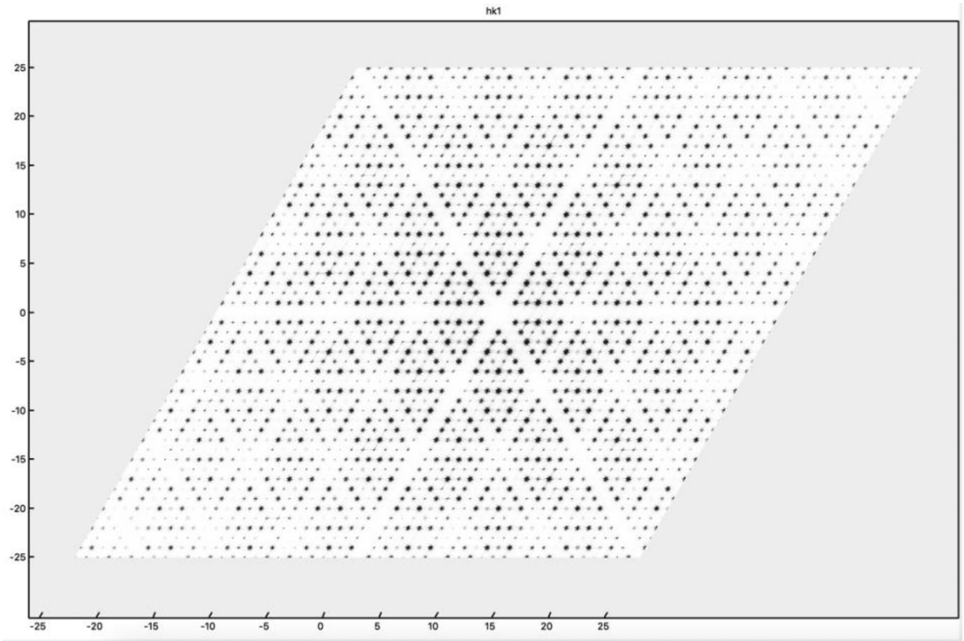

Figure S6. The output of the above code. Simulated diffuse scattering in the *hk*1 plane for J_Z = 100 and J_{XY} = 1.

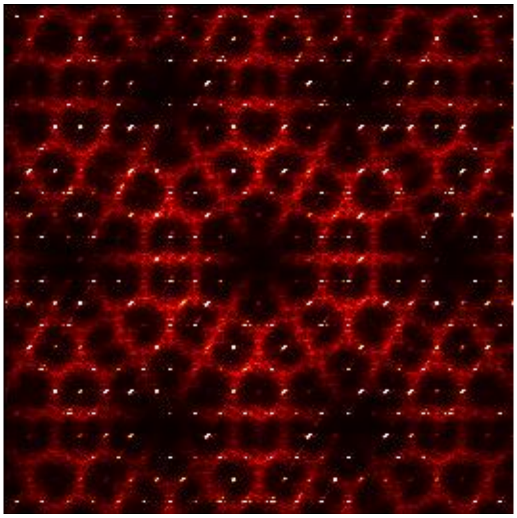

Figure S7. Simulated diffuse scattering in the *hk*1 plane for J_Z = 50 and J_{XY} = -10.

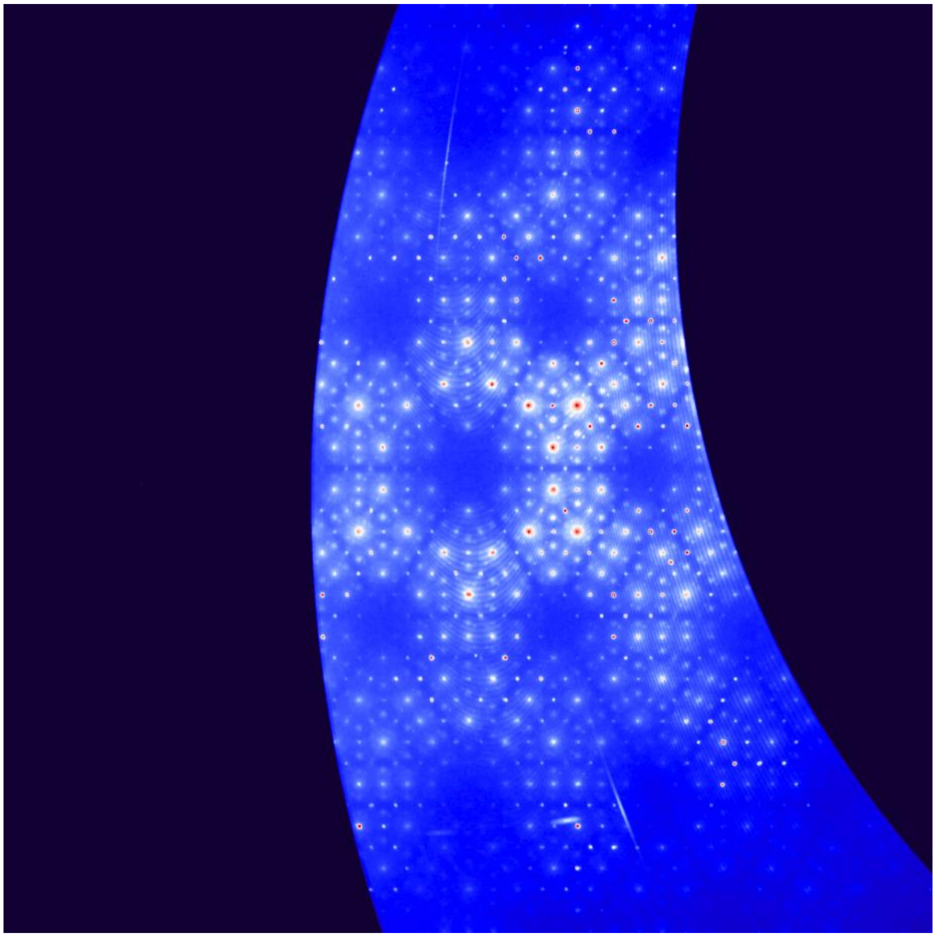

Figure S8. Simulated *hk*1 precession image for a crystal of **NdHOTP** depicting a cell tripling to a 38 Å x 38 Å x 6 Å trigonal cell.

Section S7. Continuous shape and symmetry measures

In order to explore the effect of the modulation on the local geometry of each eight-coordinate lanthanide site, continuous symmetry and shape measurements were performed on commensurate **LnHOTP** crystals. The deviation from each ideal geometry can be calculated using the SHAPE 2.1 software.[8,9] The continuous shape measure (CShM) relative to a polyhedron P for a set of N atoms can be calculated as

$$S_Q(P) = \min \frac{\sum_{i=1}^{N} |Q_i - P_i|^2}{\sum_{i=1}^{N} |Q_i - Q_0|^2} 100,$$

where Q_i is the position vector of each atom, P_i is the position vector of corresponding vertex in a reference polyhedron P, and Q_0 is the position vector of the geometrical center of the problem structure. A structure Q completely coincident in shape with the reference polyhedron P would result in a value of $S_Q(P) = 0$. The maximum value $S_Q(P) = 100$ corresponds to a scenario where all atoms of Q occupy the same point in space.

Table S8. Eight-coordinate polyhedra studied for continuous symmetry and shape measures.

Code	Point group	Polyhedron
**OP-8**	D_{8h}	Octagon
**HPY-8**	C_{7v}	Heptagonal pyramid
**HBPY-8**	D_{6h}	Hexagonal bipyramid
**CU-8**	O_h	Cube
**SAPR-8**	D_{4d}	Square antiprism
**TDD-8**	D_{2d}	Triangular dodecahedron
**JGBF-8**	D_{2d}	Johnson gyrobifastigium J26
**JETBPY-8**	D_{3h}	Johnson elongated triangular bipyramid J14
**JBTPR-8**	C_{2v}	Biaugmented trigonal prism J50
**BTPR-8**	C_{2v}	Biaugmented trigonal prism
**JSD-8**	D_{2d}	Snub diphenoid J84
**TT-8**	T_d	Triakis tetrahedron
**ETBPY-8**	D_{3h}	Elongated trigonal bipyramid

We selected three distinct polymorphs of commensurately modulated **LnHOTP** crystals with different *q* vectors: **NdHOTP** (*q* = 1/2 *c*), **LaHOTP** (q = 1/3 *c*), and **LaHOTP** (q = 1/4 *c*). In our model, all commensurate (i.e., wavevectors of 1/2 *c*, 1/3 *c*, and 1/4 *c*) crystals belong to the low-temperature superspace group $P\bar{3}c1(00\gamma)0s0$. Note that the crystals corresponding to *q* = 1/2 *c* and *q* = 1/3 *c* have been refined directly in real space (i.e., using a supercell); likewise, continuous symmetry and shape analyses could be performed in real space. On the other hand, crystals displaying *q* = 1/4 *c* were refined using the superspace formalism due to the absence of second-order satellites. Therefore, the Ln and O coordinates of the *P*321 crystals (q = 1/4 *c*) would have to be obtained from the structure averaged from the superspace formalism.

Analyses were performed on each of the distinct eight-coordinate La and Nd appearing in the crystal structure, including both major (70-80%) and minor (10-20%) occupancy sites (i.e., disordered positions). The metal atom was taken as the center of the eight-coordinate polyhedron, where the lanthanide is typically coordinated to six oxygen atoms from HOTP and two from the solvent (i.e., one water and one hydroxide). The unit cell for commensurate crystals typically contains 4 to 8 distinct lanthanide positions, including both main and disordered sites. Formally four-, six-, seven-coordinate Ln sites could not be assigned an eight-coordinate polyhedra, and were excluded from the analysis; only Ln positions unambiguously containing eight oxygen atoms in the coordination sphere were considered.

Additional continuous symmetry and shape measurements were performed on the two polymorphs of **SmHOTP**—structures consistent with *C*2/*c* and the new phase belonging to the $P2_1/n$ space group. In the case of the $P2_1/n$ polymorph, the eight-coordinate Sm atoms remain as 8-coordinate square antiprisms, but coordinate to five oxygen atoms from HOTP and three oxygens from solvent molecules (taken as either water or hydroxide).

Table S9. CShM values of commensurate **NdHOTP** (q = 1/2 c) for select eight-coordinate polyhedra. †

Structure [ML_8]	Nd01	Nd1	Nd02	Nd2	Nd03
**occ.**	0.719	0.281	0.719	0.281	0.719
**OP-8**	35.452	30.867	34.813	35.210	35.917
**HPY-8**	22.031	19.716	22.322	22.912	23.781
**HBPY-8**	10.671	9.410	10.373	10.023	11.199
**CU-8**	4.711	5.103	3.948	3.765	4.348
**SAPR-8**	3.392	5.188	3.572	4.081	4.418
**TDD-8**	2.326	3.522	2.005	3.000	3.282
**JGBF-8**	15.062	14.771	14.816	14.849	17.873
**JETBPY-8**	25.167	23.056	24.748	23.799	27.178
**JBTPR-8**	5.047	6.552	4.910	5.916	6.522
**BTPR-8**	4.765	5.995	4.819	5.769	6.253
**JSD-8**	5.846	7.205	5.182	6.699	7.844
**TT-8**	5.049	5.497	4.244	3.974	4.068
**ETBPY-8**	23.032	22.367	23.082	22.118	24.678

† Nd3 (occ. 0.281) adopts a four-coordinate square pyramidal (C_{4v}) structure with the Nd atom forming the apex of the pyramid.

Table S10. CShM values of commensurate **LaHOTP** (q = 1/3 c) for select eight-coordinate polyhedra. †

Structure [ML_8]	La1	La2	La4

**occ.**	0.824	0.824	0.176
**OP-8**	33.310	34.348	31.707
**HPY-8**	24.398	21.809	21.216
**HBPY-8**	10.765	8.706	8.640
**CU-8**	3.567	3.405	3.276
**SAPR-8**	5.031	3.997	5.618
**TDD-8**	2.24	3.174	3.962
**JGBF-8**	16.000	13.556	14.428
**JETBPY-8**	27.168	24.439	22.433
**JBTPR-8**	6.813	5.989	7.915
**BTPR-8**	5.478	5.574	7.287
**JSD-8**	7.376	7.475	8.993
**TT-8**	4.045	4.020	4.147
**ETBPY-8**	24.191	22.155	21.357

† La3 (occ. 0.176) adopts a seven-coordinate capped octahedron (C_{3v}) structure with CShM = 4.390.

Table S11. CShM values of commensurate **LaHOTP** (q = 1/4 c) for select eight-coordinate polyhedra. †

Structure [ML$_8$]	La1-1	La2-1	La2-2	La2-3	La11-1	La21-1	La21-2
**occ.**	0.163	0.837	0.837	0.837	0.163	0.837	0.837
**OP-8**	32.690	34.588	35.462	33.937	32.247	36.257	34.366
**HPY-8**	22.510	23.559	21.592	22.293	20.740	22.382	21.631
**HBPY-8**	8.428	10.809	8.151	9.272	8.773	9.231	8.119
**CU-8**	2.802	4.319	4.208	3.543	3.997	4.339	3.801
**SAPR-8**	5.764	3.650	4.040	4.514	6.156	3.748	4.374
**TDD-8**	4.098	1.621	3.757	2.297	4.670	2.267	2.754
**JGBF-8**	14.235	15.564	11.608	14.487	14.726	13.480	12.005
**JETBPY-8**	22.171	26.984	23.739	24.058	22.716	25.054	23.946
**JBTPR-8**	7.995	5.468	5.850	6.030	7.975	5.335	5.639
**BTPR-8**	7.487	4.742	5.123	5.619	7.395	4.452	5.310
**JSD-8**	8.960	6.193	7.978	6.328	9.384	7.058	6.775
**TT-8**	3.552	5.026	4.796	4.225	4.753	4.989	4.433
**ETBPY-8**	20.982	24.715	20.922	21.893	21.260	22.296	21.602

† La11-2 (occ. 0.163) adopts a seven-coordinate capped octahedron (C_{3v}) structure with CShM = 4.401. La1-2 (occ. 0.163) and La1-3 (occ. 0.163) are best described as six-coordinate trigonal prismatic (D_{3h}) structures with CShM = 7.951 and CShM = 3.915, respectively; the high value for the former indicates significant distortion from any ideal polyhedron.

Table S12. CShM values of **SmHOTP** ($C2/c$) for select eight-coordinate polyhedra. †

Structure [ML$_8$]	Sm1	Sm2	Sm3
**occ.**	0.653	0.500	0.347
**OP-8**	34.333	33.285	29.230

**HPY-8**	19.802	20.848	17.399
**HBPY-8**	11.045	10.084	11.004
**CU-8**	4.952	3.987	6.637
**SAPR-8**	3.196	4.399	5.658
**TDD-8**	3.067	3.482	4.405
**JGBF-8**	14.620	16.022	15.793
**JETBPY-8**	25.178	23.733	23.118
**JBTPR-8**	5.313	6.542	7.325
**BTPR-8**	4.974	6.140	7.042
**JSD-8**	6.941	7.824	8.588
**TT-8**	5.244	4.574	7.446
**ETBPY-8**	22.928	22.505	20.315

† Sm3 (occ. 0.347) formally adopts a seven-coordinate capped octahedron (C_{3v}) structure with CShM = 5.164. However, this table includes analysis of Sm3 with an eight-coordinate geometry, as it contained an eighth O atom relatively close to the central metal site.

Table S13. CShM values of **SmHOTP** ($P2_1/n$) for select eight-coordinate polyhedra.

Structure [ML_8]	Sm1	Sm2	Sm3
**occ.**	1.000	1.000	1.000
**OP-8**	33.701	32.389	34.394
**HPY-8**	21.736	21.845	22.278
**HBPY-8**	13.324	12.970	10.533
**CU-8**	10.685	9.926	8.753
**SAPR-8**	3.592	3.067	3.279
**TDD-8**	2.029	1.916	2.195
**JGBF-8**	11.092	11.760	10.202
**JETBPY-8**	27.335	26.761	27.891
**JBTPR-8**	2.303	2.342	3.038
**BTPR-8**	1.430	1.467	2.066
**JSD-8**	4.632	4.538	5.011
**TT-8**	11.528	10.573	9.381
**ETBPY-8**	22.624	23.819	23.318

In the shape space for eight-coordinate ML_8 complexes, note that the square antiprism and distorted dodecahedron are classified as a low-energy geometry for coordination compounds, while only a small number of structures corresponds to cubes.[5,6] Compounds can be further classified as lying along different interconversion pathways (e.g., cube–square antiprism, square antiprism–dodecahedron, and dodecahedron–cube).

We begin by examining the simplest case, commensurate **NdHOTP** (q = 1/2 c). For this crystal, the polyhedron yielding the lowest CShM was found to be the distorted dodecahedron for all distinct Nd positions, including both main and minor positions of the disorder. The sites Nd01 (occ. 0.719) and Nd02 (occ. 0.719) showed best agreement with

triangular dodecahedral coordination (CShM = 2.326 and 2.005), followed by a square-antiprismatic coordination (CShM = 3.392 and 3.572). The remaining positions Nd03 (occ. 0.719), Nd1 (occ. 0.281), and Nd2 (0.281) likewise favored the dodecahedral geometry (CShM = 3.282, 3.522, and 3.000) yet the second-best-match corresponded to the cube (CShM = 4.348, 4.103, and 3.765). Note that Nd3 (occ. = 0.281) adopts a four-coordinate square pyramidal (C_{4v}) structure with the Nd atom forming the apex of the pyramid. The CShM values are generally high enough to suggest that the structure has been significantly distorted from the ideal polyhedra. The geometry can be described as an intermediate between a distorted dodecahedron and a cube, but for certain positions, a triangular dodecahedron and a square antiprism.

In the case of commensurate **LaHOTP** (q = 1/3 c), the main positions La1 (occ. 0.824) and La2 (occ. 0.824) displayed the lowest shape measures for dodecahedral coordination (CShM = 2.24 and 3.174), and followed by a cubic geometry (CShM = 3.567 and 3.405). For the minor position La4 (occ. 0.176), the best-matching polyhedron was a cube (CShM = 3.276), followed by a second-lowest value for the distorted dodecahedron (CShM = 3.962). Square antiprismatic coordination returned higher CShM cases than the dodecahedral or cubic coordination for all cases. Sites that are not listed in the main table are not formally eight-coordinate; La11-2 (occ. 0.163) adopts a seven-coordinate capped octahedron (C_{3v}) structure with CShM = 4.401, while La1-2 (occ. 0.163) and La1-3 (occ. 0.163) are best described as six-coordinate trigonal prismatic (D_{3h}) structures with CShM = 7.951 and CShM = 3.915, respectively. As in the case of **NdHOTP** (q = 1/2 c), the CShM values are higher than what is generally reported for continuous shape and symmetry analysis, indicating substantial distortion from either ideal shape. As for the eight-coordinate sites, the local structure's deviation can be further examined using the corresponding dodecahedron–cube interconversion path. Shape map analysis indicates that each ML_8 polyhedron deviated from the minimum distortion path from CU-8 (0%) to a TDD-8 (100%) geometry; La1, La2, and La4 sites returned a deviation of 19.1%, 27.6%, and 33.8%, respectively. The main sites La1 and La2 show less pronounced deviations from the interconversion path in shape space, as compared with La4. For all cases, the geometry can be best approximated as an intermediate between a triangular dodecahedron and a cube.

The last case belongs to commensurate crystals with q = 1/4 c. In the **LaHOTP** (q = 1/4 c) sample, all major occupancy sites La2-1 (occ. 0.837), La2-2 (occ. 0.837), La2-3 (occ. 0.837), La21-1 (occ. 0.837), and La21-2 (occ. 0.837) displayed the lowest CShM for the triangular dodecahedron (CShM = 1.621, 3.757, 2.297, 2.267, and 2.754). These values were followed by square antiprismatic coordination for La2-1 and La2-2 (CShM = 3.650 and 4.040) or cubic coordination for La2-3 and La21-2 (CShM = 3.543 and 3.801). On the other hand, the minor occupancy sites La1-1 (occ. 0.163) and La11-1 (occ. 0.163) were shown to be primarily cubic (CShM = 2.802 and 3.801), followed by a triakis tetrahedron (CShM = 3.552) and a triangular dodecahedron (CShM = 4.670), respectively. La1-2 (occ. 0.163) and La1-3 (occ. 0.163) are best described as six-coordinate trigonal prismatic (D_{3h}) structures

with CShM = 7.951 and CShM = 3.915, respectively; the notably high value for the former indicates significant distortion from any ideal polyhedron. Although the q = 1/4 c case is structurally more complicated than the previous two cases, the typical coordination environment can similarly be described as a combination of dodecahedral, cubic, and square antiprismatic coordination.

The effect of the periodic lattice distortion, when coupled with various disorders, appears to be more subtle than the application of simple torsion that transforms a cube into a square antiprism. Oxygen atoms are shared between neighboring positions (c direction) and between major/minor occupancy sites in the *ab*-plane, giving rise to strong correlations. The CShM captures this aspect of the modulation, leading to local geometries consistent with an intermediate between a distorted dodecahedron and a square antiprism/cube.

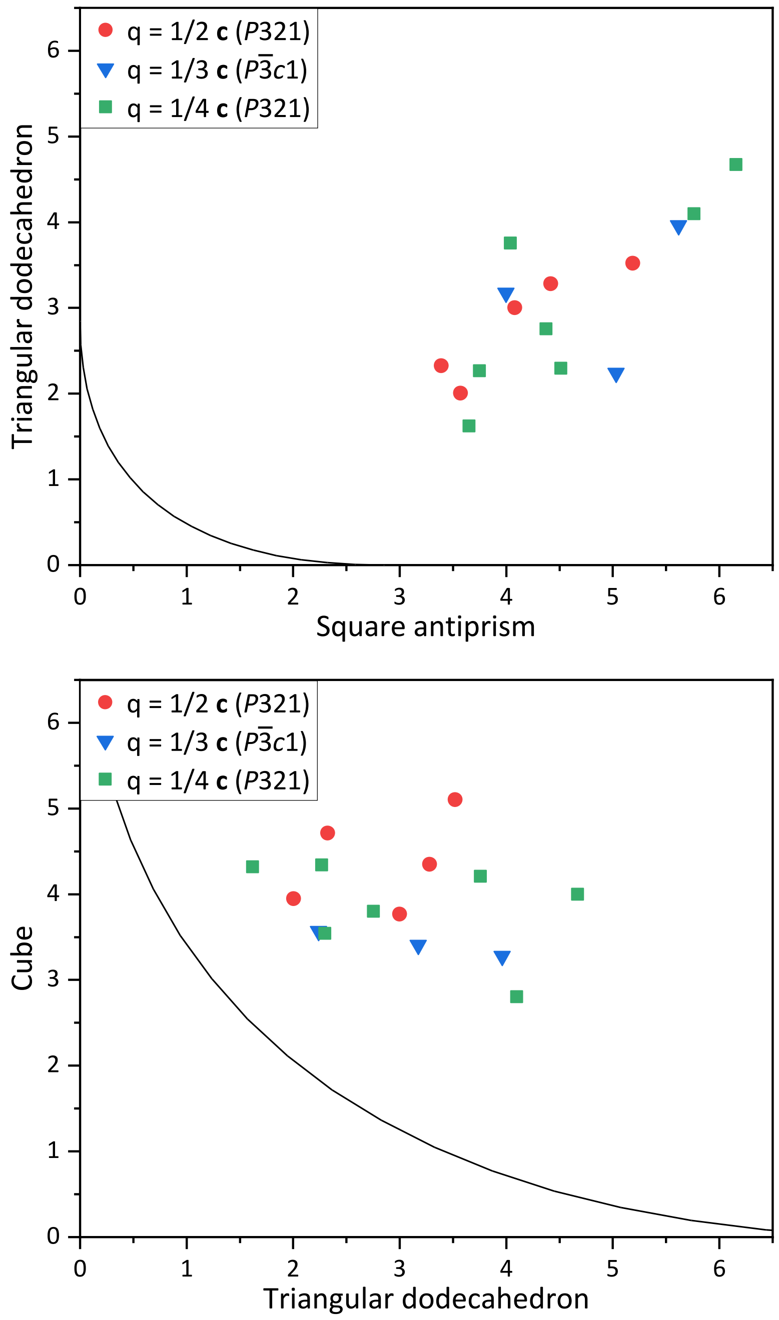

Figure S9. Shape map in the square antiprism–triangular dodecahedron space (top) and in the triangular dodecahedron–cube space (bottom) for commensurately modulated **LnHOTP** crystals. Select positions of Ln atoms corresponding to commensurate *P*321 (*q* = 1/2 *c*) **NdHOTP**, *P*-3*c*1 (*q* = 1/3 *c*) **LaHOTP**, and *P*321 (*q* = 1/4 *c*) **LaHOTP** are marked in red, blue, and green, respectively. The continuous lines correspond to the interconversion paths (minimum distortion paths) between the two reference polyhedra.

In the previous discussion, we explored the effect of the periodic lattice distortion in commensurate crystals with different modulation vectors (q = 1/2 c, 1/3 c, and 1/4 c). There were also slight differences in the preferred coordination for different sites, partially due to occupational disorder. As for the two polymorphs of **SmHOTP**, continuous symmetry and shape measures offer a handle to qualitatively investigate the effect of *positional* disorder. Measurements indicate that the $C2/c$ polymorph of **SmHOTP** generally displays results similar to the other commensurate hexagonal phase **LnHOTP** (q = 1/2 c, 1/3 c, and 1/4 c) MOFs. The sites Sm1, Sm2, and Sm3 showed best agreement with a triangular dodecahedral coordination (CShM = 3.067, 3.482, and 4.405). For Sm1 and Sm3, the second-best-match was found to be the square antiprism (CShM = 3.196 and 5.658), while for Sm2, it was the cube (CShM = 3.987).

In the case of the $P2_1/n$ polymorph, the positional disorder results in a structure with close Sm-Sm dimeric pairs. The phase is similar to the $C2/c$ structures but involves an additional descent in symmetry caused by shifts of the position of the Sm along the c axis. Contrary to the previous cases, the Sm sites of the $P2_1/n$ polymorph display the lowest values for the biaugmented trigonal prism; Sm1, Sm2, and Sm3 return CShM values of 1.430, 1.467, and 2.066, respectively. While a triangular dodecahedron (D_{2d}) consists of a pair of interpenetrating tetrahedra, the biaugmented trigonal prism (C_{2v}) is composed of a trigonal prism that is capped on two of the rectangular faces. This type of coordination is better known as the bicapped trigonal prismatic geometry, and is often encountered in the bromides or iodides of lanthanide/actinide complexes. The measures obtained for the bicapped trigonal prism are among the lowest that were calculated for the ideal polyhedra throughout the **LnHOTP** series, showing good agreement with the model geometry. In the $P2_1/n$ polymorph, the bicapped trigonal prismatic coordination effectively allows the Sm atoms in the dimeric pairs to be brought close to one another. The second-best-match for Sm1, Sm2, and Sm3 equally returned the triangular dodecahedron (CShM = 2.029, 1.916, and 2.195). The values are somewhat higher for square antiprismatic coordination (CShM = 3.067-3.592). Cubic coordination, on the other hand, yields extremely high values (CShM = 8.753-10.685), *unlike* the other structures reported in this study. Additional shape map analysis was performed to measure each site's extent of deviation from the minimum distortion path from TDD-8 (0%) to a BTPR-8 (100%) geometry; interestingly, Sm1, Sm2, and Sm3 sites returned significant deviations of 58.7%, 57.2%, and 76.9%, respectively. The substantial deviations from the interconversion path imply that the structure does not lie on a pathway that transforms a dodecahedron into a bicapped trigonal prism. This suggests that it would be difficult to induce single-crystal-to-single-crystal transformations via simple ligand association/dissociation although the two polymorphs (i.e., $C2/c$ and $P2_1/n$) have identical stoichiometries. There exists no simple minimal energy pathway that converts one polyhedron from another, although there are numerous factors that can determine the fate of the polymorph (e.g., oxidation state of the linker, shear planes from twinning).

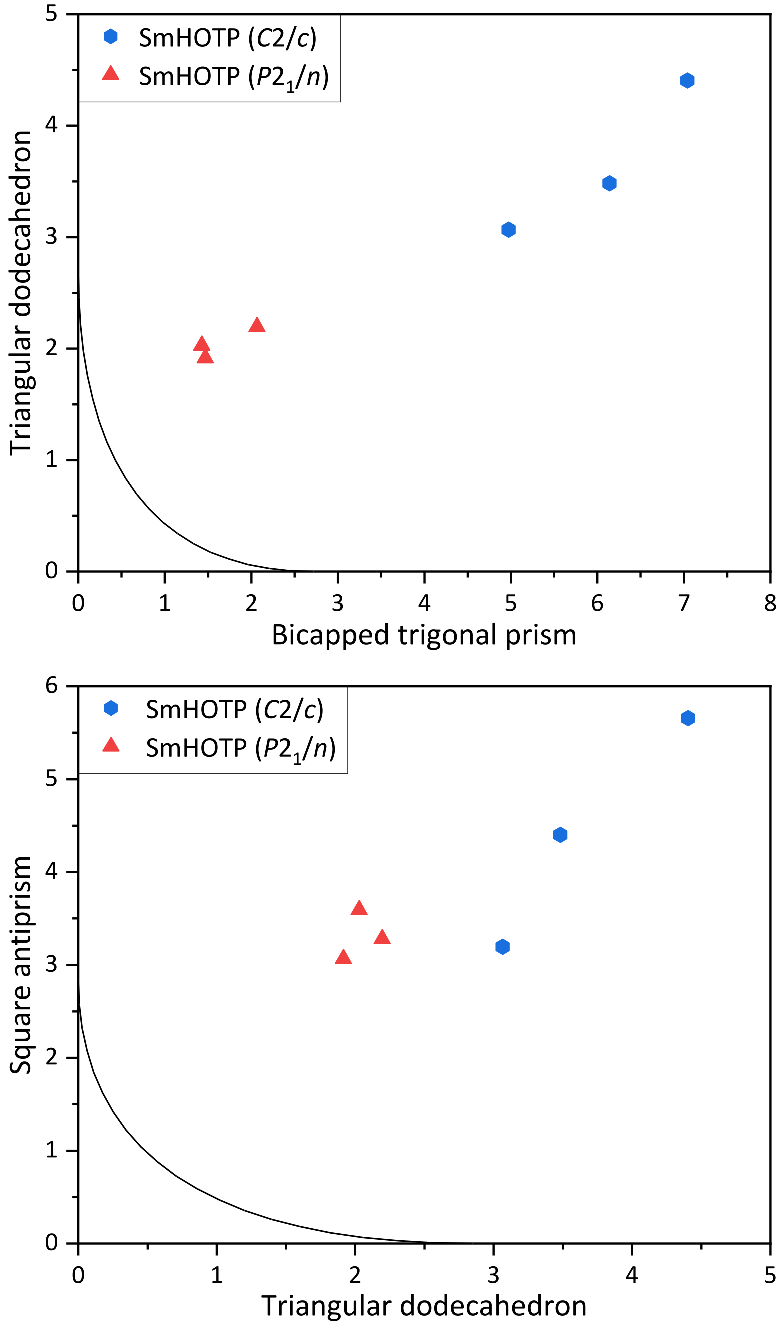

Figure S10. Shape map in the bicapped trigonal prism–triangular dodecahedron space (top) and in the triangular dodecahedron–square antiprism space (bottom) for the two polymorphs of **SmHOTP**. Select positions of Sm atoms corresponding to *C*2/*c* and *P*2$_1$/*n* are marked in blue and red, respectively. The continuous line corresponds to the interconversion path (minimum distortion path) between the two reference polyhedra.

Section S8. Variable pressure diffraction

The ruby fluorescence for the variable pressure single crystal diffraction was fit to the following equation based upon two Lorentzian functions with a linear background correction:

$$I = a_1 + a_2\lambda + a_3 \frac{a_4^2}{a_4^2 + 4(a_5 - \lambda)^2} + a_6 \frac{a_7^2}{a_7^2 + 4(a_8 - \lambda)^2}$$

In ruby, the energy difference of the excited ^2E levels is 29cm^{-1}, which leads to the splitting of R_1 and R_2 lines in the fluorescence spectra. Amongst the two peaks, R_1 is the stronger-intensity, lower-energy excitation used to gauge pressure. The position of the R_1 peak was used to probe the pressure within the cell. When possible, the pressure was taken as the average from before and after the diffraction measurement.

The pressure was fit to the equation $P = \frac{1904}{7.665}\left(\left(\frac{\lambda}{\lambda_0}\right)^{7.665} - 1\right)$ where $\lambda_0 = 694.25\ \mathrm{nm}$ corresponds to the R_1 wavelength measured at 1 atm.[11,12]

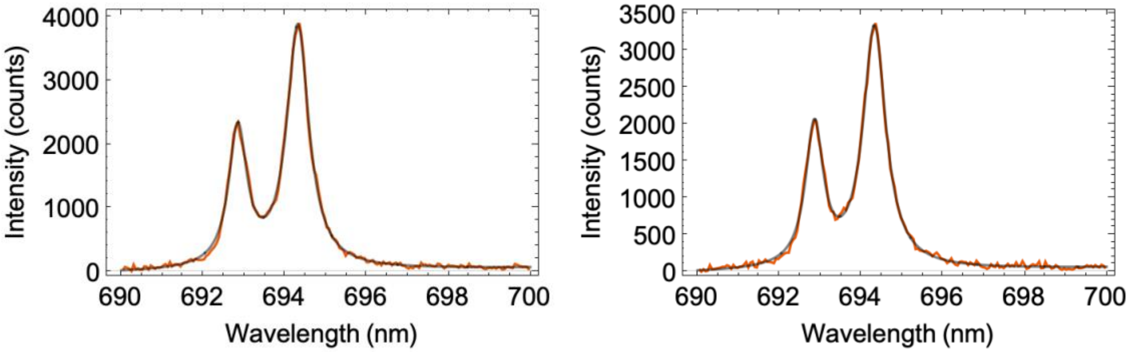

Figure S11. Ruby fluorescence before and after data point 1 for **LaHOTP**.

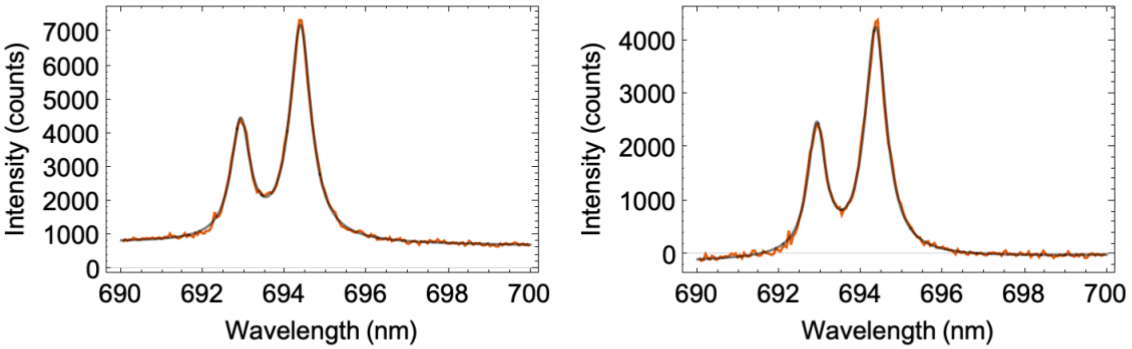

Figure S12. Ruby fluorescence before and after data point 2 for **LaHOTP**.

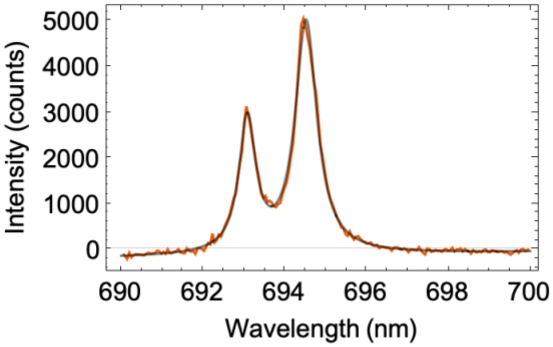

Figure S13. Ruby fluorescence after data point 3 for **LaHOTP**.

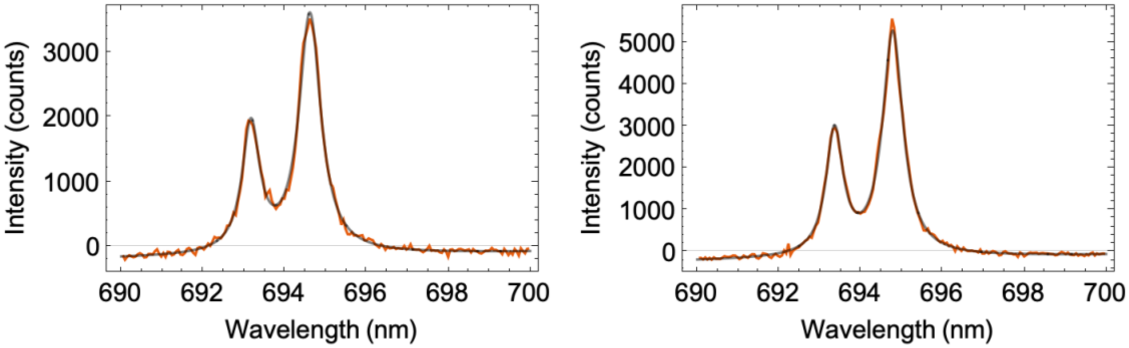

Figure S14. Ruby fluorescence before and after data point 4 for **LaHOTP**.

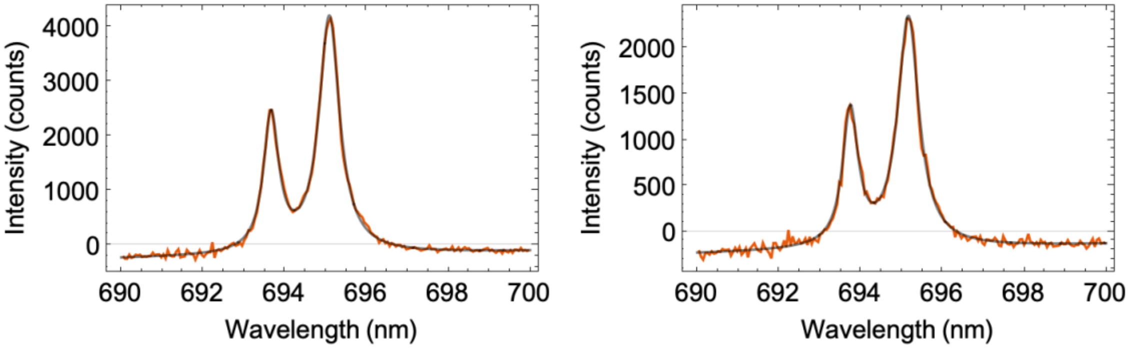

Figure S15. Ruby fluorescence before and after data point 5 for **LaHOTP**.

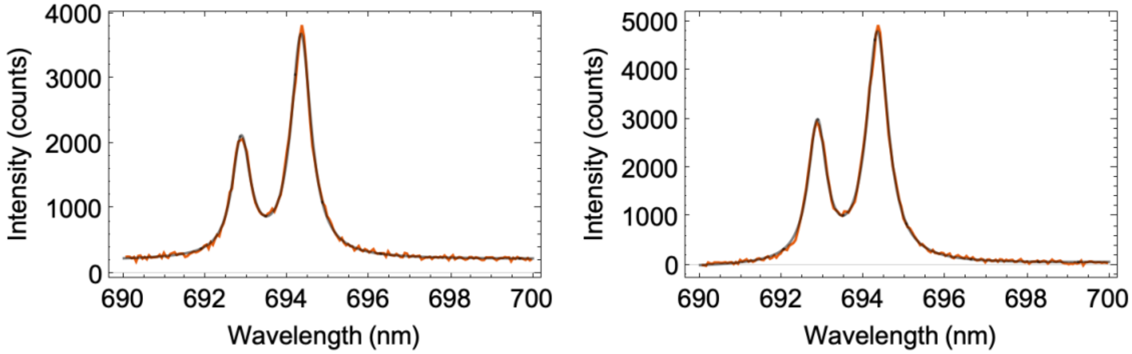

Figure S16. Ruby fluorescence before and after data point 1 for **CeHOTP**.

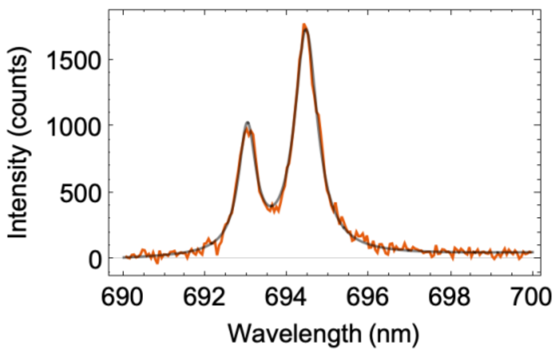

Figure S17. Ruby fluorescence after data point 2 for **CeHOTP**.

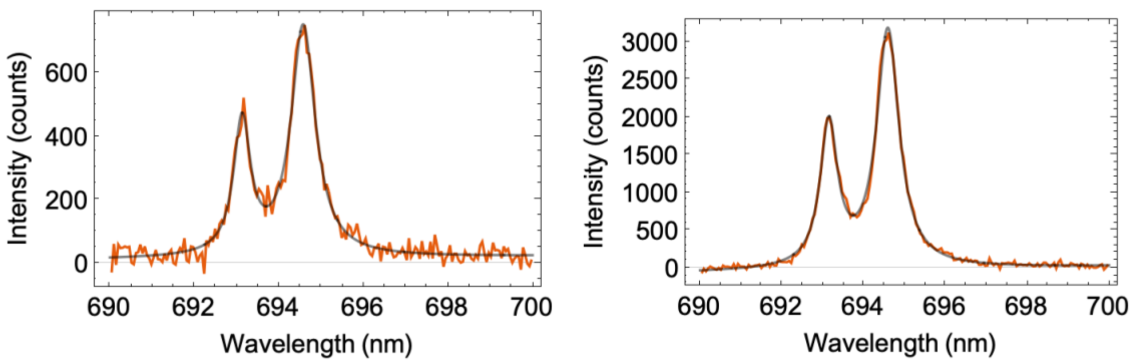

Figure S18. Ruby fluorescence before and after data point 3 for **CeHOTP**.

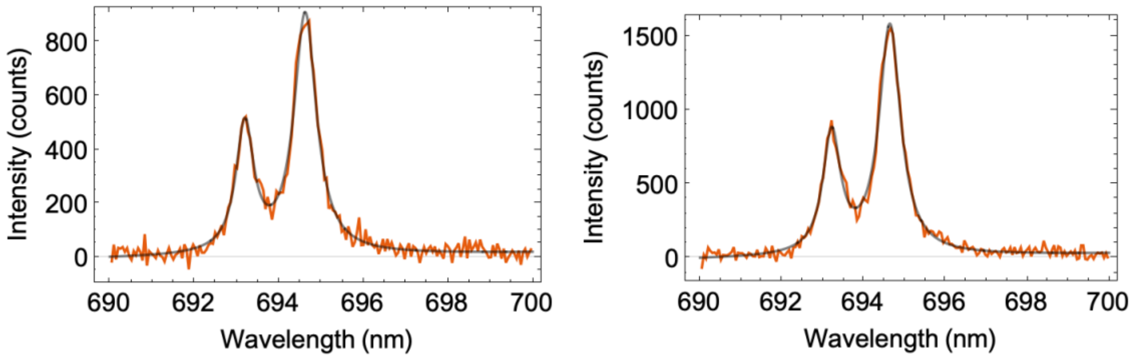

Figure S19. Ruby fluorescence before and after data point 4 for **CeHOTP**.

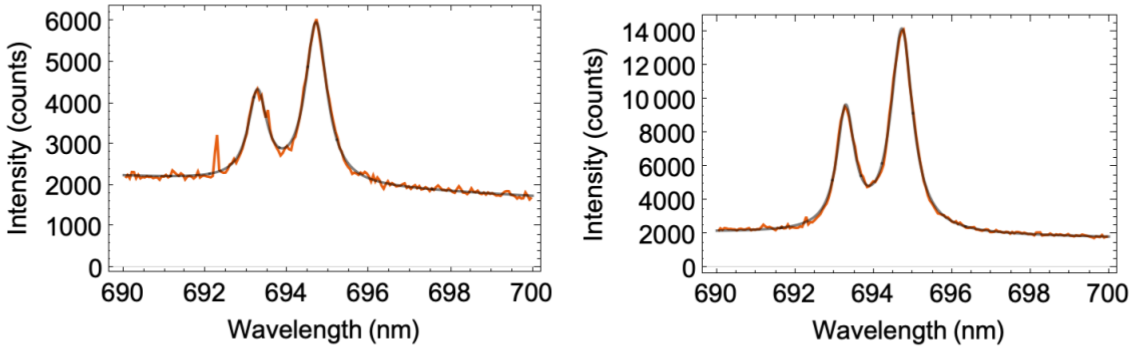

Figure S20. Ruby fluorescence before and after data point 5 for **CeHOTP**.

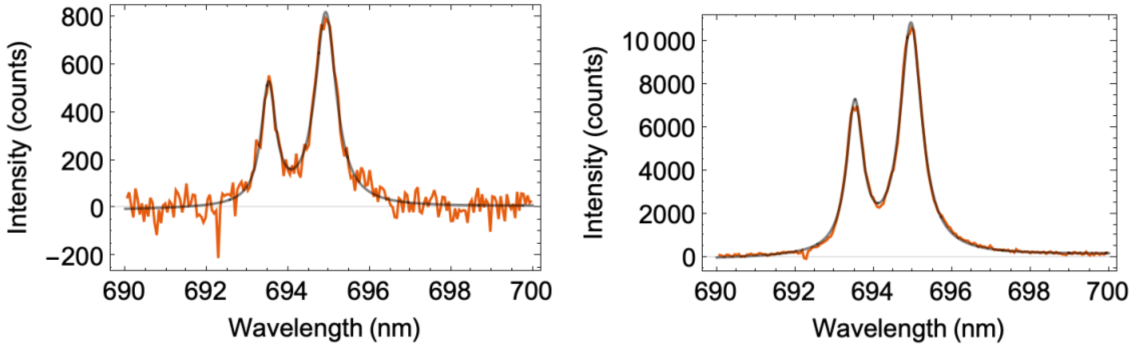

Figure S21. Ruby fluorescence before and after data point 6 for **CeHOTP**.

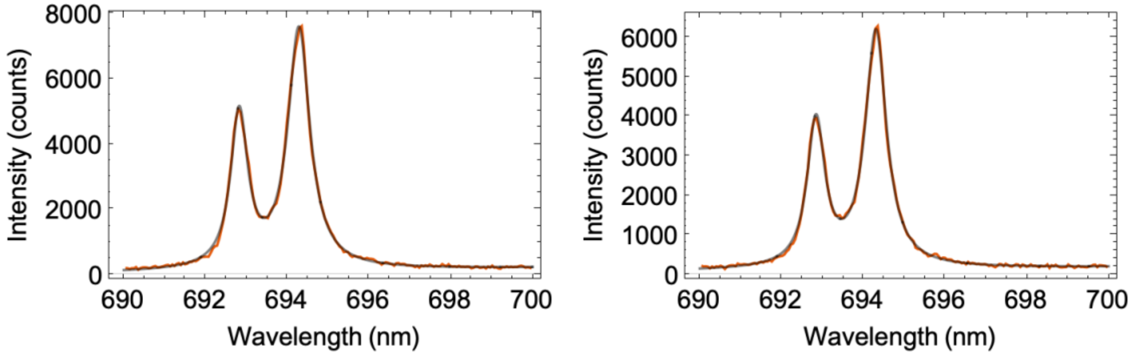

Figure S22. Ruby fluorescence before and after data point 1 for **NdHOTP**.

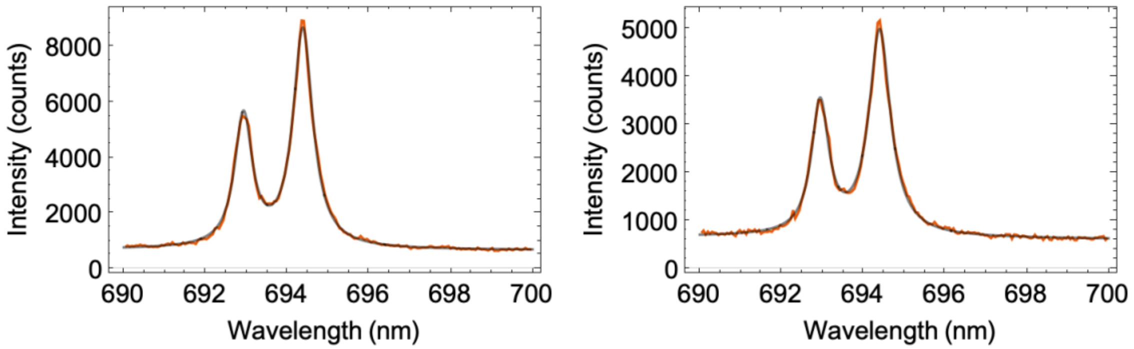

Figure S23. Ruby fluorescence before and after data point 2 for **NdHOTP**.

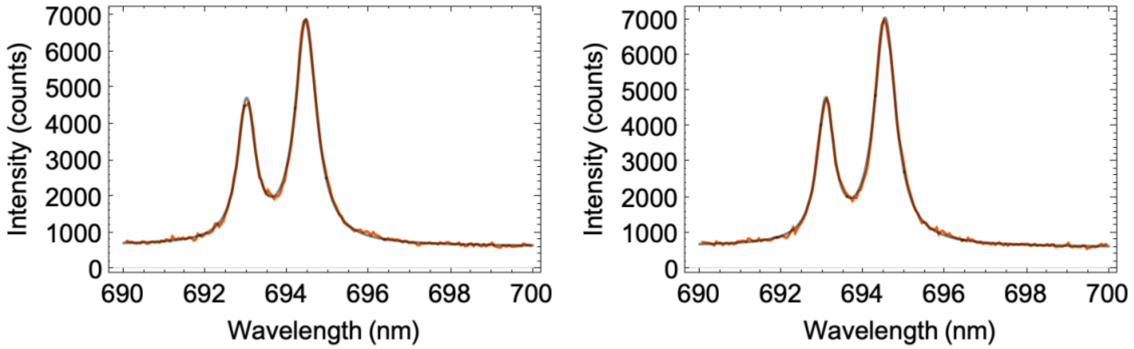

Figure S24. Ruby fluorescence before and after data point 3 for **NdHOTP**.

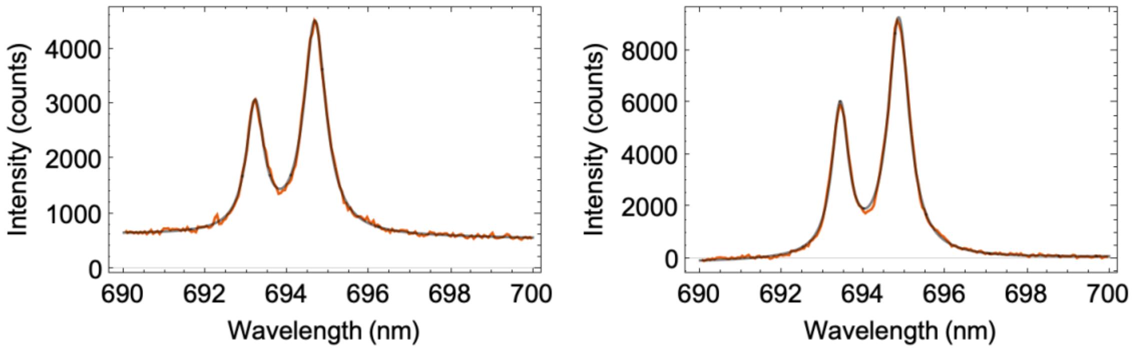

Figure S25. Ruby fluorescence before and after data point 4 for **NdHOTP**.

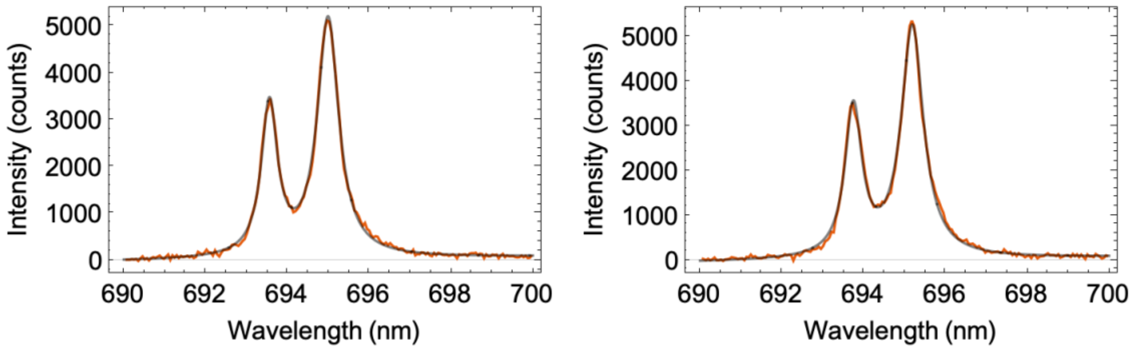

Figure S26. Ruby fluorescence before and after data point 5 for **NdHOTP**.

Synchrotron high-pressure powder X-ray diffraction.

High-pressure powder X-ray diffraction (HPPXRD) experiments were carried out at the PETRA III synchrotron facility (beamline P02.1) at the Deutsches Elektronen-Synchrotron (DESY, Hamburg), using an X-ray energy of 60 keV (λ = 0.207349 Å) and a Varex4343 CT 2D area detector for data collection. Prior to HPPXRD experiments, the sample-to-detector distances have been calibrated using Si.

The high-pressure cell used in these experiments is a custom-built prototype inspired by the pressure-jump cell described in the literature.[13] For the HPPXRD measurements, samples of **LnHOTP** (Ln = La, Ce, and Nd) were loaded into soft Kapton capillaries (MicroLumen® 730-1, inner diameter: 1.86 mm, wall thickness: 0.0368 mm), together with silicone oil AP-100, which served as a non-penetrating pressure-transmitting medium to ensure hydrostatic conditions. The capillaries were sealed with a two-component epoxy adhesive (Araldyte-2014-1).

The sealed capillaries were placed into a water-filled metal block that serves as a sample chamber. Upon sealing the chamber, the plastic capillary is brought into direct contact with the surrounding water and pressure was applied by increasing the water pressure inside the chamber. The high-pressure cell was equipped with two pressure transducers to monitor the internal water pressure.

Within the operational pressure range of the cell and at temperatures above 0 °C, water behaves as a hydrostatic pressure-transmitting medium, ensuring uniform pressure distribution throughout the high-pressure system. As the water comes in directly contact with the flexible plastic capillary, the applied pressure is transmitted through the capillary wall to the interior, to the silicone oil and the sample.

Diffraction data were collected through two diamond windows integrated into the metal block, allowing X-ray access to the sample under pressure. HPPXRD measurements were performed from ambient pressure to 0.68 GPa by raising the pressure by increments of 0.04 GPa.

Pawley profile fit analyses of the data were performed using TOPAS v6 to extract lattice parameters and volumes as a function of pressure (Table S14 – S16).

Bulk modulus of the samples were obtained by fitting the 2nd and 3rd order Birch-Murnaghan (BM) equation using PASCal developed by M. Cliffe[14] as well as EoSFit7c developed by R.J. Angel.[15]

Table S14. Results for Pawley refinements from variable-pressure powder diffraction of **CeHOTP**.

Pressure (bar)	Rwp	GoF	a (Å)	a_{error} (Å)	c (Å)	c_{error} (Å)
1	0.68115	0.43284	21.98605	0.00264	6.09474	0.0043
400	0.63705	0.40459	21.97482	0.00243	6.07948	0.00421
800	0.60853	0.38401	21.96854	0.00228	6.07292	0.00386
1200	0.56717	0.35686	21.9552	0.0021	6.06168	0.00379
1600	0.54653	0.34208	21.9415	0.00205	6.05004	0.00371
2000	0.5443	0.34211	21.93488	0.00206	6.03759	0.00391
2400	1.61997	1.01107	21.93488	0.006	6.03759	0.01151
2800	0.50589	0.31567	21.93271	0.00204	6.01866	0.00362
3200	0.5485	0.34212	21.95359	0.00238	6.00764	0.00401
3600	0.52089	0.32425	21.94441	0.00243	5.99858	0.00419
4000	1.42184	0.88266	21.94441	0.00656	5.99858	0.01139
4400	0.46263	0.28341	21.94881	0.00252	5.98467	0.00415
4800	0.41145	0.25293	21.95768	0.00235	5.97378	0.00343
5200	0.46736	0.28885	21.95908	0.00279	5.97406	0.00468
5600	0.39939	0.24409	21.96087	0.00251	5.95813	0.0038
6000	0.38407	0.23654	21.96229	0.00251	5.94883	0.00393
6400	0.36443	0.22206	21.96122	0.00243	5.94506	0.00358
6800	0.34078	0.20807	21.96681	0.00238	5.93244	0.00346

Table S15. Results for Pawley refinements from variable-pressure powder diffraction of **NdHOTP**.

Pressure (bar)	Rwp	GoF	a (Å)	a_{error} (Å)	c (Å)	c_{error} (Å)
1	1.58674	1.0241	22.02867	0.00257	6.09118	0.00085
400	1.50144	0.96898	22.0444	0.00241	6.08454	0.00088
800	1.61166	1.04206	22.04354	0.00251	6.07495	0.0012
1200	1.72018	1.10306	22.03157	0.00276	6.06558	0.00122
1600	1.83059	1.17737	22.03626	0.00288	6.0566	0.00148
2000	1.77368	1.13972	22.0229	0.00267	6.04496	0.00187
2400	1.86602	1.19654	22.01118	0.00273	6.03605	0.00191
2800	1.87074	1.19441	21.98539	0.00283	6.02617	0.00225
3200	1.94716	1.25388	21.99407	0.00299	6.01233	0.00276
3600	1.97284	1.26475	21.95441	0.00302	5.99993	0.00323
4000	1.99151	1.28632	21.95263	0.00321	5.98772	0.00396
4400	1.92166	1.23793	21.94803	0.00317	5.98015	0.00383
4800	1.93321	1.24319	21.93879	0.00319	5.97406	0.00368
5200	1.93388	0.96416	21.93368	0.00316	5.96829	0.00392

5600	1.93071	1.24022	21.93083	0.00322	5.96537	0.00429
6000	1.89404	1.21385	21.92306	0.0031	5.958	0.00344
6400	1.87344	1.20254	21.91558	0.0031	5.95151	0.00301
6800	1.82031	1.17489	21.91294	0.00303	5.946	0.00269

Table S16. Results for Pawley refinements from variable-pressure powder diffraction of **PrHOTP**.

Pressure (bar)	Rwp	GoF	a (Å)	a_{error} (Å)	c (Å)	c_{error} (Å)
0	1.21176	0.76659	22.02807	0.00135	6.084	0.00027
400	2.73627	1.72409	22.02807	0.00301	6.084	0.00061
800	1.01599	0.63181	22.00278	0.00123	6.06209	0.00031
1200	2.50379	1.55665	22.00278	0.003	6.06209	0.00077
1600	3.6744	2.29633	22.00278	0.00445	6.06209	0.00114
2000	0.84734	0.52915	21.97211	0.00111	6.03434	0.00039
2400	1.0273	0.64145	21.97344	0.0014	6.02547	0.00064
2800	2.94651	1.82901	21.97344	0.00398	6.02547	0.00185
3200	1.105	0.6803	21.98105	0.00156	6.01129	0.00078
3600	0.85707	0.52565	21.94564	0.00133	6.00143	0.00069
4000	0.97161	0.59826	21.94045	0.00155	5.99434	0.00084
4400	0.93412	0.57629	21.94262	0.0015	5.98603	0.00078
4800	1.01662	0.62647	21.92932	0.00178	5.97949	0.00094
5200	0.99188	0.61125	21.93378	0.00188	5.97357	0.0011
5600	0.85659	0.52564	21.92942	0.002	5.96555	0.00114
6000	0.7386	0.45199	21.92663	0.00203	5.95851	0.00116
6400	0.65917	0.40009	21.92581	0.00211	5.95296	0.00124
6800	0.58535	0.35574	21.92186	0.00208	5.94683	0.00118

Table S17. Bulk moduli fitted by 2nd and 3rd order Birch-Murnaghan equations using PASCal and EoSFit7c.

BM equation	CeHOTP	PrHOTP	NdHOTP
PASCal 2nd order	23.6 ± 1.2 GPa	18.3 ± 0.7 GPa	15.7 ± 0.6 GPa
PASCal 3rd order	8.1 ± 4.3 GPa	9.9 ± 3.4 GPa	11.9 ± 3.2 GPa
EoSFit 2nd order	23.6 ± 1.2 GPa	18.9 ± 0.7 GPa	15.7 ± 0.6 GPa
EoSFit 3rd order	8.1 ± 4.3 GPa	9.9 ± 3.4 GPa	11.9 ± 3.2 GPa

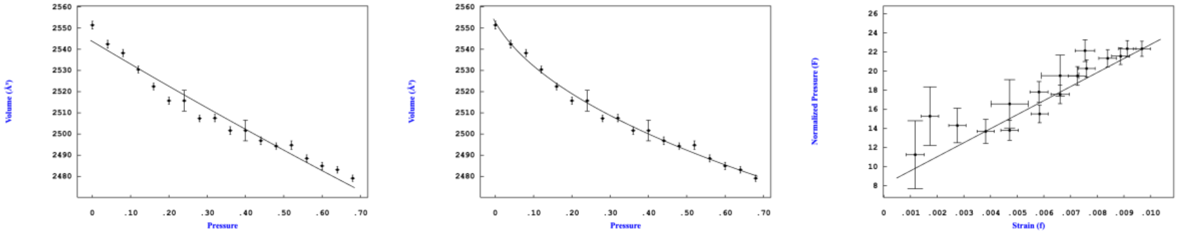

Figure S27. *Vp*-plots 2nd order (left), *Vp*-plots 3rd order (middle) and *Ff*-plots (right) of CeHOTP.

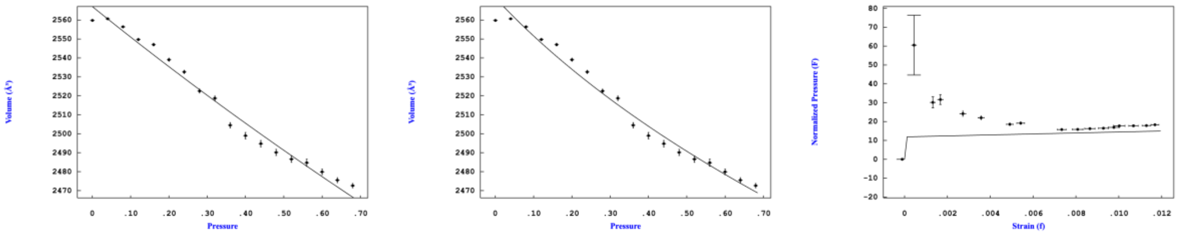

Figure S28. *Vp*-plots 2nd order (left), *Vp*-plots 3rd order (middle) and *Ff*-plots (right) of NdHOTP.

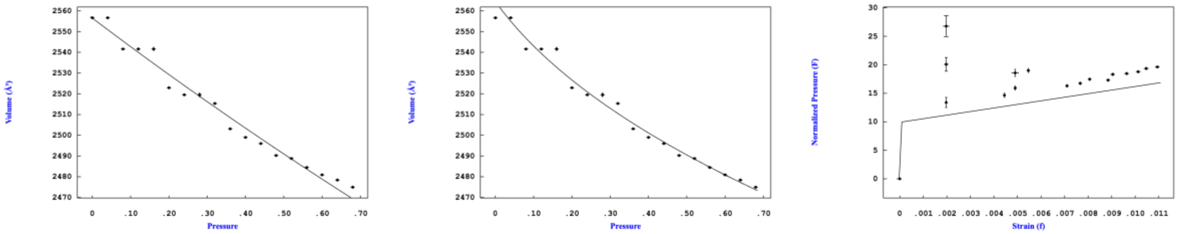

Figure S29. *Vp*-plots 2nd order (left), *Vp*-plots 3rd order (middle) and *Ff*-plots (right) of PrHOTP.

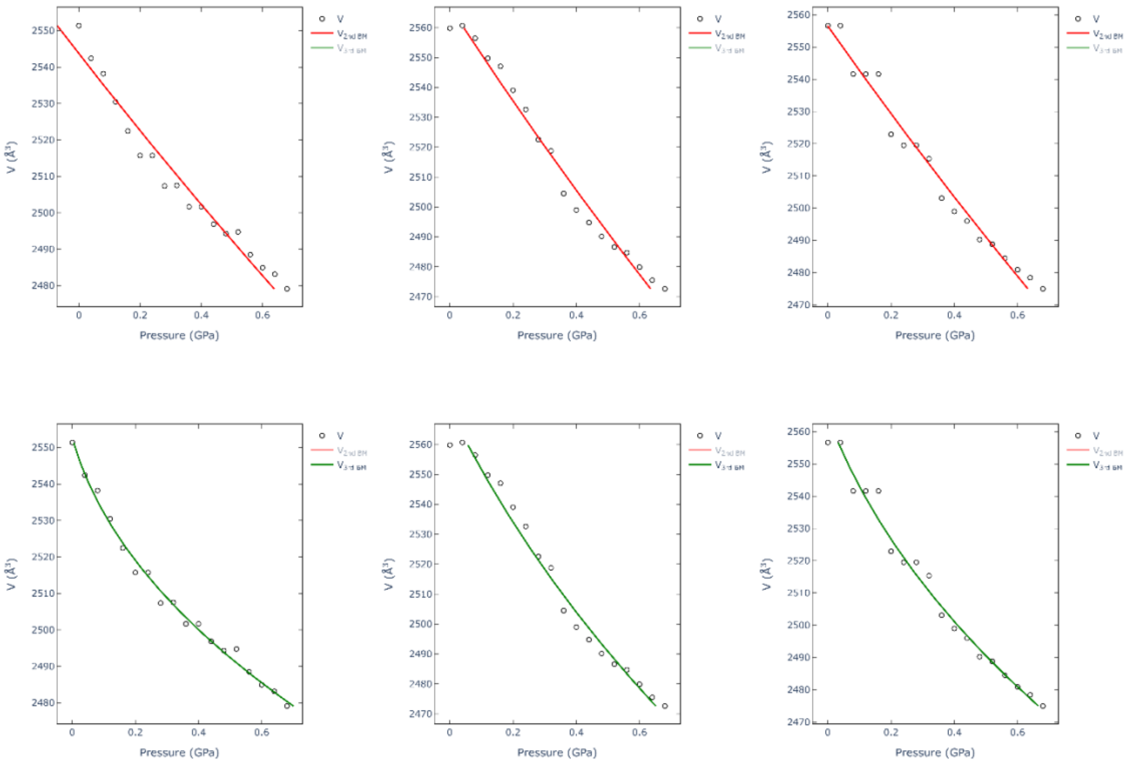

Figure S30. *Vp*-plots 2nd order (top), *Vp*-plots 3rd order (bottom) of CeHOTP (left), NdHOTP (middle) and PrHOTP (right)

The bulk modulus was calculated from 2nd and 3rd order Birch-Murnaghan Coefficients using the following equation: $P = \frac{3}{2}B_0((\frac{V_0}{V})^{\frac{7}{3}} - (\frac{V_0}{V})^{\frac{5}{3}})(1 + \frac{3}{4}(B_0' - 4)((\frac{V_0}{V})^{2/3} - 1))$

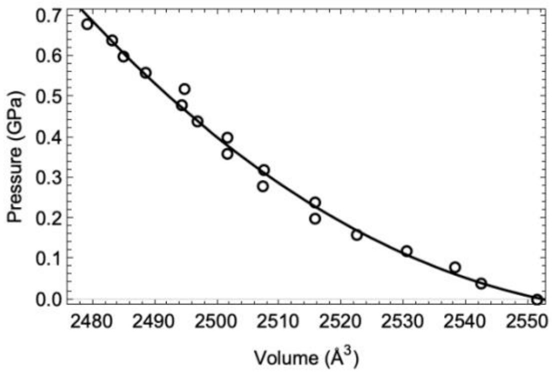

Figure S31. Bulk modulus fit for **CeHOTP**. B_0 = 8.1 ± 4.3 GPa, B_0’ = 124.8 ± 79.0 GPa, V_0 = 2552.8 ± 5.3 Å^3.

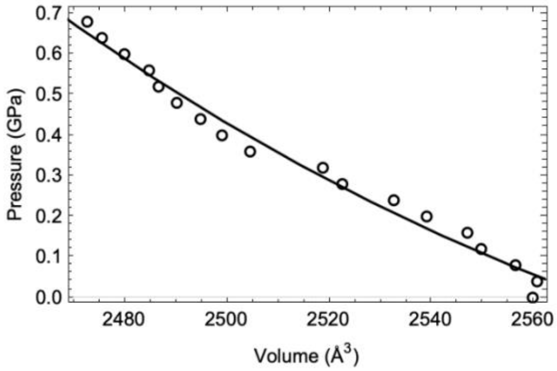

Figure S32. Bulk modulus fit for **NdHOTP**. B_0 = 11.9 ± 3.2 GPa, B_0' = 18.5 ± 14.3 GPa, V_0 = 2571.7 ± 5.3 Å^3.

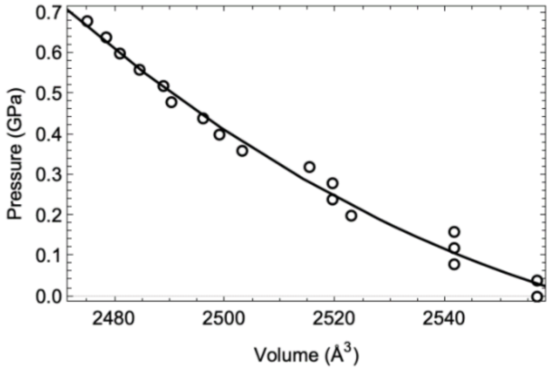

Figure S33. Bulk modulus fit for **PrHOTP**. B_0 = 9.9 ± 3.4 GPa, B_0' = 46.2 ± 26.2 GPa, V_0 = 2564.5 ± 5.5 Å^3.

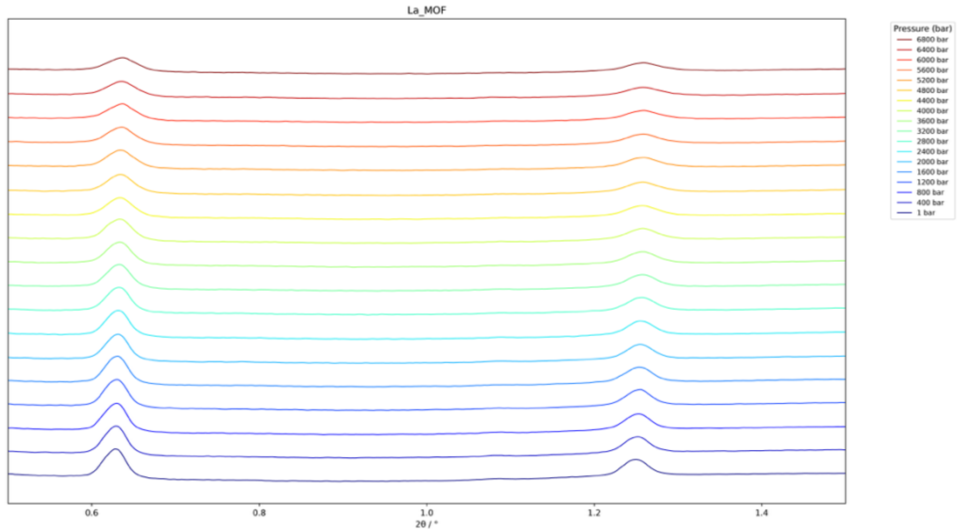

Figure S34. Variable pressure powder X-ray diffraction of **LaHOTP**.

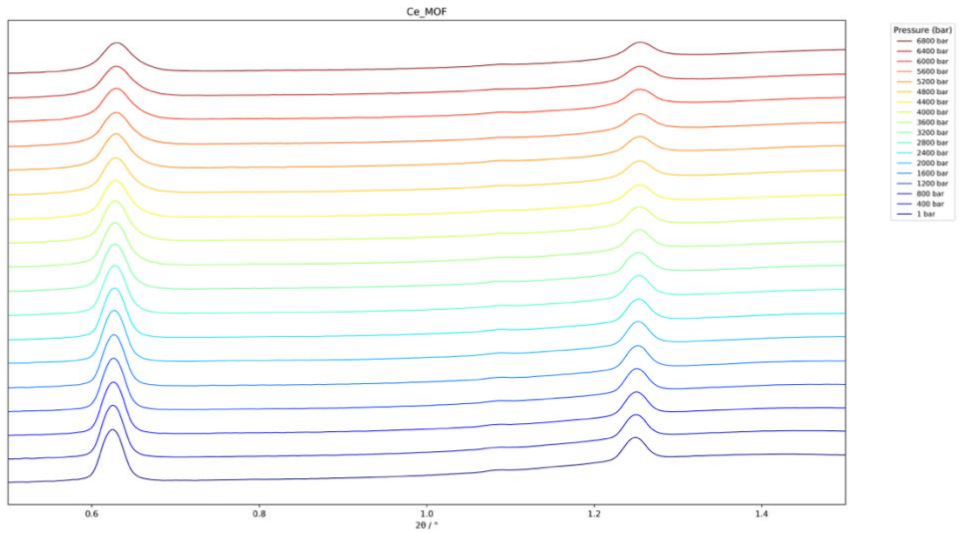

Figure S35. Variable pressure powder X-ray diffraction of **CeHOTP**.

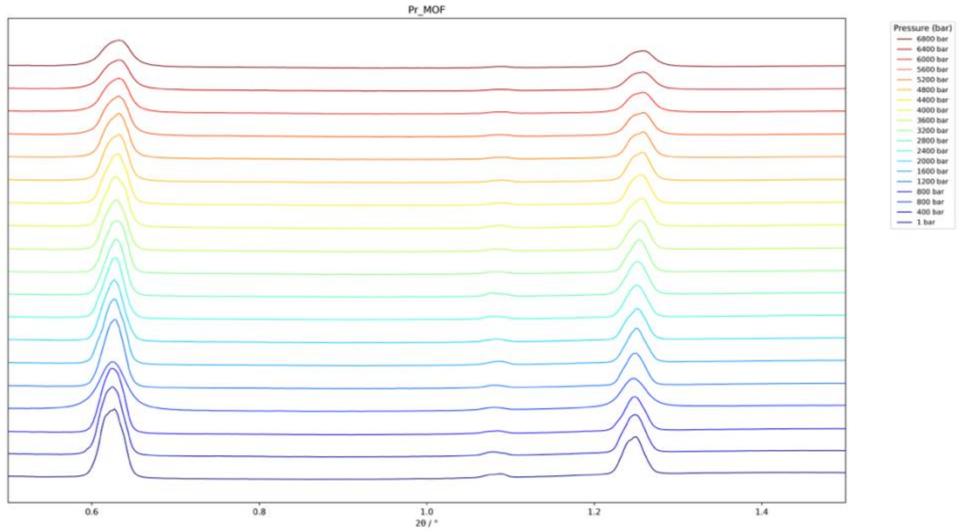

Figure S36. Variable pressure powder X-ray diffraction of **PrHOTP**.

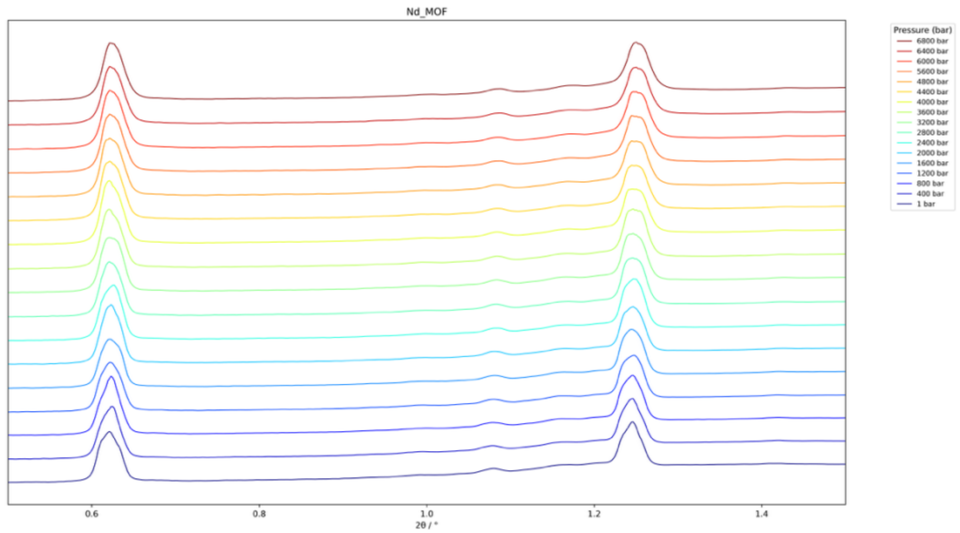

Figure S37. Variable pressure powder X-ray diffraction of **NdHOTP**.

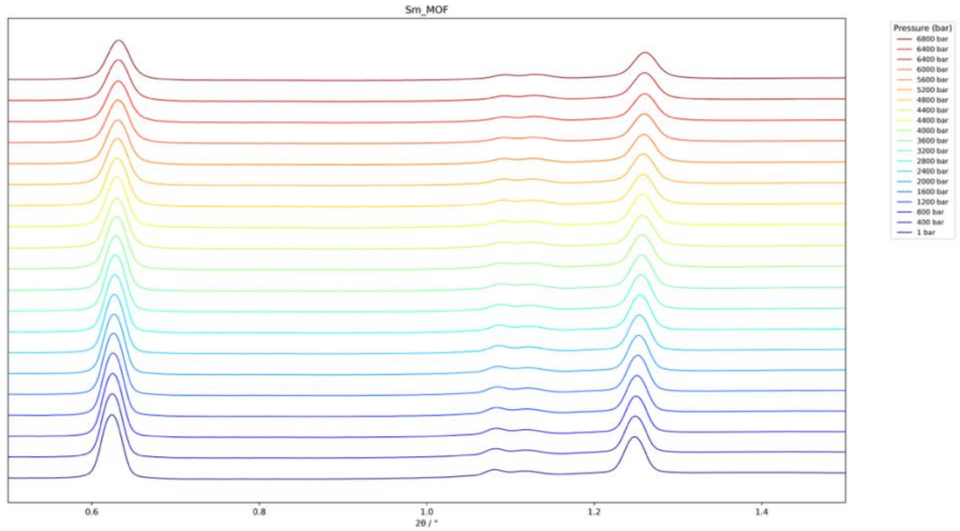

Figure S38. Variable pressure powder X-ray diffraction of **SmHOTP**.

Section S9. Construction of Hückel band structure

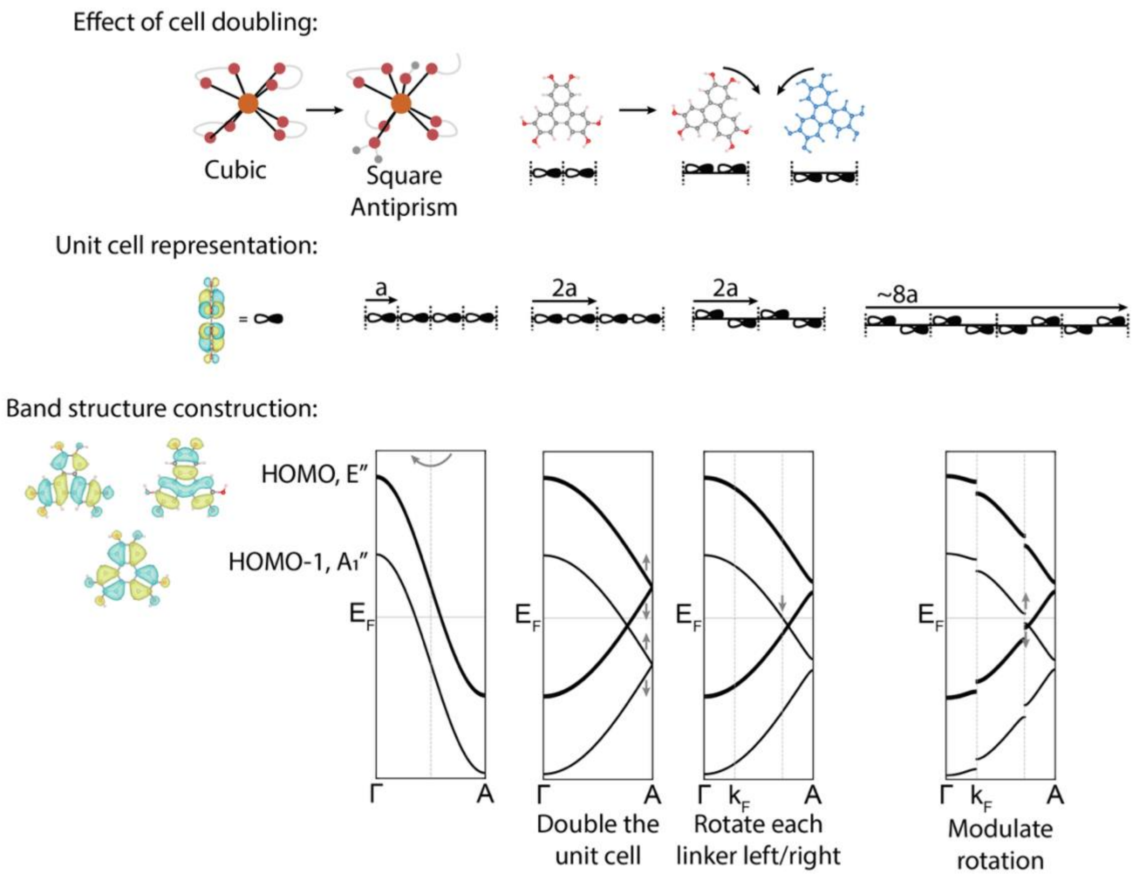

Figure S39. Construction of a Hückel band structure along the Γ to A direction. The construction begins with a 1D stack of $HOTP^{3-}$ with spacegroup *P*6*/mmm*. Only the HOMO E" and HOMO-1 A_1" orbitals from the linker are considered. The unit cell is then doubled, enabling distortion of the Ln coordination from cubic to pseudo-square antiprismatic and alternate rotation of the HOTP. This distortion lowers spacegroup to *P*6*/mcc* (a subgroup of *P*6*/mmm* with k_{index} = 2). The CDW can gap either the HOMO or HOMO-1 bands as both cross the Fermi level (here the gap is shown in the HOMO-1 band, although this is not necessarily the case).

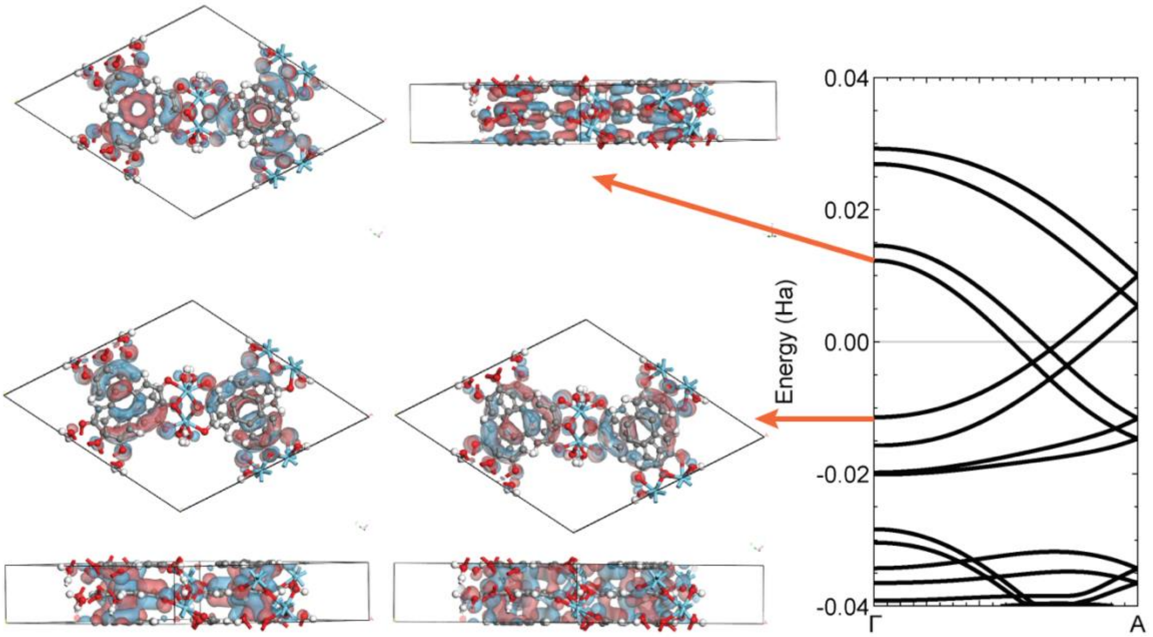

Figure S40. DFT computed band structure along the Γ to A direction as well as the orbitals for the lowest unoccupied and highest occupied orbitals at the Γ point. Note that the highest occupied orbitals are doubled degenerate.

DFT calculation. Calculations were performed on a model structure for trigonal $(La(OH)(H_2O))_3(HOTP)_2$. Hydrogen atoms were added to the average structure of **LaHOTP** to set the charge state of the linker to $HOTP^{3-}$ (i.e., one hydroxide and one water per lanthanum). The structure was geometry optimized using DFTB+ as implemented in BIOVIA Materials Studio 2020 (20.1.0.5), using dispersion correction, smearing (0.005 Ha), the PBTP (periodic table baseline parameter) set, a 1x1x3 k-point set, and with carbon atoms frozen (to preserve the relative rotation of HOTP determined by crystallography). The band structure was calculated by a single point calculation using DMol3 as implemented in BIOVIA Materials Studio 2020, suing smearing (0.005 Ha), the DN 3.5 basis set, and a 1x1x2 k-point set. The Gamma point orbitals were calculated by the same method except for a 1x1x1 k-point set. The coordinates for the optimized structure are provided below:

Model LaHOTP Structure
Unit cell: 22.1276 Å x 22.1276 Å x 6.0552 Å x 90° x 90° x 120°
Space group: *P*1

Atom	Fract x	Fract y	Fract z
O	0.52699	0.46294	0.51462
C	0.57512	0.44501	0.5018
C	0.68585	0.40969	0.48277
C	0.74333	0.39062	0.48007
O	0.43908	0.33187	0.09802
C	0.70153	0.47775	0.49984
C	0.55872	0.37165	0.48112
C	0.64735	0.49669	0.53792
H	0.75519	0.51956	0.51127
H	0.50392	0.3322	0.48211
O	0.38057	0.35006	0.56459

H	0.33138	0.3216	0.51489
O	0.46264	0.52662	0.97423
C	0.44469	0.57473	0.98738
C	0.40963	0.68569	0.00698
C	0.39074	0.7433	0.00951
O	0.33218	0.43932	0.39178
C	0.47762	0.70123	0.99019
C	0.37137	0.55846	0.00808
C	0.49644	0.647	0.95179
H	0.5195	0.75486	0.9789
H	0.33184	0.5037	0.00719
O	0.34981	0.38035	0.92598
H	0.32144	0.33117	0.97563
La	0.57078	0.5707	0.74495
H	0.37096	0.37102	0.74534
O	0.53706	0.06405	0.51462
C	0.55499	0.13011	0.5018
C	0.59031	0.27616	0.48277
C	0.60938	0.35271	0.48007
O	0.66813	0.10721	0.09802
C	0.52225	0.22378	0.49984
C	0.62835	0.18707	0.48112
C	0.50331	0.15066	0.53792
H	0.48044	0.23563	0.51127
H	0.6678	0.17172	0.48211
O	0.64994	0.03051	0.56459
H	0.6784	0.00978	0.51489
O	0.47338	0.93602	0.97423
C	0.42527	0.86996	0.98738
C	0.31431	0.72394	0.00698
C	0.2567	0.64744	0.00951
O	0.56068	0.89286	0.39178
C	0.29877	0.77639	0.99019
C	0.44154	0.81291	0.00808
C	0.353	0.84944	0.95179
H	0.24514	0.76464	0.9789
H	0.4963	0.82814	0.00719
O	0.61965	0.96946	0.92598
H	0.66883	0.99027	0.97563
La	0.4293	0.00008	0.74495
H	0.62898	0.99995	0.74534
O	0.93595	0.47301	0.51462
C	0.86989	0.42488	0.5018
C	0.72384	0.31415	0.48277
C	0.64729	0.25667	0.48007
O	0.89279	0.56092	0.09802
C	0.77622	0.29847	0.49984
C	0.81293	0.44128	0.48112
C	0.84934	0.35265	0.53792
H	0.76437	0.24481	0.51127
H	0.82828	0.49608	0.48211
O	0.96949	0.61943	0.56459
H	0.99022	0.66862	0.51489
O	0.06398	0.53736	0.97423
C	0.13004	0.55531	0.98738
C	0.27606	0.59037	0.00698
C	0.35256	0.60926	0.00951
O	0.10714	0.66782	0.39178
C	0.22361	0.52238	0.99019
C	0.18709	0.62863	0.00808
C	0.15056	0.50356	0.95179
H	0.23536	0.4805	0.9789
H	0.17186	0.66816	0.00719
O	0.03054	0.65019	0.92598
H	0.00973	0.67856	0.97563
La	0.99992	0.42922	0.74495
H	0.00005	0.62904	0.74534
O	0.53706	0.47301	0.01462
C	0.55499	0.42488	0.0018
C	0.59031	0.31415	0.98277
C	0.60938	0.25667	0.98007
O	0.66813	0.56092	0.59802
C	0.52225	0.29847	0.99984
C	0.62835	0.44128	0.98112
C	0.50331	0.35265	0.03792
H	0.48044	0.24481	0.01127

H	0.6678	0.49608	0.98211
O	0.64994	0.61943	0.06459
H	0.6784	0.66862	0.01489
O	0.47338	0.53736	0.47423
C	0.42527	0.55531	0.48738
C	0.31431	0.59037	0.50698
C	0.2567	0.60926	0.50951
O	0.56068	0.66782	0.89178
C	0.29877	0.52238	0.49019
C	0.44154	0.62863	0.50808
C	0.353	0.50356	0.45179
H	0.24514	0.4805	0.4789
H	0.4963	0.66816	0.50719
O	0.61965	0.65019	0.42598
H	0.66883	0.67856	0.47563
La	0.4293	0.42922	0.24495
H	0.62898	0.62904	0.24534
O	0.93595	0.46294	0.01462
C	0.86989	0.44501	0.0018
C	0.72384	0.40969	0.98277
C	0.64729	0.39062	0.98007
O	0.89279	0.33187	0.59802
C	0.77622	0.47775	0.99984
C	0.81293	0.37165	0.98112
C	0.84934	0.49669	0.03792
H	0.76437	0.51956	0.01127
H	0.82828	0.3322	0.98211
O	0.96949	0.35006	0.06459
H	0.99022	0.3216	0.01489
O	0.06398	0.52662	0.47423
C	0.13004	0.57473	0.48738
C	0.27606	0.68569	0.50698
C	0.35256	0.7433	0.50951
O	0.10714	0.43932	0.89178
C	0.22361	0.70123	0.49019

C	0.18709	0.55846	0.50808
C	0.15056	0.647	0.45179
H	0.23536	0.75486	0.4789
H	0.17186	0.5037	0.50719
O	0.03054	0.38035	0.42598
H	0.00973	0.33117	0.47563
La	0.99992	0.5707	0.24495
H	0.00005	0.37102	0.24534
O	0.52699	0.06405	0.01462
C	0.57512	0.13011	0.0018
C	0.68585	0.27616	0.98277
C	0.74333	0.35271	0.98007
O	0.43908	0.10721	0.59802
C	0.70153	0.22378	0.99984
C	0.55872	0.18707	0.98112
C	0.64735	0.15066	0.03792
H	0.75519	0.23563	0.01127
H	0.50392	0.17172	0.98211
O	0.38057	0.03051	0.06459
H	0.33138	0.00978	0.01489
O	0.46264	0.93602	0.47423
C	0.44469	0.86996	0.48738
C	0.40963	0.72394	0.50698
C	0.39074	0.64744	0.50951
O	0.33218	0.89286	0.89178
C	0.47762	0.77639	0.49019
C	0.37137	0.81291	0.50808
C	0.49644	0.84944	0.45179
H	0.5195	0.76464	0.4789
H	0.33184	0.82814	0.50719
O	0.34981	0.96946	0.42598
H	0.32144	0.99027	0.47563
La	0.57078	0.00008	0.24495
H	0.37096	0.99995	0.24534

Section S10. References

(1) Sheldrick, G. M. SHELXT – Integrated Space-Group and Crystal-Structure Determination. *Acta Cryst A* **2015**, *71* (1), 3–8. https://doi.org/10.1107/S2053273314026370.

(2) Sheldrick, G. M. Crystal Structure Refinement with SHELXL. *Acta Cryst C* **2015**, *71* (1), 3–8. https://doi.org/10.1107/S2053229614024218.

(3) Müller, P. Practical Suggestions for Better Crystal Structures. *Crystallography Reviews* **2009**, *15* (1), 57–83. https://doi.org/10.1080/08893110802547240.

(4) Petříček, V.; Palatinus, L.; Plášil, J.; Dušek, M. Jana2020 – a New Version of the Crystallographic Computing System Jana. *Zeitschrift für Kristallographie - Crystalline Materials* **2023**, *238* (7–8), 271–282. https://doi.org/10.1515/zkri-2023-0005.

(5) Palatinus, L.; Chapuis, G. SUPERFLIP – a Computer Program for the Solution of Crystal Structures by Charge Flipping in Arbitrary Dimensions. *J Appl Cryst* **2007**, *40* (4), 786–790. https://doi.org/10.1107/S0021889807029238.

(6) Palatinus, L. Ab Initio Determination of Incommensurately Modulated Structures by Charge Flipping in Superspace. *Acta Cryst A* **2004**, *60* (6), 604–610. https://doi.org/10.1107/S0108767304022433.

(7) Proffen, T.; Neder, R. B. DISCUS: A Program for Diffuse Scattering and Defect-Structure Simulation. *J Appl Cryst* **1997**, *30* (2), 171–175. https://doi.org/10.1107/S002188989600934X.

(8) Alemany, P.; Casanova, D.; Alvarez, S.; Dryzun, C.; Avnir, D. Continuous Symmetry Measures: A New Tool in Quantum Chemistry.

(9) Casanova, D.; Llunell, M.; Alemany, P.; Alvarez, S. The Rich Stereochemistry of Eight-Vertex Polyhedra: A Continuous Shape Measures Study. *Chemistry – A European Journal* **2005**, *11* (5), 1479–1494. https://doi.org/10.1002/chem.200400799.

(10) Skorupskii, G.; Le, K. N.; Cordova, D. L. M.; Yang, L.; Chen, T.; Hendon, C. H.; Arguilla, M. Q.; Dincă, M. Porous Lanthanide Metal–Organic Frameworks with Metallic Conductivity. *Proceedings of the National Academy of Sciences* **2022**, *119* (34), e2205127119. https://doi.org/10.1073/pnas.2205127119.

(11) Shen, G.; Wang ,Yanbin; Dewaele ,Agnes; Wu ,Christine; Fratanduono ,Dayne E.; Eggert ,Jon; Klotz ,Stefan; Dziubek ,Kamil F.; Loubeyre ,Paul; Fat'yanov ,Oleg V.; Asimow ,Paul D.; Mashimo ,Tsutomu; Wentzcovitch ,Renata M. M.; and. Toward an International Practical Pressure Scale: A Proposal for an IPPS Ruby Gauge (IPPS-Ruby2020). *High Pressure Research* **2020**, *40* (3), 299–314. https://doi.org/10.1080/08957959.2020.1791107.

(12) Mao, H. K.; Xu, J.; Bell, P. M. Calibration of the Ruby Pressure Gauge to 800 Kbar under Quasi-Hydrostatic Conditions. *Journal of Geophysical Research: Solid Earth* **1986**, *91* (B5), 4673–4676. https://doi.org/10.1029/JB091iB05p04673.

(13) Brooks, N. J.; Gauthe, B. L. L. E.; Terrill, N. J.; Rogers, S. E.; Templer, R. H.; Ces, O.; Seddon, J. M. Automated High Pressure Cell for Pressure Jump X-Ray Diffraction. *Review of Scientific Instruments* **2010**, *81* (6), 064103. https://doi.org/10.1063/1.3449332.

(14) Lertkiattrakul, M.; Evans, M. L.; Cliffe, M. J. PASCal Python: A Principal Axis Strain Calculator. *Journal of Open Source Software* **2023**, *8* (90), 5556. https://doi.org/10.21105/joss.05556.
(15) Angel, R. J.; Alvaro, M.; Gonzalez-Platas, J. EosFit7c and a Fortran module (library) for equation of state calculations. *Zeitschrift für Kristallographie - Crystalline Materials* **2014**, *229* (5), 405–419. https://doi.org/10.1515/zkri-2013-1711.
