# Supplementary figures and images for: Evidence for an Electronically Driven Charge Density Wave in a 1D Metallic MOF
Source: ACS Cent Sci. 2026 May 5;12(5):704–11. doi: 10.1021/acscentsci.6c00405 (PMC13220207; doi:10.1021/acscentsci.6c00405)

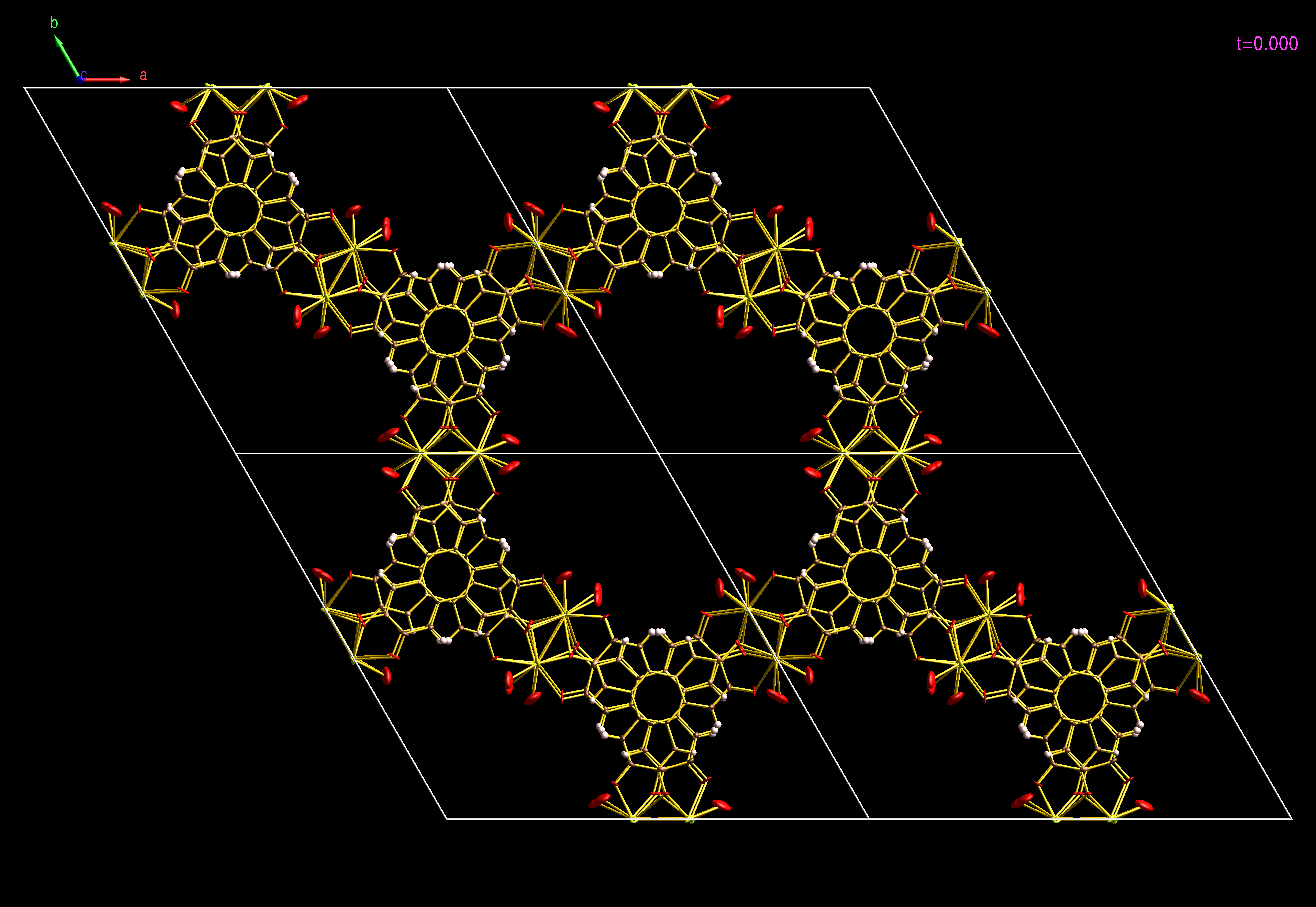

Supplement: Supplementary file 2 [file oc6c00405_si_002.gif]

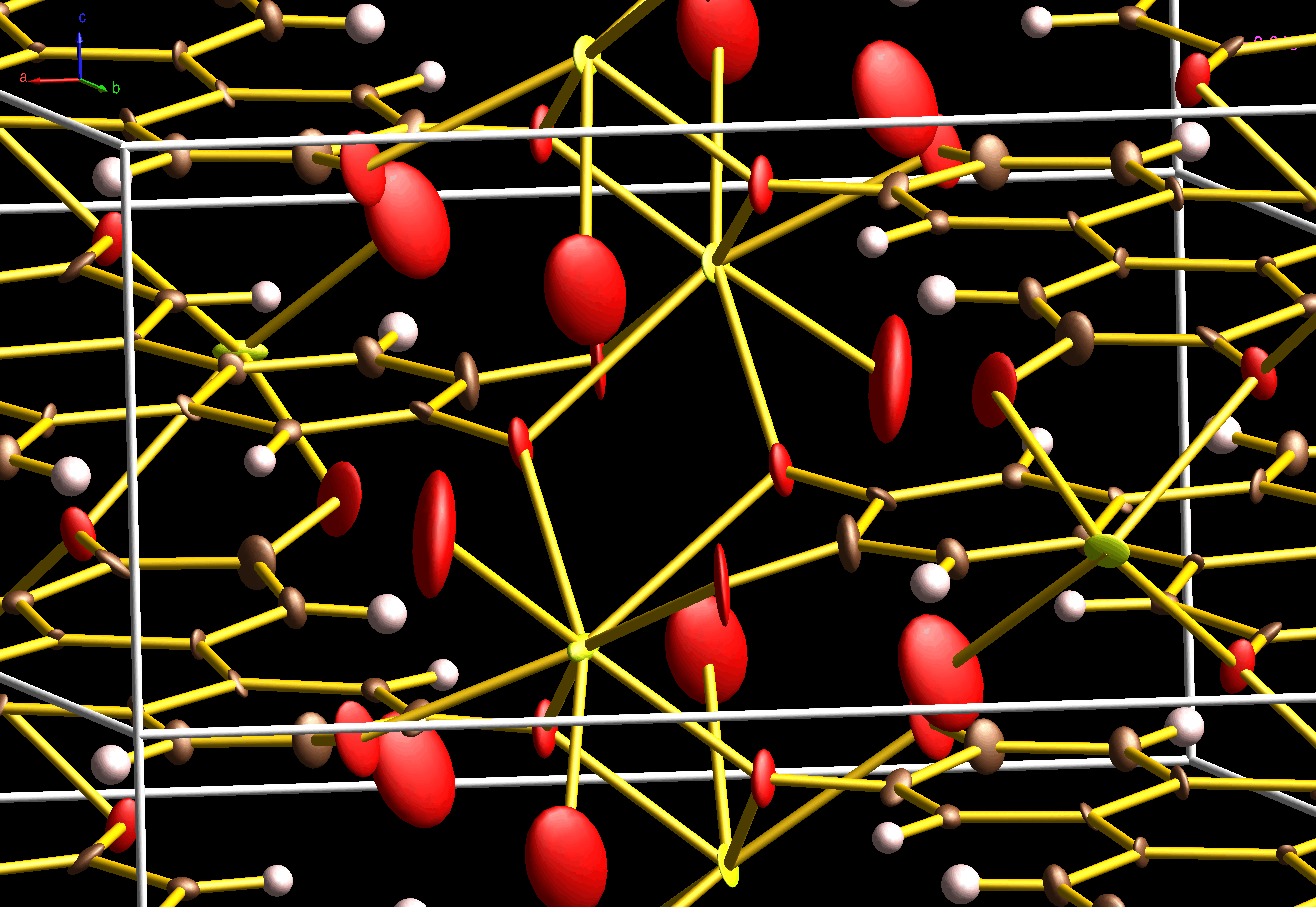

Supplement: Supplementary file 3 [file oc6c00405_si_003.gif]
